# Comparative analysis of eccDNA and circRNA tools shows increased accuracy of tool combination

**DOI:** 10.1093/gigascience/giag017

**Published:** 2026-02-25

**Authors:** Aitor Zabala, Alex M Ascensión, Iñigo Prada-Luengo, David Otaegui

**Affiliations:** Group of Neuroimmunology, Biogipuzkoa Health Research Institute, Paseo Dr. Begiristain, s/n, 20014 Donostia-San Sebastián, Spain; Group of Neuroimmunology, Biogipuzkoa Health Research Institute, Paseo Dr. Begiristain, s/n, 20014 Donostia-San Sebastián, Spain; Center for Health Data Science, Section for Health Data Science and Artificial Intelligence, Department of Public Health, Faculty of Health and Medical Sciences, University of Copenhagen, Blegdamsvej 3B, 2200 Copenhagen, Denmark; Center for Genomic Medicine, Rigshospitalet Copenhagen University Hospital, Blegdamsvej 9, 2100 Copenhagen, Denmark; Group of Neuroimmunology, Biogipuzkoa Health Research Institute, Paseo Dr. Begiristain, s/n, 20014 Donostia-San Sebastián, Spain; Center for Biomedical Research Network in Neurodegenerative Diseases (CIBER-CIBERNED-ISCIII), Av. Monforte de Lemos, 3-5. Pabellón 11. Planta 0, 28029 Madrid, Spain

**Keywords:** extrachromosomal circular DNA, circular RNA, benchmark, multi-tool integration, bioinformatics

## Abstract

**Introduction:**

Circular nucleic acids such as extrachromosomal circular DNA (eccDNA) and circular RNA (circRNA) are increasingly recognized for their biological relevance and potential as biomarkers in disease contexts. Despite their growing importance, their detection remains challenging due to tool-specific biases, limited validation frameworks, and high variability in performance across datasets.

**Methods:**

We benchmarked 10 circle detection tools across diverse conditions using both simulated and biological datasets. Our evaluation included classical performance metrics and a novel internal measure of read distribution symmetry ($\Delta$CJ) to assess circle prediction confidence. We explored the impact of sequencing protocols, filtering strategies, and combined tool consensus.

**Results:**

We found that detection accuracy was highly influenced by sequencing depth, alignment algorithm, and experimental enrichment protocols. $\Delta$CJ proved effective in flagging potential false positive circles, showing improved accuracy of *Intersect* (circles detected by all tools) and *Rosette* (circles detected by $\ge$2 tools) combinations.

**Discussion:**

This study offers a broad evaluation of circular detection tools, suggesting that the combination of $\ge$3 tools is necessary for a correct prediction. These insights will inform future experimental design and data analysis pipelines in both experimental and clinical settings.

## Introduction

Extrachromosomal circular DNA (eccDNA) and circular RNA (circRNA) are covalently closed-loop structures formed via DNA circularization events and the back-splicing process of precursor messenger RNA (pre-mRNA), respectively [1, 2]. Both types of molecules are common in eukaryotic organisms and are abundant in various types of cells and tissues [1, 3–8]. Due to their association with various diseases, their potential use as disease biomarkers has garnered significant interest [9–11]. In this study, we focus on eccDNA defined as circular DNA elements in the small-to-moderate size range (tens to a few thousand base pairs). We distinguish these from larger, tumor-associated ecDNA elements (commonly $>$10 kbp) that are typically reported in oncogene amplification studies; because ecDNA and eccDNA differ in size, biogenesis, and functional impact, we use the term eccDNA throughout to indicate the smaller class studied here.

eccDNA and circRNA isolation and detection pose several challenges. eccDNA and circRNAs are typically sequenced using targeted approaches, such as Circle-Seq, which is followed by nuclease treatments to eliminate linear sequences and enrichment processes to amplify circular sequences [12, 13]. However, targeted sequencing is currently under debate, and alternative non-enriched techniques, including ATAC-seq and RNA-seq, are gaining significance [14, 15].

The prediction of genomic coordinates of eccDNA and circRNA circular junctions (CJs)—DNA breakpoint for eccDNA and backsplice junctions (BSJs) for circRNA—requires specialized algorithms capable of identifying reads that map to that CJ. First, reads are aligned to the reference genome, and discordant reads are extracted. The unmapped reads are then remapped in reverse orientation to identify putative CJ connections. Various filtering criteria can be applied, such as the number of reads assigned to the CJ and the splicing signal that flanks the sites in the circRNA [16, 17]. For the detection of circRNA, in addition to traditional methods, a pseudo-reference alignment approach can also be employed. In this approach, circular read candidates are aligned with a synthetic reference that includes circular sequences to identify and validate BSJs [18, 19].

Numerous computational software programs have been developed and tested for detecting eccDNA and circRNA. Insights from comparisons based on eccDNA [20, 21] and circRNA [22–24] revealed significant differences in the detection capabilities of these software, particularly in terms of the total number of circles identified. Consequently, addressing the high rate of false positives (FP) caused by technical artifacts or transcripts derived from uncommon events, such as exon duplication or trans-splicing events, remains a critical challenge [12, 25]. One way to mitigate this issue is by combining 2 or more prediction software tools, identifying only the circles that are shared between them [26]. Additionally, other software works at a lower level, merging read detection results from different tools [27]. In circRNA studies, this approach defines the so-called *bona fide* circles, increasing confidence in their detection.

Regarding the establishment of common protocols for circular analysis, *nf-core* [28] provides a collection of community-driven high-quality Nextflow [29] pipelines for analyzing eccDNA [30] and circRNA [16]. These pipelines can help develop standardized protocols for detecting circular molecules. Both pipelines are compatible with targeted sequencing methods as well as whole-genome sequencing (WGS) and ATAC-seq for eccDNA and RNA-seq for circRNA.

Although these tools are widely used, studies still lack a rigorous and standardized evaluation framework, leading to considerable variability in their results. This inconsistency is largely due to the fact that most benchmarks rely solely on *in silico* data, which often produces divergent outcomes. These discrepancies arise from (1) sequencing artifacts, (2) differences in how circles are formed in repetitive regions—leading to higher FP rates, and (3) biological post-processing steps that cannot be fully replicated computationally [24, 31]. The reliance on *in silico* data is primarily due to the absence of a reliable proxy for assessing circle detection quality. As a result, most benchmarks simply compare detection outputs across tools and conditions, which is insufficient for evaluating performance on real biological data.

In this study, we present a comprehensive evaluation of 5 eccDNA and 5 circRNA detection tools using both *in silico* datasets with an array of coverages and circle sizes, as well as biological datasets produced with different methods. We show that moderate coverages (×10–×20), combined with split-read filters, minimize FP circles. To overcome the limitations of individual detection tools, we propose the *Rosette* combination, which retains only those circles supported by at least 2 tools, and achieves the optimal balance between precision and recall. To evaluate the accuracy of circle detection, we introduce the $\Delta$CJ parameter—the discrepancy in read assignment to each side of the breakpoint—as a proxy for detection quality, and validate our approach on biological datasets, thereby enhancing the reliability of circular molecule quantification.

## Methods

### eccDNA and circRNA detection software

In this study, we compared 5 eccDNA detection software—CIRCexplorer2 (v2.3.8) [32], Circle-Map (v1.1.4) [17], Circle_finder [33, 34], ecc_finder-bwa (v1.0.0), and ecc_finder-minimap2 (v1.0.0) [35]—and 5 circRNA detection software—CIRCexplorer2 (v2.3.8), circRNA_finder (v1.2) [36], CIRIquant (v2.1.0) [19], find_circ (v1.2) [8], and segemehl (v0.3.4) [37]. All of the software tools used in this study are integrated into the nf-core framework, with the exception of ecc_finder. We performed the detection of eccDNA using nf-core/circdna (v1.1.0) [30], and the detection of circRNA using nf-core/circrna (dev) [16]. Sequence reads were aligned to the human reference genome GRCh38 (NCBI) and the mouse reference genome GRCm38 (Ensembl) (Table 1).

**Table 1:** Summary of eccDNA and circRNA detection tools, versions, aligners, pipelines, and core detection strategies used in this study.

Type	Tool	Version	Aligner(s)	Pipeline	Reference	Key detection strategy
eccDNA	CIRCexplorer2	v2.3.8	BWA	nf-core/circdna v1.1.0	[38]	Detection of non-colinear alignments and reanalysis of linear junction reads to identify circular junctions
	Circle-Map	v1.1.4	BWA	nf-core/circdna v1.1.0	[17]	Identification of eccDNA breakpoints using discordant and soft-clipped reads, followed by probabilistic realignment for nucleotide-level resolution
	Circle_finder	–	BWA	nf-core/circdna v1.1.0	[33, 34]	Detection of eccDNA using paired-end information where one read maps contiguously and the mate spans a split junction
	ecc_finder-bwa	v1.0.0	BWA	standalone	[35]	Identification of circular breakpoints based on supporting discordant and split reads spanning identical boundaries
	ecc_finder-minimap2	v1.0.0	Minimap2	standalone	[35]	Same strategy as ecc_finder-bwa, using Minimap2 for read alignment
circRNA	CIRCexplorer2	v2.3.8	STAR	nf-core/circrna (dev)	[38]	Identification of back-splice junctions from non-colinear and chimeric alignments, including reanalysis of linear junction reads
	circRNA_finder	v1.2	STAR	nf-core/circrna (dev)	[36]	Direct detection of chimeric junction reads from STAR alignments, followed by filtering based on mapping quality and splice distance
	CIRIquant	v2.1.0	BWA, HISAT2	nf-core/circrna (dev)	[19]	Pseudo-reference-based detection of circRNAs using CIRI2, followed by realignment to circular references for quantification
	find_circ	v1.2	Bowtie2	nf-core/circrna (dev)	[8]	Segment-based anchor alignment approach to identify back-splice junctions with canonical splice signals
	segemehl	v0.3.4	segemehl	nf-core/circrna (dev)	[37]	Detection of chimeric and back-splice junction reads using split-read alignment supporting complex splicing patterns

For read alignment, eccDNA sequencing reads were mapped to the reference genome using BWA (v0.7.17-r1188), while circRNA sequencing reads were aligned using STAR (v2.7.11b). Alignments were performed against the human reference genome GRCh38 (NCBI) and the mouse reference genome GRCm38 (UCSC).

Although all software detects circles based on identifying the CJ, their strategies for circle identification differ.

#### eccDNA

CIRCexplorer2 is the upgraded version of CIRCexplorer [38]. It was primarily developed to detect circRNA, but it can also identify eccDNA. CIRCexplorer2 integrates additional aligner options beyond the original TopHat2 [39], including STAR [40], MapSplice [12], and segemehl, to accommodate different RNA-seq mapping preferences. CIRCexplorer2 aligns reads to the reference genome using various aligners and detects non-colinear reads. Next, CIRCexplorer2 analyzes these non-colinear alignments to detect the exact location of the CJ. Additionally, CIRCexplorer2 reanalyzes reads that were originally mapped to linear exon-exon junctions. For circRNA, it also performs *de novo* assembly of linear reads to discover novel exons and splicing events. Additionally, unmapped reads are realigned to capture any missed circular structures. In this study, DNA was mapped using BWA [41], and RNA was mapped using STAR. For *in silico* circRNA detection, we selected an intermediate non-annotated file due to the lack of detected circRNAs in the annotated output generated by the nf-core CIRCexplorer module.

Circle-Map identifies eccDNA breakpoints by utilizing discordantly mapped reads and mapping soft-clips using probabilistic models. First, Circle-Map detects eccDNA candidate reads, including discordant read pairs, soft-clipped reads, and hard-clipped reads, using the BWA aligner. Next, it constructs a breakpoint graph based on these candidate reads. Finally, soft-clipped reads are realigned using a probabilistic model to accurately determine the eccDNA breakpoints and achieve nucleotide-level resolution.

Circle_finder is designed to identify eccDNA from WGS and ATAC-seq data by analyzing read pairs using BWA. It collects all read pairs where one read maps uniquely to the genome in a contiguous manner and the other read maps as a split read flanking the mapped reads. The start of the split read and the end of the contiguous read are then annotated as the start and end points of the eccDNA.

ecc_finder detects circular breakpoints based on discordant reads and split reads. Once discordant reads and split reads are identified, only reads spanning the same boundary are retained to define the breakpoint. By default, ecc_finder uses the BWA aligner, but Minimap2 [42] can also be used.

#### circRNA

circRNA_finder uses STAR to directly identify chimeric junction reads from RNA-Seq data. After the initial alignment, the algorithm filters these chimeric reads to detect potential CJs based on predefined criteria. The filtering process includes evaluating the uniqueness of the mapped reads, allowing for a limited number of mismatches, and ensuring that the distance between splice donor and acceptor sites is within a specified range.

CIRIquant extends the functionality of CIRI2 by implementing a pseudo-reference-based approach for circRNA detection. Initially, reads are aligned to the reference genome using BWA, and unmapped reads are considered as potential circRNA candidates. CIRIquant then uses CIRI2 to detect circRNAs, but it also supports BSJ bed files created by other software. A pseudo-reference consisting of circular sequences is generated, and all circular candidate reads are aligned to the pseudo-reference using HISAT2. Reads that map concordantly within a 10 bp region of the BSJ are classified as circular reads. Additionally, CIRIquant can perform RNase correction, linear RNA quantification, and circRNA differential expression analysis.

find_circ uses a segment-based approach to identify circRNA. First, reads are mapped to the reference genome using Bowtie2, and reads that align contiguously are discarded. From the remaining reads, 20 nucleotides from both ends are extracted and aligned to obtain unique anchor positions within spliced exons. Anchors that align in the reverse orientation are identified as circRNAs. The anchor alignments are then extended to the BSJ, flanked by GU/AG splice sites.

Segemehl is able to identify multiple types of splice junctions. It aligns RNA-Seq reads to a reference genome while accounting for complex splicing patterns that are characteristic of circRNAs. The software detects chimeric reads that span back-splice junctions, where the 3′ end of one exon is joined to the 5′ end of another in a circular fashion.

### *In silico* datasets

#### CircleSim

*CircleSim* [43] is a specific simulation software for circular and linear reads. It is implemented in Python 3 and consists of 3 modules: (1) *coordinates*: generates coordinates based on a provided length distribution; (2) *reads*: simulates circular or linear short-read sequencing; and (3) *join*: merges circular and linear FASTQ files.

The *coordinates* module selects a chromosome at random based on length-associated probabilities. For both DNA and RNA, the first nucleotide of the CJ is randomly chosen across the entire genome and transcriptome, respectively. The position of the second nucleotide is determined by the length of the circular region, which can be modeled using either a uniform or a lognormal distribution. The uniform distribution is defined by specified minimum and maximum lengths, while the lognormal distribution is characterized by its mean and standard deviation, with options to set minimum and maximum lengths as well.

The *reads* module simulates sequencing based on the read and insert lengths, and a coverage defined as


\begin{eqnarray*}
\mathrm{coverage} = \frac{{n\mathrm{\_reads}} \cdot \mathrm{reads\_length} \cdot 2}{\mathrm{circle\_length}}.
\end{eqnarray*}

For circular molecules, a nucleotide within the circle is selected randomly, and the distance to the CJ determines whether the read is concordant, discordant, or a split read. Concordant reads are those mapped in the expected orientation, discordant reads are mapped in the opposite orientation, and split reads have an unmapped portion because they span across the CJ. *CircleSim* includes an option to increase the proportion of reads near the CJ. For linear molecules, the start of the read is randomly selected between the start of the sequence and the position defined as the end of the sequence minus the insert length, ensuring that all reads are concordant.

Despite its relevance for proper *in silico* analysis, *CircleSim* faces several limitations related to circle generation dynamics. Our main assumption is that circles are uniformly distributed across the genome or transcriptome; however, biological eccDNA and circRNA formation is known to be biased by chromatin state, transcriptional activity, repetitive elements, and genomic architecture [44–47].

The source code is released under the MIT license and is freely available.

#### *In silico* datasets

We used *CircleSim* (v1.0.0) to generate 1,000 eccDNAs and circRNAs from the canonical chromosomes and transcripts for the GRCh38.p14 version (NCBI) of the human genome, respectively. The circles were simulated using a log-normal size distribution implemented in the $scipy.stats.lognorm$ function with parameters *s* = 1, loc = 0, and scale = 1,000 and circle lengths ranging from 175 to 10,000 bp. The reads were simulated based on short-read sequencing with a read length of 150 bp, an insert length of 500 bp, a sequencing error rate of 0.001, and a mutation rate of 0.01 under the Kimura mutation model, with coverage depths of ×5, ×7, ×10, ×15, ×20, ×30, ×50, ×70, and ×100.

### Biological datasets

We downloaded a dataset from human muscle tissue from SRA database (accession number SRR6315430), where eccDNA was enriched and sequenced using Circle-Seq [5]. We also used a dataset generated by The Chinese University of Hong Kong (CUHK) Circulating Nucleic Acids Research Group from a knockout mouse model with deficiencies in deoxyribonuclease 1-like 3 (DNASE1L3) from EGA database (accession number EGAS00001005873), where cell-free eccDNA (cf-eccDNA) was sequenced using ATAC-seq [48] sequencing. These 2 datasets are labeled as “Circle-Seq” and “ATAC-seq” correspondingly. For circRNA, we downloaded 2 HELA datasets [49] from SRA database: an original sample (accession number SRR1637090) and the corresponding sample after RNase R treatment (accession number SRR1636986), labeled “RNase(-)” and “RNase(+).” We used “prefetch” and “fasterq-dump” from the SRA Toolkit to download and convert the SRA files to FASTQ format.

The number of reads associated with each dataset is the following: Circle-Seq − 12,829,402; ATAC-seq − 12,287,079; RNase(−) − 35,685,310; RNase(+) − 23,505,713.

### Circle filtering strategies

To improve the reliability of predicted circular elements, we implemented 4 filtering strategies: *unfilter, filter-split, filter-duplicates*, and *filter*. These strategies aim to reduce FPs and emphasize consistently detected circles across different algorithms. The filtering strategies used in this study are described below:

- *unfilter*: includes all raw detections without applying any filtering criteria.
- *filter-split*: retains only circles supported by at least 2 split reads.
- *filter-duplicates*: removes overlapping circles. Two circles are considered overlapping if their coordinate-defined regions share at least 1 base. Among overlapping circles, the one with the highest number of supporting split reads is retained. If split read information is unavailable, the longest circle is kept.
- *filter*: applies both *filter-split* and *filter-duplicates*. Specifically, it first retains circles with at least 2 supporting split reads, and then removes overlapping circles as described above.

All tools used in this study provide split-read information, except for *CIRCexplorer2* in the context of *in silico* circRNA detection, in which split information is not considered.

### Circle combination strategies

**Figure 1:**
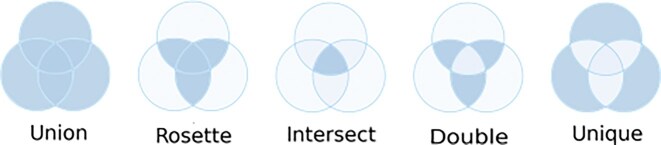
Description of the combining strategies. Visualization of the different combining strategies: *Union, Rosette, Intersect, Double*, and *Unique*.

To assess the reliability of circle detection across multiple tools, we evaluated the performance of different tool combination strategies (Fig. 1):

- *Rosette*: includes circles detected by at least 2 different tools in a combination of 3 or more tools.
- *Union*: includes all circles detected by any of the tools.
- *Intersect*: includes only circles detected by all tools in the set.
- *Unique*: includes circles detected by only 1 tool.
- *Double*: includes circles detected by at least 2 tools, excluding both *Unique* and *Intersect* detections.

The combinations shown in the main plots are based on sets of 3 or more tools, in accordance with the minimum requirement of 3 tools for the *Rosette* strategy. However, 2 tool combinations for *Union, Intersect*, and *Unique* are included in the Supplementary Material for completeness.

### Statistical analysis of *in silico* data

#### Base metrics

A threshold of 20 bp between the analyzed circles was applied to determine if 2 circles were considered the same. Detection accuracy in simulated data was analyzed using precision, recall, and *F*-score metrics.


\begin{eqnarray*}
{\mathrm{ Precision}} = \frac{\mathrm{ TP}}{\mathrm{ TP }+ \mathrm{ FP}},
\end{eqnarray*}

\begin{eqnarray*}
\mathrm{Recall} = \frac{\mathrm{ TP}}{\mathrm{ TP }+ \mathrm{ FN}},
\end{eqnarray*}

\begin{eqnarray*}
{F\mathrm{-score}} = \frac{2 \cdot \mathrm{Precision} \cdot \mathrm{Recall}}{\mathrm{Precision} + \mathrm{Recall}},
\end{eqnarray*}

where TP = true positives, FP = false positives, and FN = false negatives.

#### CJ detection precision

To evaluate the offset of the CJ detection, we calculated the ratio of the number of circles detected with an offset of 1 bp ($\mathrm{eccDNA}_{\mathrm{T=1}}$ for eccDNA and $\mathrm{circRNA}_{\mathrm{T=1}}$ for circRNA) and the maximum number of circles detected ($\mathrm{eccDNA}_{\mathrm{max}}$ for eccDNA and $\mathrm{circRNA}_{\mathrm{max}}$ for circRNA). Higher ratios indicate that most circles are detected with only a small offset of 1 bp, while lower ratios indicate that a higher offset is required.

#### Length distribution

The distribution of eccDNA and circRNA lengths was analyzed and compared using the Kolmogorov–Smirnov (KS) test. To account for the non-uniformity of the distributions, we first examined the full range of circle lengths (175–10,000 bp). We then focused specifically on short circles, defined as those within the 175–1,000 bp range. Finally, we identified a specific length interval, referred to as the “square” range (shown in Fig. 4), where the observed distribution was lower compared to the simulated distribution. The circular length distribution was plotted using both absolute and relative counts. The relative counts were calculated by normalizing against the counts from the simulated data. To enhance the clarity of the distribution plots, a sliding window of size 5 was applied to smooth the distribution curves. This technique allowed for more precise visualization of the trends across different tools, facilitating the comparison between predicted lengths and the ground truth, represented by the simulated circles. For this analysis, we used *in silico* datasets generated at ×30 coverage, ensuring consistency across comparisons.

#### Repeat element annotation

For the repeat element analysis, we utilized the GRCh38 (NCBI) genome assembly in conjunction with RepeatMasker open (v4.0.5) and the Repeat Library (31 January 2014). The RepeatMasker software, using the specified library, allowed us to identify and classify repetitive elements within the genome. Repeat elements were categorized into several classes, including *LINEs* (Long Interspersed Nuclear Elements), *SINEs* (Short Interspersed Nuclear Elements), *DNA* (DNA transposons), *satellite* (satellite DNA), and *other* elements such as *Long Terminal Repeats (LTR), simple repeats, low-complexity regions, small nuclear RNA* (*snRNA*), and *unknown* elements. Junctions that did not overlap with any annotated repeat were labeled as *non-repetitive* (Ø). For this analysis, we used *in silico* datasets generated at ×30 coverage, ensuring consistency across comparisons. This analysis was performed to ensure comprehensive annotation of repeat sequences at each coordinate of the CJ. For this analysis, we used *in silico* datasets generated at ×30 coverage.

#### Genomic element annotation

For the genomic element analysis, we used the GFF annotation file corresponding to the GRCh38 version (NCBI) of the human genome. This analysis provides a comprehensive annotation of genomic elements at each CJ coordinate. Coordinates were annotated based on detailed genomic features, including *3′-UTR* (*3′ Untranslated region*), *5′-UTR* (*5′ Untranslated region*), and *other* elements such as *start codon, stop codon*, and *selenocysteine positions*. Coordinates not annotated in this step were evaluated for overlap with *exon* regions. Those that still remained unannotated were checked against *intronic* regions. Finally, any coordinates not matching any of the above categories were retained as *intergenic*. For this analysis, we used *in silico* datasets generated at ×30 coverage.

### Statistical analysis of biological data

#### Similarity analysis

eccDNA and circRNA detection results were visualized using UpSet plots [50], which allow visualization of overlapping circles defined with a threshold of 20 bp.

#### CJ difference ($\Delta \mathrm{CJ}$)

When a read spans the CJ, the split, unmapped portion of the read can be associated with either the left or right side of the junction. Assuming both sides are equally likely, a strong imbalance in read association suggests potential artifacts, as the supporting reads may originate from other circles or misalignments.

Based on this hypothesis, if *N* is the number of reads spanning a circle’s CJ, $k_L$ is the number assigned to the left side, and $k_R = N - k_L$ to the right side, we define the metric $\Delta \mathrm{CJ}$ as


\begin{eqnarray*}
\Delta \mathrm{CJ} = \frac{|k_L - k_R|}{N}.
\end{eqnarray*}

To obtain $k_L$ and $k_R$, we extracted reads overlapping windows centered on the circle start and end positions, respectively. Each window extended $N_{\mathrm{offset}} = 20$ nucleotides upstream and downstream of the junction coordinate to capture all potentially spanning reads. Unique read identifiers were collected within these windows from the aligned BAM file using the pysam package for efficient read retrieval. The total number of CJ reads, *N*, corresponds to the union of reads found in both windows.

To improve robustness against alignment artifacts, $\Delta \mathrm{CJ}$ integrates 2 complementary corrections: junction region mappability bias and read-level mapping quality weighting.

- *Junction region mappability*: The assumption of equal mapping probability (*p* = 0.5) for both sides of the junction does not hold when local sequence mappability differs. To account for this, the expected probability of left-side support was estimated from a mappability track:
  \begin{eqnarray*}
p_L = \frac{M_L}{M_L + M_R}, \quad p_R = 1 - p_L,
\end{eqnarray*}
  where $M_L$ and $M_R$ represent the average mappability scores over 30-nucleotide regions located immediately inside the circle at each junction boundary. Mappability values were extracted from UCSC 50-mer multi-track bigWig files using the pyBigWig library.
  These scores were used to set the expected probability parameter for a 2-sided binomial test assessing whether the observed left/right distribution significantly deviated from the expected mappability-driven balance.
- *Read-level mapping quality weighting*: To reduce the influence of poorly aligned or multi-mapped reads, each read *i* contributes to the counts $k_L$ or $k_R$ according to its individual mapping quality ($\mathrm{MAPQ}_i$). Each read was assigned a weight $w_i \in [0,1]$ based on the probability of correct alignment derived from its MAPQ value:
  \begin{eqnarray*}
w_i = 1 - 10^{-\frac{\mathrm{MAPQ}_i}{10}}.
\end{eqnarray*}
  This probabilistic weighting reflects the exponentially decreasing error probability encoded in MAPQ values, thus giving more nuanced confidence weights to individual reads instead of a linear approximation.
  The effective read counts were obtained by summing the weights of all reads overlapping each junction side:
  \begin{eqnarray*}
k_L = \left\lceil \sum _{i \in CJ_1} w_i \right\rceil , \quad k_R = \left\lceil \sum _{i \in CJ_2} w_i \right\rceil , \quad N = k_L + k_R,
\end{eqnarray*}
  and the junction imbalance metric was computed as
  \begin{eqnarray*}
\Delta \mathrm{CJ} = \frac{|k_L - k_R|}{N}.
\end{eqnarray*}

Values of $\Delta \mathrm{CJ}$ close to 0 indicate a balanced read distribution, whereas $\Delta \mathrm{CJ} = 1$ means that all reads associate with only one side of the junction. Of note, the ceiling function is used to approximate the number of reads to a non-zero integer, which is required for the following steps.

While informative, this metric has limitations, particularly at low *N*, where extreme $\Delta \mathrm{CJ}$ values might occur. To address this, we modeled the probability of observing a particular distribution of reads ($k_L$ and $k_R$) to identify circles where such probabilities are exceedingly low.

The number of reads associated with one side of the CJ, *X*, can be modeled using a binomial distribution: $X \sim B(N, 0.5)$ for the ideal scenario where mappability is not considered ($M_L = M_R \rightarrow p_L=p_R=0.5)$. Thus,


\begin{eqnarray*}
P(X=k) = {N\atopwithdelims ()k} \cdot 0.5^k \cdot 0.5^{N-k} = {N\atopwithdelims ()k} \cdot 0.5^N.
\end{eqnarray*}

Considering that assignment to either side of the CJ is arbitrary, the final distribution must account for symmetry:


\begin{eqnarray*}
P(X=k) &=& \left( {N\atopwithdelims ()k} + {N\atopwithdelims ()N-k} \right)\cdot 0.5^N = 2 \ {N\atopwithdelims ()k} \cdot 0.5^N,\\
&&\quad k = \lbrace 0, ..., N/2\rbrace.
\end{eqnarray*}

Thus, the cumulative probability of assigning up to *k* reads out of *N* to one side of the CJ is


\begin{eqnarray*}
p = P(X\le k) = 2 \cdot 0.5^N \sum _{i=0}^k \ {N\atopwithdelims ()i}.
\end{eqnarray*}

For even *N* values, the cumulative distribution can exceed 1 due to double-counting at the midpoint $P(X=N/2)$. For example, when $N=6$ and $k=3$, the cumulative probability is$p = 2\cdot 0.5^6 ({6 \atopwithdelims ()0} + {6 \atopwithdelims ()1} + {6 \atopwithdelims ()2} + {6 \atopwithdelims ()3}) = 1.3125$. This happens because $P(X=3)$ is counted twice. In contrast, when $N=7$, for $k=3$, $p = 2\cdot 0.5^6 ({7 \atopwithdelims ()0} + {7 \atopwithdelims ()1} + {7 \atopwithdelims ()2} + {7 \atopwithdelims ()3}) = 1$, as the complement of ${7\atopwithdelims ()3}$ is ${7 \atopwithdelims ()4}$. Thus, in even cases where $k = N/2$, the cumulative probability is set to 1 since midpoints are not relevant for identifying outliers, which are our primary interest.

For scenarios with unequal mappabilities, probabilities are calculated in a similar fashion. In this case, 2 variables arise: $X_L$, the number of $k_L$ reads, which follows a binomial distribution $B(k, p_L)$; and $X_R$, the number of $k_R$ reads, which follows a binomial distribution$B(k, p_R)$. Thus,


\begin{eqnarray*}
P(X_L=k_L) &=& {k \atopwithdelims ()k_L}p_L^{k_L}(1-p_L)^{k-k_L} \\
P(X_R=k_R) &=& {k \atopwithdelims ()k_R}p_R^{k_R}(1-p_R)^{k-k_R}.
\end{eqnarray*}

Considering that both $k_L|k_R$ and $p_L|p_R$ pairs are related; distributions associated to $X_L$ and $X_R$ are also related:


\begin{eqnarray*}
P(X_L=k_L) &=& {k \atopwithdelims ()k_L}p_L^{k_L}(1-p_L)^{k-k_L} \\
&=& \frac{k!}{k_L!(k-k_L)!}p_L^{k_L}(1-p_L)^{k-k_L} \\
&=& \frac{k!}{k_L!k_R!}p_L^{k_L}p_R^{k_R} \\
&=& \frac{k!}{(k-k_R)!k_R!}(1-p_R)^{k-k_R}p_R^{k_R} \\
&=& {k \atopwithdelims ()k_R}p_R^{k_R}(1-p_R)^{k-k_R} = P(X_R=k_R).
\end{eqnarray*}

Therefore, since $P(X_L=k_L) = P(X_R=k_R)$, the cumulative distributions $P(X_L \le k_L)$ and $P(X_R \ge k_R)$ are equivalent.

Thus, the cumulative probability of assigning up to $k_L$ reads to the left side of the CJ and up to $k_R$ reads to the right side of the CJ can be computed using only one of the sides (e.g., left):


\begin{eqnarray*}
p &=& P(X_L \le k_L) + P(X_R \ge k_R) = 2\cdot P(X_L \le k_L) \\
&=& 2\sum _{i=0}^{k_L}{k \atopwithdelims ()i}p_L^{i}(1-p_L)^{k-i}.
\end{eqnarray*}

Under the ideal mappability condition, the minimum *N* required to achieve a cumulative probability $P(X \le 1)$ below 0.05 is $P(X \le 1) < 0.05 \,\,\,\,\Rightarrow \,\,\,\, 2\cdot 0.5^N ({N \atopwithdelims ()0} + {N \atopwithdelims ()1}) = 2(N+1)\cdot 0.5^N < 0.05 \,\,\,\,\Rightarrow \,\,\,\, N >8.58$.

The solution was retrieved numerically. For $P(X\le 1) < 0.01$, the solution is $N > 11.25$.

Using these findings, we defined 3 metrics for evaluating circle detection quality: (1) the proportion of circles with $N\ge 9$, (2) $\Delta \mathrm{CJ}$ for selected circles, and (3) the proportion of circles fulfilling $p < 0.05$ based on probabilities derived from observed *k* and *N* values. For the third metric, we applied the Benjamini–Hochberg correction, designating circles with adjusted probabilities ($p_{\mathrm{adj}} < 0.05$) as significantly skewed compared to the expected baseline distribution.

## Results

### Study design

Given the variability in individual tool performance and the differences between eccDNA and circRNA detection methods, our study evaluated 5 widely used tools for eccDNA detection—CIRCexplorer2, Circle-Map, Circle_finder, ecc_finder-bwa, and ecc_finder-minimap2, as well as 5 tools for circRNA detection—CIRCexplorer2, circRNA_finder, CIRIquant, find_circ, and segemehl. Notably, all tools except ecc_finder-bwa and ecc_finder-minimap2 are incorporated into nf-core pipelines, which provide standardized, reproducible protocols for detection and analysis, ensuring fair comparisons and minimizing variability due to differences in default parameters or preprocessing. By conducting this combined evaluation, we aimed to systematically identify shared strengths and distinct limitations across these 2 circular molecule detection approaches.

The study consists of 2 separate parts making use of *in silico* and biological data. For the *in silico* analysis, we developed *CircleSim* to generate simulated data that approach biological distributions; thus generating 2 separate datasets for eccDNA and circRNA with a wide range of coverages from ×5 to ×100. Reads from both *in silico* and biological data were assessed by each tool, and reported circles were then filtered based on different criteria (described in the “Materials and methods” section), and the resulting circles were then ordered based on the combinations of tools that reported them.

For *in silico* data, we evaluated a diverse range of metrics for each individual tool, including (1) precision, recall, and *F*-score metrics, (2) variation in the coordinates reported by the tools, and (3) deviations in circle detection associated with circle length.

**Figure 2:**
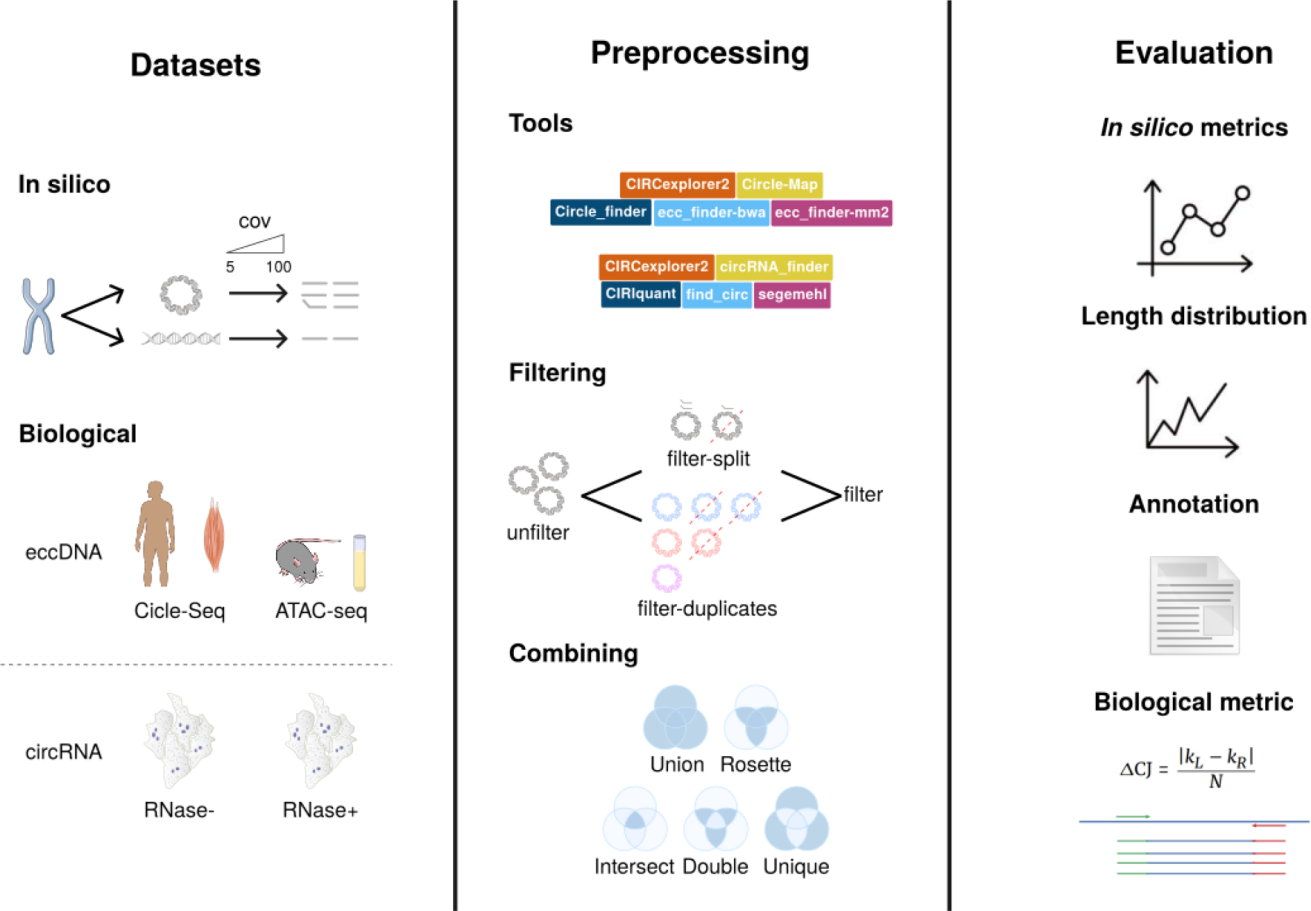
Overview of the study design and analysis workflow. The *in silico* datasets were used to evaluate detection performance, length distribution, and repeat and genomic element enrichment. Biological datasets, including Circle-Seq and ATAC-seq for eccDNA and RNA-seq (RNase−/RNase+) for circRNA, were analyzed using the same filtering and combination strategies. A novel $\Delta$CJ metric was developed to assess the consistency of CJ reads and overall circle detection quality. Icons were taken from Bioicons under CC-BY 3.0 Unported license and NIH BioArt.

For biological data, we analyzed Circle-Seq and ATAC-seq datasets for eccDNA, and RNA-seq datasets for an original sample—RNase(−)—and the corresponding sample after RNase R treatment—RNase(+)—for circRNA. We evaluated the same downstream filters as in the *in silico* data; as well as the tool combinations. Aside from circle detection patterns, we developed a new metric based on the discordance of reads assigned to the CJ split site ($\Delta$CJ) as a proxy to evaluate the “quality” of circle detection (Fig. 2).

### Circle detection evaluation in *in silico* data

#### False positive detection is biased toward high coverage

**Figure 3:**
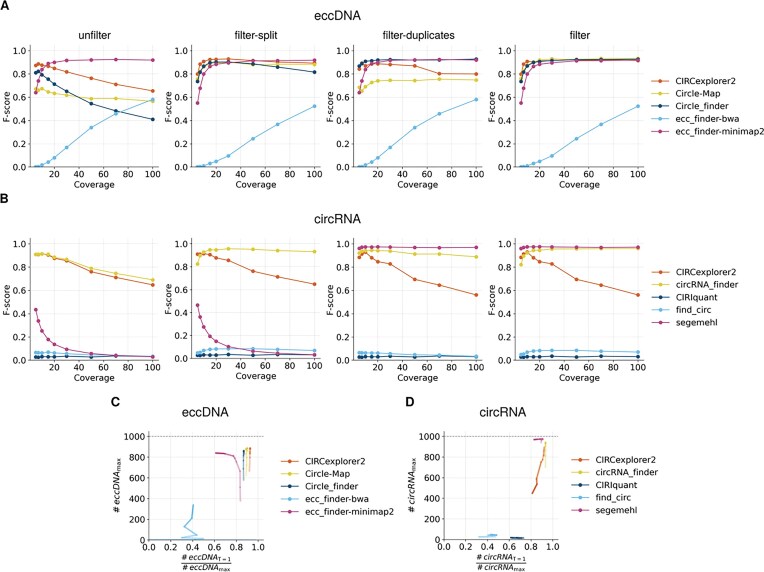
Performance analysis of detection software for eccDNA and circRNA identification in *in silico* datasets. *F*-score values for (A) eccDNA and (B) circRNA detection across 4 filtering conditions: *unfilter, filter-split, filter-duplicates*, and *filter*. Evaluation of circular predictions at coverage ×30 for (C) eccDNA and (D) circRNA based on the proportion of circles detected with an offset of 1 in the detected coordinate vs. real coordinate, relative to the total number of circles detected using *filter* data. Color intensity indicates coverage level, with higher intensity corresponding to greater coverage.

Detection accuracy for eccDNA and circRNA can be significantly affected by sequencing coverage, especially given their low abundance and the specialized methods required to identify them [51]. To investigate this relationship, we evaluated how varying sequencing coverages affect the detection accuracy of eccDNA (Fig. 3A) and circRNA (Fig. 3B). We standardized our evaluation by defining a threshold of 20 bp to determine when 2 circles should be considered identical.

Our initial results indicated that higher sequencing coverage negatively impacted overall detection performance for simulated circles, resulting in lower *F*-scores at higher coverage levels (Fig. 3A and B, *unfilter*). This decline primarily reflects a significant drop in precision, despite a modest increase in recall (Supplementary Fig. S1, *unfilter*, and Supplementary Materials 1 and 2). In other words, higher coverage allowed for the detection of more TP but also substantially increased the number of FP, ultimately reducing overall accuracy.

To address this FP detection, we evaluated 4 filtering methods (described in the “Materials and methods” section): (1) *unfilter*, (2) *filter-split*, (3) *filter-duplicates*, and (4) *filter*. Overall, we observed that applying any filter form increases the *F*-score, showing a plateau at coverages around ×10–×20. For good measure, the following analyses were performed using a ×30 coverage (Table 2).

**Table 2:** *F*-score values at coverage ×30 for eccDNA and circRNA detection tools under the different filtering conditions. Bold values indicate the highest F-score obtained by each tool across the evaluated filtering strategies.

	Tool	Unfilter	Filter-split	Filter-duplicates	Filter
eccDNA	CIRCexplorer2	0.819	**0.929**	0.882	**0.929**
	Circle-Map	0.618	0.902	0.746	**0.927**
	Circle_finder	0.652	0.903	0.924	**0.916**
	ecc_finder-bwa	0.168	0.096	**0.168**	0.096
	ecc_finder-minimap2	0.916	0.896	**0.916**	0.896
circRNA	CIRCexplorer2	0.856	**0.856**	0.829	0.829
	circRNA_finder	0.867	**0.957**	0.939	0.956
	CIRIquant	0.035	0.035	0.035	0.035
	find_circ	0.056	0.084	0.057	**0.085**
	segemehl	0.094	0.103	0.973	**0.974**

Focusing on individual filter comparison, we observed that *filter-split* was generally more effective than *filter-duplicates*, although removing duplicates sometimes enhanced the effectiveness of split read-based filtering (e.g., Circle-Map in eccDNA). Among eccDNA detection tools, CIRCexplorer2 (*F*-score = 0.929), Circle-Map (*F*-score = 0.927), Circle_finder (*F*-score = 0.916), and ecc_finder-minimap2 (*F*-score = 0.896) were the most accurate (Fig. 3A, *filter*). For circRNA detection, circRNA_finder (*F*-score = 0.956) and segemehl (*F*-score = 0.974) were identified as the most effective software (Fig. 3B, *filter*). However, segemehl was dependent on duplicate removal (*F*-score = 0.973) due to the high number of FPs, which were not adequately addressed by using only split read-based filtering (*F*-score = 0.103). It is worth noting that CIRCexplorer2 output for simulated circRNA data lacked information on reads mapped to the BSJ, which diminished the effectiveness of the split read-based filtering method. Consequently, the filtering approach only managed to filter out overlapping circles and retain longer circles. Surprisingly, find_circ (*F*-score = 0.085) and CIRIquant (*F*-score = 0.035) remained extremely inaccurate even after filtering. This effect is again driven by low recall values, although precision also dropped for find_circ with higher coverages; indicating that these 2 methods are prone to FP detection (Supplementary Fig. S1 and Supplementary Materials 1 and 2)

Therefore, these results show that high coverages may be unnecessary, if not detrimental for circle detection, incrementing the number of FP circles.

#### High coverage may affect the offset of CJ detection

Accurate CJ coordinates–defined as DNA breakpoints in eccDNA and BSJ in circRNA–are necessary for proper circle identification. Stemming from our hypothesis that circular coordinate accuracy may be affected by coverage, we analyzed how stable circle identification was with different coverages. To do this, we calculated the ratio of circles detected with an offset of 1 to the total number of detected circles using *filter*. Ratios approaching 1 indicate that most circles are accurately captured with little-to-no offset, while lower ratios indicate that higher offsets are required, and therefore circle detection is less accurate. Values of these ratios are depicted in Fig. 3C and D for eccDNA and circRNA, respectively.

In eccDNA, we observed that for CIRCexplorer2, Circle-Map, and Circle_finder, the ratio was not affected by coverage, and remained at around 0.9. On the other hand, the ratio decreased with coverage for ecc_finder-minimap2, indicating that, although more circles were detected, even if those circles were “correct” based on the *F*-score, they were detected with a higher offset from the coordinate of the simulated circle, indicating that CJ sequence was affected by some tool-related factor. Lastly, ecc_finder-bwa showed a “stabilization” of the ratio at around 0.4 with increasing coverage, but the ratio is still low.

Regarding circRNA, 2 ratio trends arise. On the one hand, tools with low overall detection (find_circ and CIRIquant) show reduced ratios; whereas tools with high accuracy (segemehl and circRNA_finder) show higher ratios above 0.8. Interestingly, CIRCexplorer2, which showed a decrease in circle detection accuracy with increased coverage, also shows a decreased ratio.

Therefore, it is clear for both eccDNA and circRNA that tools with low *F*-scores tend to show low ratios showcasing that for tools with higher FP detection values, detected circles are also inaccurately detected, with higher coordinate offsets than their counterparts.

#### Specific tools showed a circle length anomaly for short circles

**Figure 4:**
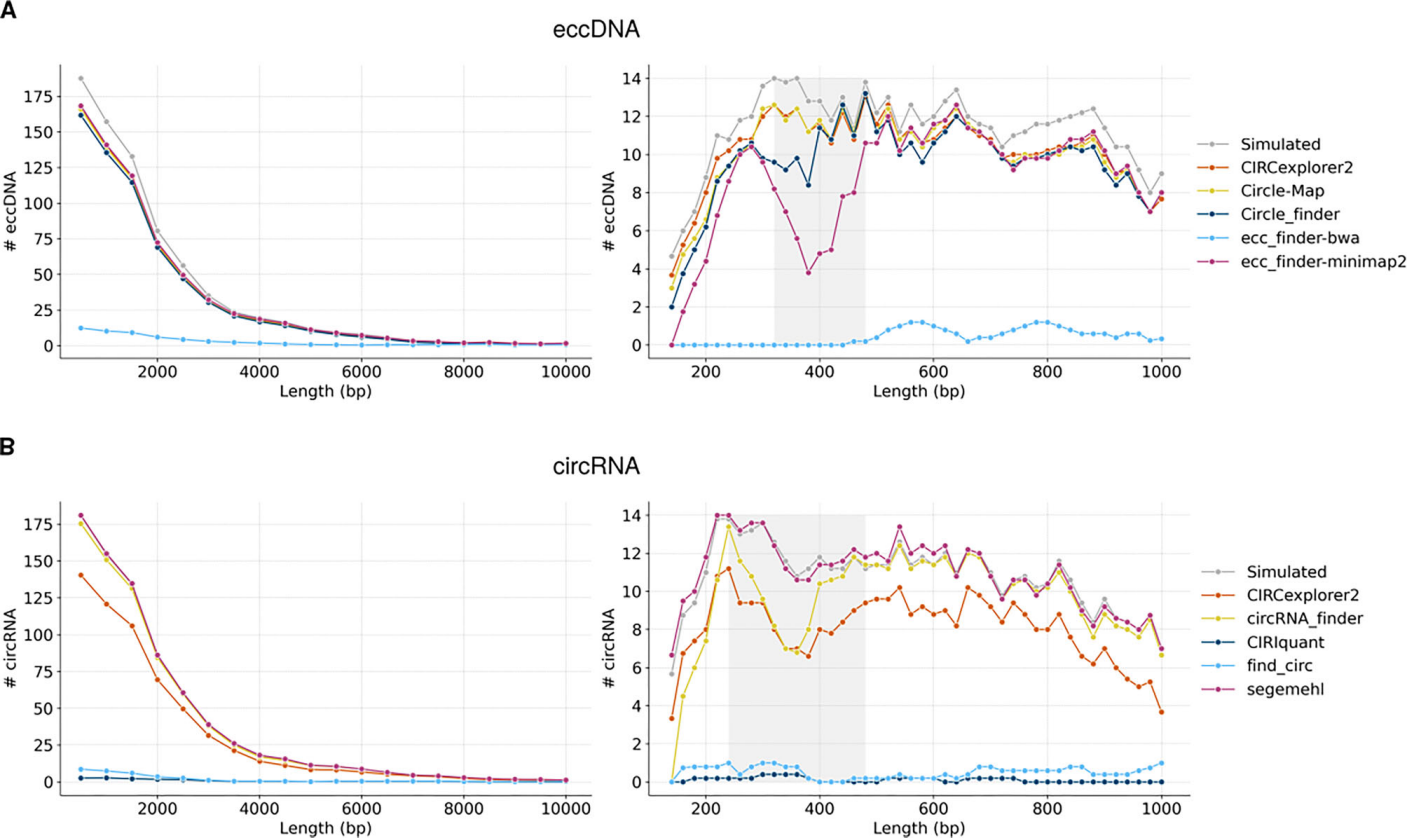
Circular length distribution in *in silico* datasets. Length distribution of detected (A) eccDNA and (B) circRNA across all size ranges (left) and within the short-length range (right) in *in silico* datasets at coverage ×30. A gray-shaded area highlights the length interval where detection performance was lowest (eccDNA: 320–480 bp; circRNA: 240–480 bp). To enhance the clarity of the distribution plots, a sliding window of size 5 was applied to smooth the distribution curves.

After observing that circle detection accuracy was variable across tools, we were interested in testing biases in the detection of circles of specific lengths. Theoretically, since *in silico* circles are generated at random positions in the genome, circle length should not pose a bias in their detection. Expectedly, tools with good circle detection accuracy showed length distributions similar to the expected distribution; whereas for tools with lower accuracies, the detection is equally reduced across length (Fig. 4 and Supplementary Fig. S2, left). This effect was present both for eccDNA (ecc_finder-bwa, KS test, KS = 0.55 and *p* = 3.97$\times 10^{-3}$) and circRNA (CIRIquant, KS = 0.75 and *p* = 9.55$\times 10^{-6}$; find_circ, KS = 0.60 and *p* = 1.12$\times 10^{-3}$).

Considering that the original circle distribution is condensed in shorter circles, we performed an additional analysis on short circles, ranging from 175 to 1,000 bp. Interestingly, a distinct reduction of detected circles occurred in the range of 320–480 bp for eccDNA (Fig. 4A and Supplementary Fig. S2A, right) and 240–480 bp for circRNA (Fig. 4B and Supplementary Fig. S2B, right). Although this reduction was more apparent for tools that already show a low circle detection, some tools with good performance showed much lower than expected counts near the 400 bp mark in eccDNA Circle_finder (KS = 0.38 and *p* = 0.66) and ecc_finde-minimap2 (KS = 0.75 and *p* = 1.87$\times 10^{-2}$) in eccDNA; and CIRCexplorer2 (KS = 0.75 and *p* = 1.87$\times 10^{-2}$) and circRNA_finder (KS = 0.50 and *p* = 0.283) in circRNA (Supplementary Material 3).

#### Tools showed a biased detection of specific repeated and genomic elements

The genome is not uniform across its sequence, having areas with specific repetitive sequences that, we hypothesize, may affect the ability to detect circles. We are also interested, specially for circRNAs, if there are notable differences in the detection depending on the genomic elements they originated from.

**Figure 5:**
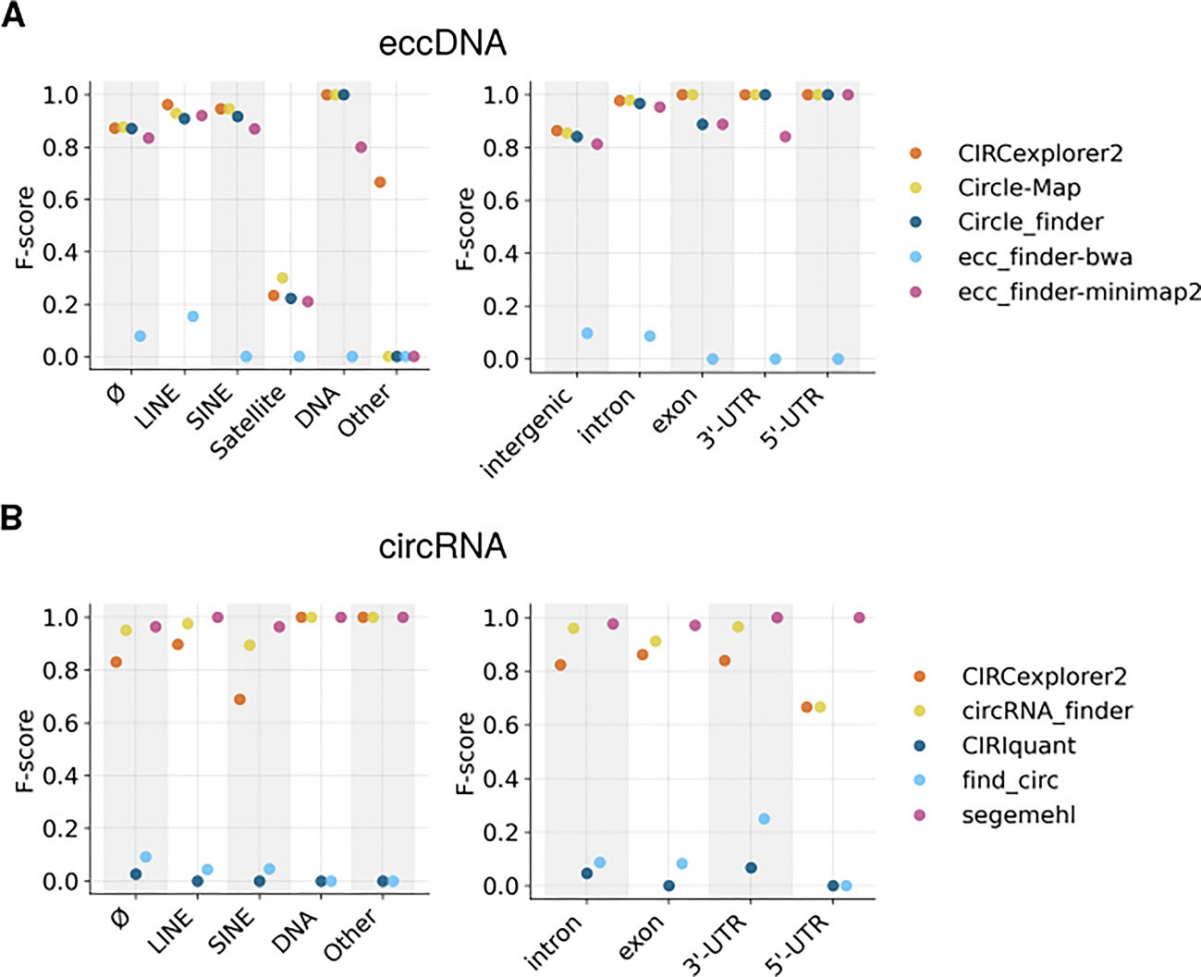
Repeat and genomic element analysis in *in silico* datasets. *F*-score values of repeat elements (left) and genomic features (right) associated with detected (A) eccDNA and (B) circRNA in *in silico* datasets at coverage ×30. In circRNAs, no intergenic circles were generated, and thus the region is not included in this plot.

Across the different genomic regions, the repeat element analysis showed the greatest limitation in the detection of satellite eccDNA. Among the 32 eccDNAs lying in satellite regions, all tools showed a lack of accuracy in detecting these circles (*F*-score $\approx$ 0.2, Fig. 5A, left). These low scores were mostly driven by a lack of recall, although 3/5 tools showed reduced precision values (0.4–0.7) showing that FP circles were also assigned to satellite regions (Supplementary Fig. S3A, left). For the rest of elements, the *F*-scores were generally high, with moderate decreases in regions without a demarcated repeating element (Ø).

There was a marked decrease in accuracy for circles detected in “other”—repetitive elements not marked within the SINE, LINE, and similar categories; however, it is important to note that only 2 eccDNAs were generated in these regions, meaning that conclusions should be drawn with caution (Supplementary Material 4).

In the case of circRNA, no satellite regions were created since they were generated from the transcriptome. Interestingly, CIRCexplorer2 and circRNA_finder exhibited a decrease in their original *F*-score in SINE regions (Fig. 5B and Supplementary igure S3B, left; and Supplementary Material 5), although this does not happen with eccDNAs.

The analysis of genomic elements showed a considerable decrease in the *F*-score for eccDNA in intergenic regions (*F*-score $\approx$ 0.8), due to a low recall. This suggests a significant limitation in the detection of eccDNA in these areas (Fig. 5A and Supplementary Fig. S3A, right; and Supplementary Material 6). In contrast, circRNA detection showed a decrease in the *F*-score specifically in the 5′UTR regions. However, it is important to note that, in this case, only 2 circRNAs were generated in these regions, so the conclusions should be interpreted with caution (Fig. 5B and Supplementary Fig. S3B, right; and Supplementary Material 7).

#### Combination of 3 or more tools improved circle detection in *in silico* data

Although individual tools may perform correctly in specific analyses, we hypothesize that the combination of several tools may improve the accuracy of circle detection, leveraging the strengths and reducing individual tool biases. This is particularly necessary for biological data, where the ground truth is unknown, and circle detection may be more difficult to perform due to unknown variation in their sequences.

Although this claim is already supported in the literature, mentioned as *bona fide* circles [26], these circles are usually defined by the combination of 2 tools. Our aim is to extend it by including 3 or more tools, under the assumption that the inclusion of more tools may increase the credibility of the detection of common circles. We evaluated the performance of software combinations using 5 different strategies (described in the “Materials and methods” section): *Union, Rosette, Intersect, Double*, and *Unique*. Briefly, *Union* is the set of all circles; *Instersect* is of circles detected by all tools, while *Unique* refers to circles detected by one tool. *Rosette* is defined as the set of circles detected by 2 or more tools; whereas *Double* includes circles detected by 2 or more tools, excluding *Intersect* circles. *Double* circles are used as a comparison to *Rosette* to see the effect of a more lenient detection of circles. Each strategy is evaluated with 16 tool combinations across the 4 filtering methods.

**Figure 6:**
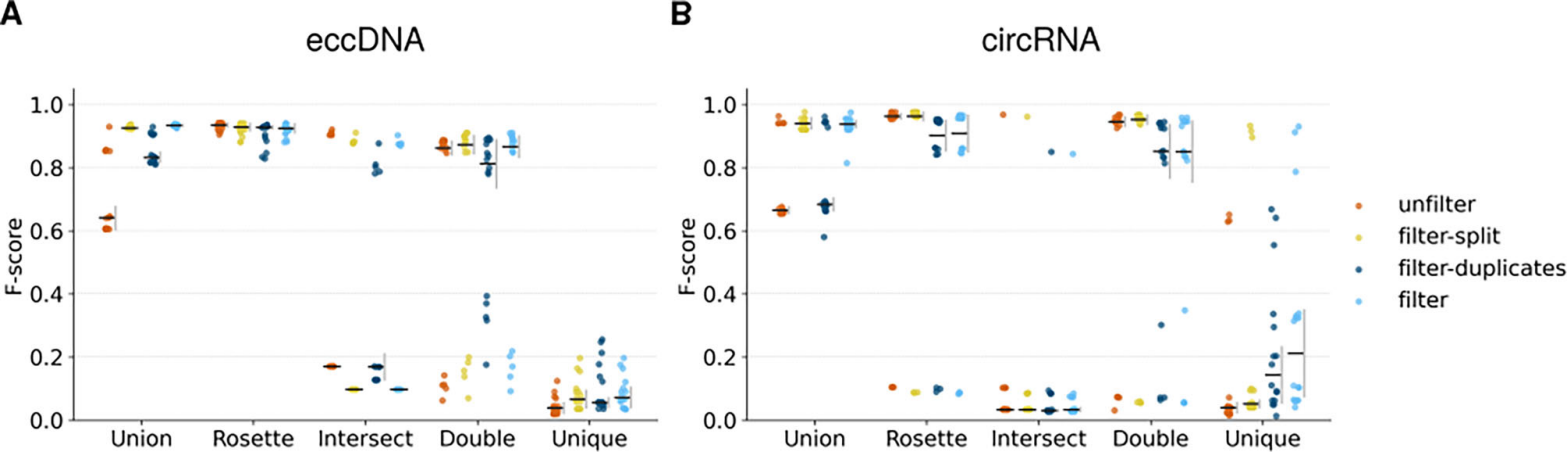
Performance analysis of software combinations for eccDNA and circRNA identification in *in in silico* datasets. Strip plot of the *F*-score of software combination strategies—*Union, Rosette, Intersect, Double*, and *Unique*—were evaluated under 4 filtering conditions: *unfilter, filter-split, filter-duplicates*, and *filter. F*-score values are shown for (A) eccDNA and (B) circRNA. For each combination-filter pair, the horizontal bar represents the mean value, and the vertical gray bar represents the standard deviation.

Two of the worst combination strategies were *Intersect* and *Unique*, both in eccDNA (Supplementary Materials 8–11) and circRNA (Supplementary Materials 13–16) (Fig. 6). The low *F*-scores were explained by a low precision and recall in *Unique*, and by a low recall but high precision in *Intersect. Unique* circles show low precision and recall values, indicating that simulated (true) circles are not likely to be detected by one tool (low recall) and, also, a *Unique* circle is likely not to be a true circle (low precision).

Nonetheless, for some tool combinations—the best performing ones—*Intersect* yields high precision values. In fact, we see a bimodal distribution for recall, indicating that some tool combinations detect the simulated circles more accurately; although this detection is thus highly dependent on the combination of tools. This effect will be discussed later for biological data.

Regarding *Union* strategy, where all circles detected by all tools are included, it showed high *F*-scores ($>$0.8 for most combinations), although their precision values were low, especially for *unfilter* circles. This effect may be driven by the inclusion of *Unique* circles, which already are linked to low *F*-scores. However, this showcases that a proper filtering may greatly improve the detection quality.

As expected, applying the filtering step led to a slight decrease in recall, which is attributable to an increase in FN, and improved precision values, specially for *Union* and *Unique* methods. However, this effect was less pronounced for *filter-duplicates* than for *filter-split*. Thus, *F*-scores were overall higher in *filter-split* than *filter-duplicates*; and sometimes were similar to *unfilter* or *filter* depending on the combination strategy.

Lastly, the 2 main strategies to discuss are *Rosette* and *Double*. Both strategies offer a balance between precision and recall. Focusing on eccDNA, *Rosette* yielded higher *F*-scores than *Double* after *filter-split* (2-tailed Dunn’s test, *p* = 0.016) as a result of a lower, although not significant, recall (*p* = 0.253). This difference was visible for the rest of filtering strategies, where *Rosette* showed a similar if not higher recall (Supplementary Material 12). For circRNA, *Rosette* and *Double* showed similar results. In fact, both *F*-scores, precision, and recall values were non significant (*p* = 1 for all) (Supplementary Material 17).

Overall, these findings highlight that the combination of 3 or more tools applied by *Rosette* improves detection accuracy for both eccDNA and circRNA. The choice between methods depends on whether maximizing precision, recall, or if a balanced performance is the priority. Additionally, filter option may affect the quality of the detection, with *filter* and *filter-split* showing better results than the rest of filtering options.

### Circle detection evaluation in biological data

Analysis of eccDNA and circRNA in biological data was performed with datasets with different processing techniques. For eccDNA, datasets generated using Circle-Seq and ATAC-seq were used; and for circRNA a dataset without RNase enrichment—RNase(−)—and with RNase enrichment—RNase(+)—were used. Each method has specific particularities affecting the quantity and quality of circle detection that will be further discussed.

#### Combined tool usage and *filter-split* improved eccDNA and circRNA detection

To better understand the detection dynamics among circular detection tools, filtering strategies, and circle enrichment techniques, Supplementary Figs S5–S8 show, for each method, the number of circles detected by each combination of tools (Supplementary Material 18).

In eccDNA, we observed higher detection rates using ATAC-seq compared to Circle-Seq. For instance, for the *unfilter* case, ATAC-seq detected 67,930 circles (5528.60 circles per million of reads), whereas Circle-Seq detected 15,498 circles (1208.04 circles per million of reads). However, Circle-Seq exhibited a higher proportion of circles retained by multiple tools—28.0% (689/1,474) retained when filtered for detection by 3 or more tools, compared to 8.94% (2,032/22,713) in ATAC-seq. Although the total amount remains higher in ATAC-seq, Circle-Seq proportionally showed more inner consistence in circle detection.

Furthermore, the number of circles detected by 4 out of 5 tools is extremely low in ATAC-seq (3/67,390 = 0.004% unfiltered circles), while Circle-Seq had a significantly higher ratio (642/15,489 = 4.14%) ($\chi ^2$ test with Yates correction, $\chi ^2$ = 2790.67, *p*  $<$ 0.0001). These observations suggest that Circle-seq captures circles with more robust detection across tools, which potentially indicates a higher-quality capture.

Regarding filtering strategies, both for ATAC-seq and Circle-Seq, *filter-split* retained fewer circles compared to *filter-duplicates* (36.7% vs. 74.2% for ATAC-seq and 23.02% vs. 31.6% for Circle-Seq). Nevertheless, the proportion of circles detected by 3 or more tools is considerably higher using *filter-split* (12.9% vs. 3.07% in ATAC-seq and 32.0% vs. 5.62% in Circle-Seq). Assuming that circles detected by multiple tools represent higher confidence detections, these results suggest that *filter-split* selects circles of higher confidence compared to *filter-duplicates*.

In terms of tool-specific performance for ATAC-seq, we identified tool groups with high joint detection (Supplementary Fig. S5): Circle_finder combined with CIRCexplorer2, Circle-Map individually, and ecc_finder-minimap2 combined with ecc_finder-bwa. These groups persist regardless of the filtering strategy, although circles detected exclusively by Circle-Map decrease when applying *filter-duplicates* and *filter*. In Circle-Seq, the groups ecc_finder-minimap2 combined with ecc_finder-bwa, Circle_finder combined with CIRCexplorer2, and CIRCexplorer2 individually appear (Supplementary Fig. S6). However, only ecc_finder-minimap2 combined with ecc_finder-bwa remains robust across different filters. This indicates clear similarities in circle detection among specific tool groups.

Similar dynamics were observed in circRNA detection. Initially, RNase(+) reported a higher number of detected circles compared to RNase(−). For the *unfilter* case, in RNase(+) 51,909 circles were detected (2208.42 circles per million of reads), whereas in RNase(−) 15,653 circles were detected (438.64 circles per million of reads). However, a lower proportion of circles subsequently passed the filters (e.g., *filter-split*: 11,139 (21.4%) for RNase(+) vs. 6,901 (44.1%) for RNase(−)). This suggests that RNase enrichment might increase sensitivity, requiring a more stringent filtering.

Furthermore, the filtering effect observed for circRNA mirrors that observed for eccDNA, with *filter-split* yielding a slightly higher percentage of consensus circles compared to *filter-duplicates* (RNase(+): 35.6% (3,969/11,139) vs. 22.8% (7,327/32,110); RNase(−): 30.5% (2,106/6,901) vs. 25.3% (2,392/9,469)). This reaffirms that, when choosing one strategy, *filter-split* is the superior filtering strategy. However, applying both filtering strategies yields a similar net number of circles (RNase(+) 5,538/51,909 (8.1%) vs. RNase(−): 4,620/15,653 (25.5%)), indicating that a 2-filtering system, if possible, may stabilize the number of circles.

#### $\Delta$ CJ is a proxy measure of circle detection quality

One of the limitations of biological data in this context is that no ground truth is available, and thus, alternative metrics must be employed to assess detection performance. In this study, we propose the difference in read coverage across each nucleotide of the CJ ($\Delta$CJ) as a proxy for precision. The rationale behind this metric is that, assuming that reads mapped to the CJ are detected on the left side of the CJ at the same rate as on the right side of the CJ, any imbalance in the detection (e.g., a circle with 2 reads assigned on the left side and 28 on the right side) is likely to be a result of an incorrect circle detection; for which a better-suited circle may or may not be available. To further improve the robustness of this metric, $\Delta$CJ was refined to account for sequence mappability and read-level alignment quality (described in the “Materials and methods” section).

To illustrate the rationale and behavior of the refined metric, in Supplementary Fig. S9, we depict 3 cases based on *in silico* data where each circle was incorrectly detected along with its corresponding TP circle. Circle A represents a case of an exact duplication, where both circles share identical coordinates, resulting in equal read support on both sides of the junction and consequently identical $\Delta$CJ = 0 and $p_{\mathrm{adj}}$ = 1. Circles B and C, in contrast, illustrate partial duplications, where one end of the detected circle does not match the true simulated coordinates. In circle B, the undetected right-side junction region exhibits a lower mappability score (0.470) compared to the mapped region (1), which explains the complete lack of reads on the right side. In circle C, both sides of the junction have high mappability (1.0), but the alignment quality of reads supporting the left side is substantially lower (42.793) compared to the right side (60), leading to a reduced effective read count after MAPQ weighting. Thus, these examples illustrate how the $\Delta$CJ$_{\mathrm{adj}}$ distinguishes true from false circles (Table 3).

**Table 3:** $\Delta$ CJ and $p_{\mathrm{adj}}$  $\Delta$CJ values of 3 circle detection examples.

Circle	Condition	Reads (L|R)	$\Delta$ CJ	$p_{\mathrm{adj}}$ $\Delta$CJ
chr6:39,754,059–39,754,823	TP	22 $|$ 22	0	1
chr6:39,754,063–39,754,823	FP	22 $|$ 22	0	1
chr3:140,171,091–140,172,928	TP	28 $|$ 27	0.029	0.110
chr3:140,171,091–140,174,822	FP	28 $|$ 0	1	$1.3 \times 10^{-6}$
chr17:6,795,106–6,797,132	TP	25 $|$ 29	0.072	1
chr17:6,794,297–6,797,033	FP	6 $|$ 39	0.734	$2.7 \times 10^{-6}$

Assuming, for each circle with $k_L$ reads assigned to the left, and $k_R$ reads assigned to the right, we can compute the probability *p* of this configuration, assuming a binomial $B(n=k_L+k_R, p=0.5)$ distribution. Thus, we can set a circle with a high imbalance (0 or 1 read assigned on one side) as an incorrect circle with $\alpha =0.05$ if it contains 9 or more reads (described in the “Materials and methods” section). Based on this, we will compute a series of metrics: (1) proportion of circles with $\ge$9 reads, (2) mean and median $\Delta$CJ, and (3) ratio of circles with $p_{\mathrm{adj}} < 0.05$, where probabilities are adjusted by the Benjamini–Hoechberg method.

To ensure that the metric is a correct proxy for the accuracy of circle detection, we applied it to *in silico* data. To that end, in Supplementary Figs S10A and B, we show the $\Delta$CJ distribution of TP reads and FP reads for eccDNA and circRNA, respectively. Overall, simulated circles follow a right-tailed distribution of $\Delta$CJ, with median values smaller than 0.1 for both eccDNA and circRNA, showing that most circles have a balanced left/right read distribution. The $\Delta$CJ distributions arising after filtering showed no statistically significant differences, neither for eccDNA (Kruskal–Wallis [KW] test, *H* = 8.78, $p_{\mathrm{adj}}$ = 0.067) nor for circRNA (*H* = 0.01, $p_{\mathrm{adj}}$ = 0.999).

In eccDNA, the unfiltered FP circles showed a wider $\Delta$CJ distribution, with a mean value of 0.367, and many of them with $\Delta$CJ = 1 (75th percentile is 1). Similarly, circles that still remain FP after filtering showed a high median $\Delta$CJ (*filter*, 0.810), even higher than the unfiltered circles. To better understand the individual filtering effect, circles remaining FP after *filter-split* showed a lower $\Delta$CJ than circles remaining after *filter-duplicates*. This may indicate that *filter-split* removes high-$\Delta$CJ circles, whereas *filter-duplicates* removes low-$\Delta$CJ circles. This effect is also observable based on their assignment probability: there is a higher proportion of circles retained after *filter-duplicates* that have $p_{\mathrm{adj}}$  $<$ 0.05 (94%), indicating that most of these are unbalanced, in contrast to the proportion of circles retained after *filter-split* (26%) (Supplementary Material 19). Thus, most of these low-$\Delta$CJ circles are, expectedly, duplications of simulated circles, and thus may contain a balanced left/right-assigned reads ratio. On the other hand, high-$\Delta$CJ circles may represent new circles, which escape duplicate-filtering, and which contain an imbalanced left-right read ratio, as explained before. These circles are likely to arise as an artifact during the alignment step, where reads are assigned incorrectly to other genomic areas on the side with the lowest read presence.

Interestingly, the results on circRNA are different. All circles retained as FP after filtering remain with a low $\Delta$CJ; with the exception of *filter*, which has a similar $\Delta$CJ distribution as in eccDNA. These distributions are likely because most of these circles may be assigned uniquely by segemehl, which contains an extremely high amount of duplicated circles, most of which may be even remain after *filter-duplicates*. Therefore, the remaining circles contain a low $\Delta$CJ. Interestingly, the ratio of FP circles with $p_{\mathrm{adj}}$  $<$ 0.05 is higher after filter-split (44%) than after *filter-duplicates* (12%) indicating that many of the removed circles by *filter-duplicates* probably had a biased left/right read ratio (Supplementary Material 20).

#### Regional mappability and read mapping quality influences the detectability of circular molecules

Given that read alignment accuracy depends on regional mappability, we examined the extent to which low-mappability regions and MAPQ contribute to the non-detection or incorrect of circular molecules. Beyond the general effect of mappability on detectability, a detailed inspection of TP, FP, and FN shows that these categories differ more from one another than across tools (Supplementary Figs S13 and S14).

Focusing first on eccDNA, TP events consistently occur in regions of high mappability and are supported by reads with high MAPQ, reflecting confident and unambiguous alignment. In contrast, there is a considerable proportion of FN (undetectable circles) containing at least 1 CJ segment in a low-mappability region, which results in poorly aligned or completely absent reads, as evidenced by their minimal MAPQ values. Among the 1,000 simulated eccDNAs, 78 were not detected by any tool; of these, 61 contained at least 1 region lacking a mappability value, and 58 had no mapped reads. Similarly, in circRNA, 11 circles were undetectable, 7 of which showed at least 1 region without a mappability value, and 6 had no mapped reads.

FP predictions, although arising from regions with slightly lower mappability than TP, are most strongly characterized by reads with very low MAPQ. Thus, observing low MAPQ as a shared component of FP and FN circles suggests that alignment uncertainty rather than regional mappability itself is the primary driver of false detections.

Of note, mappability and MAPQ distributions show a clear bimodal distribution in the violin plots of Supplementary Figs S13 and S14, which flattens into a unimodal distribution with a lower tail for distributions with increased circle number. This bimodal distribution may indicate that FN and FP detection is governed by 2 factors: on the one hand, circles with a decreased mappability and/or MAPQ, which causally affect read alignment and therefore also affect circle detection; on the other hand, circles with high mappability and MAPQ values, which are nonetheless incorrectly detected. Since the proportion of these circles is tool-dependent and increases with the number of falsely detected circles, this set of circles may be affected by tool-dependent factors, and thus is not influenced by MAPQ or mappability.

Differences between tools are comparatively small: for instance, CIRCexplorer2 in eccDNA generates FP supported by extremely low-MAPQ reads, whereas ecc_finder-minimap2 shows minimal sensitivity to regional mappability. Overall, detection accuracy is governed more by the intrinsic properties of the circles (TP, FP, and FN) than by tool-specific behavior, underscoring the central roles of alignment quality and genomic mappability in both eccDNA and circRNA detection (Supplementary Figs S11 and S12).

Focusing on circRNAs, we observe that the overall distributions of mappability and MAPQ values both for FP and FN circles are more biased toward values of 1, which indicates a much lower proportion of falsely detected circles explained by low mappability and/or MAPQ. This showcases that the differences in the biogenesis between circRNAs and eccDNAs are translated into tool-specific detection effect. In fact, the increased tendency of FP and FN in circRNAs compared to eccDNA may indicate that circle detection tools may be hindered by the more complex biological properties of the former circles.

#### Circle capture method greatly affected detection quality in biological data

**Figure 7:**
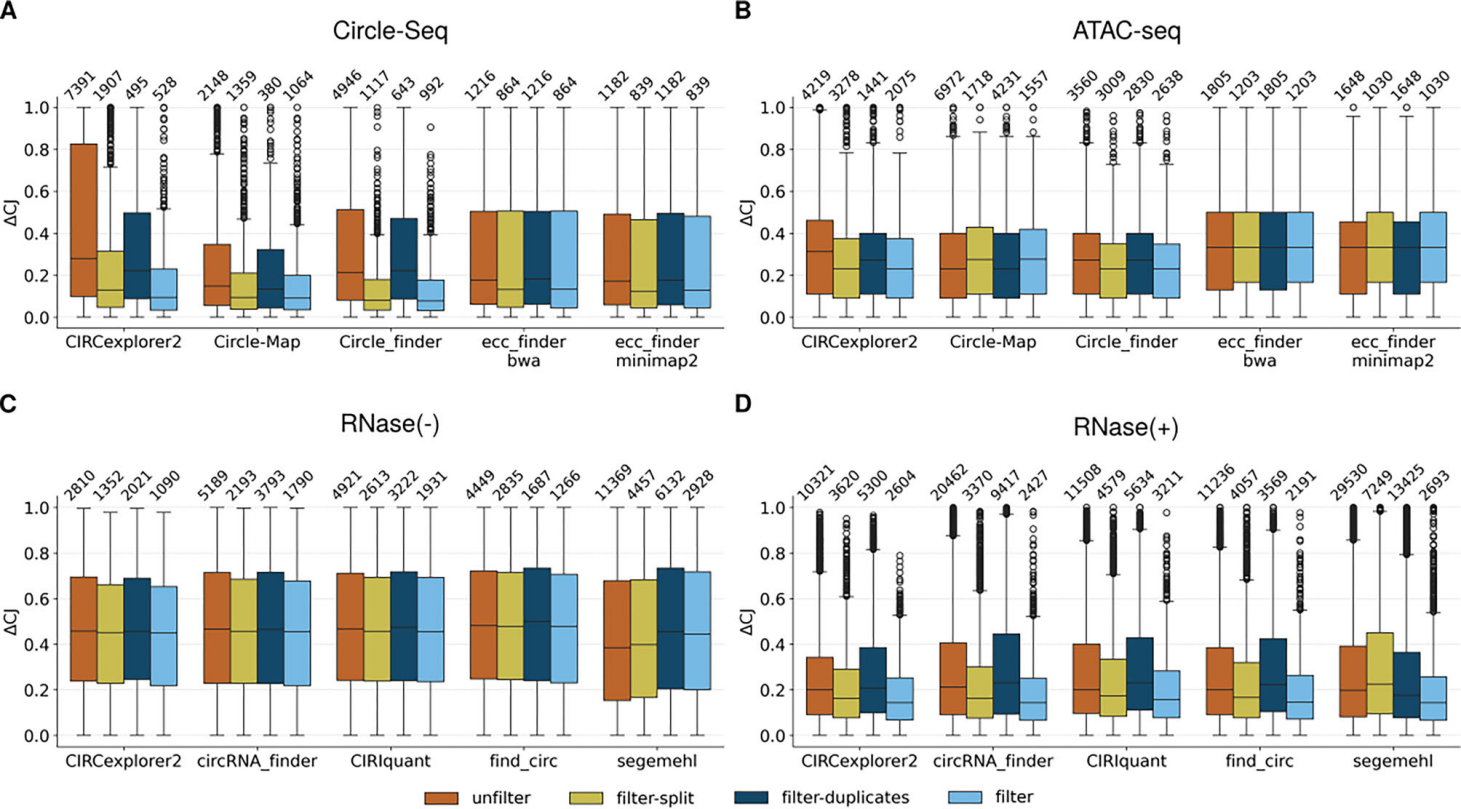
Performance analysis of detection software for eccDNA and circRNA identification in biological datasets. Boxplots of $\Delta$CJ values for (A) Circle-Seq and (B) ATAC-seq data for eccDNA, and (C) RNase(−) and (D) RNase(+) data for circRNA, under 4 filtering conditions: *unfilter, filter-split, filter-duplicates*, and *filter*.

After establishing $\Delta$CJ as a proxy measure to evaluate the fit of the circular detection, we wanted to observe the variations in $\Delta$CJ for the different circle capture methods in biological data. Figure 7 shows $\Delta$CJ values for all 4 circle capture methods in each circle detection tool and the 4 filtering methods.

Regarding eccDNA detection tools, similarities between individual tools are visible again. For instance, ecc_finder-bwa and ecc_finder-minimap2 showed a similar detection pattern based on the $\Delta$CJ for both Circle-Seq and ATAC-seq, and showed overall uniformly increased $\Delta$CJ values at 0.3 (range 0.272–0.333) in ATAC-seq. In Circle-Seq, the filtering effect on the $\Delta$CJ is more apparent than for ATAC-seq. Generally, there are more circles and with lower $\Delta$CJ values remaining after *filter-split*, compared to *filter-duplicates*. $\Delta$CJ values for *filter* are similar, if not lower, to *filter-split*, suggesting again that these 2 filtering strategies are optimal also for biological datasets.

Additionally, ATAC-seq has a tendency to capture more circles than Circle-Seq, as mentioned before. Interestingly, in tools and filter combinations where the circle number is similar or higher, $\Delta$CJ are higher in ATAC-seq; with few exceptions like *filter-duplicates* in Circle_finder and CIRCexplorer2. Also, $\Delta$CJ for ecc_finder tools are markedly higher in ATAC-seq compared to Circle-Seq. Therefore, it is likely, as mentioned in the previous section, that the higher number of circles captured by ATAC-seq may show a negative correlation with circle quality.

Focusing on circRNA, there is a clear difference in the distribution of $\Delta$CJs: RNase(−) has a quite stable $\Delta$CJ at around 0.4 (differences in *filter*, KW test: *H* = 10.46, *p* = 0.033); mostly driven by segemehl; while effects across filters are more pronounced in RNase(+) (differences in *filter*, KW test: *H* = 45.758, *p* = 2.8$\times 10^{-9}$), which shows a clearly reduced $\Delta$CJ compared to RNase(−). In this case, too, compared with eccDNA, a lower number of detected circles correlates with the $\Delta$CJ value (e.g., with different filters) indicating that the 2-filtering process tends to retain circles with lower $\Delta$CJ. Additionally, and similarly to eccDNA, circles remaining after *filter-split* show lower $\Delta$CJs than after *filter-duplicates*. Therefore, these results suggest that RNase treatment enhances detection accuracy (Supplementary Materials 21 and 22).

#### *Rosette* tool combination yields the best trade-off between detection accuracy and number of circles in biological data

**Figure 8:**
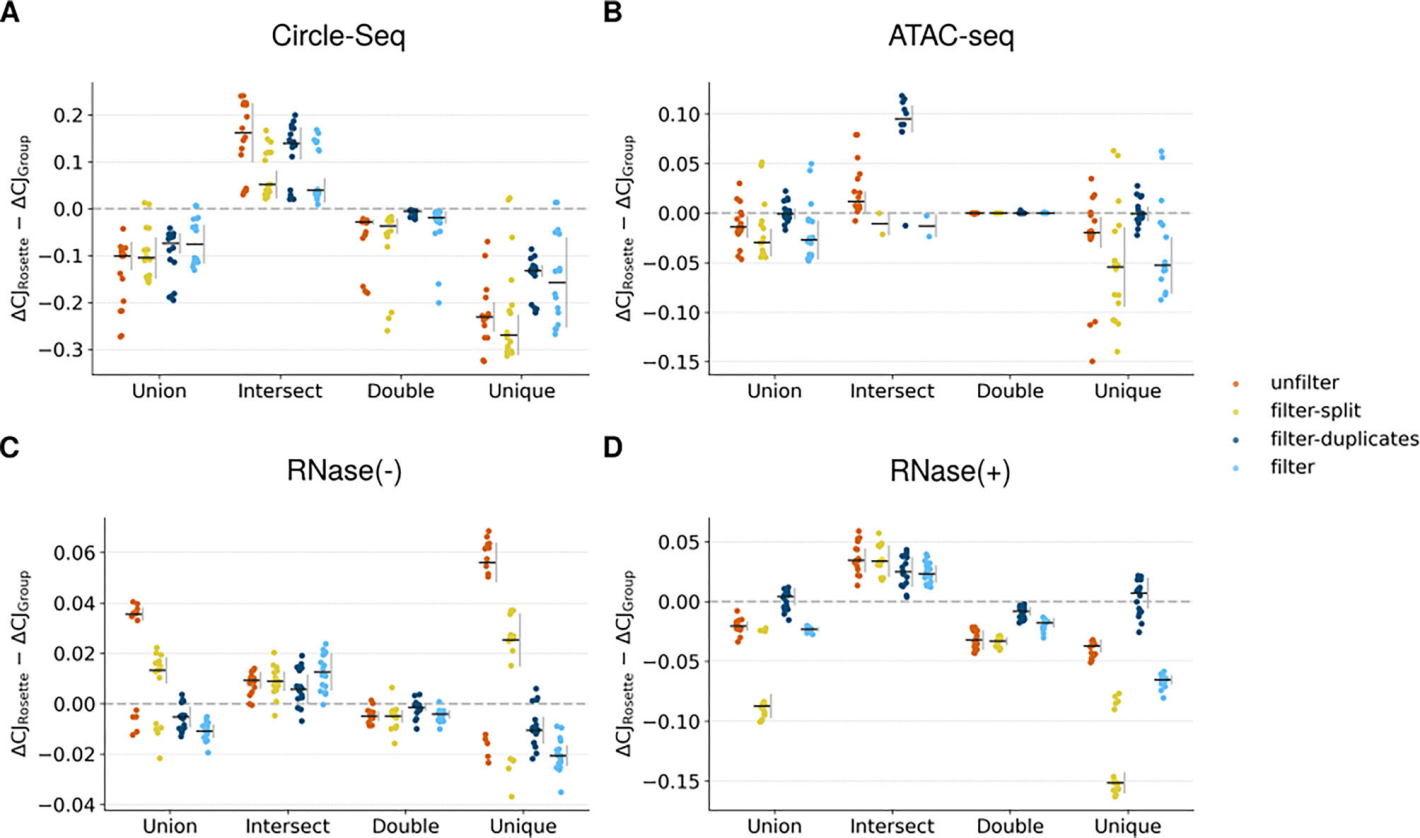
Performance analysis of software combinations for eccDNA and circRNA identification in biological data. Strip plot of $\Delta \Delta$CJ values ($\Delta$CJ$_{\mathrm{Rosette}}$ − $\Delta$CJ$_{\mathrm{Group}}$) for different tool combinations—*Union, Rosette, Intersect, Double*, and *Unique*—evaluated on biological data, with comparisons made against the *Rosette* combination. Results are shown for (A) Circle-Seq and (B) ATAC-seq data for eccDNA, and (C) RNase(−) and (D) RNase(+) data for circRNA, under 4 filtering conditions: *unfilter, filter-split, filter-duplicates*, and *filter*.

The final aspect of this analysis is centered on evaluating tool combinations. Firstly, we assessed how $\Delta$CJ varies depending on the combination of tools. To this end, Supplementary Fig. S13 shows the $\Delta$CJ values for each combination (Supplementary Material 23), while Fig. 8 illustrates the difference in $\Delta$CJ between the *Rosette* combination and all other combinations ($\Delta \Delta$CJ).

From this analysis, although results vary considerably based on the filter and circle enrichment method, we observed a general trend: the rest of tool combination strategies consistently yielded $\Delta \Delta$CJ $<$ 0, meaning that the *Rosette* combination typically has a lower $\Delta$CJ compared to other combinations. This trend is particularly evident when comparing *Rosette* with *Unique*, where $\Delta \Delta$CJ is the most pronounced. Regarding filtering, as previously discussed, the lowest $\Delta$CJ values are generally achieved with the *filter* strategy, followed by *filter-split*.

The only exception exhibiting $\Delta \Delta$CJ $>$ 0 occurred with the *Intersect* combination. This result is expected, as *Intersect* exclusively selects circles detected consistently by all tools. However, *Intersect* detected about half of the circles compared to *Rosette* (Supplementary Fig. S14), suggesting that the *Rosette* combination might provide a better balance between the number of detected circles and detection quality.

The $\Delta$CJ values observed between *Rosette* and *Double* combinations were quite similar, with *Rosette* generally having slightly lower $\Delta$CJ values. Nevertheless, this relationship depends on the applied filter (e.g., in RNase(+) $\Delta \Delta$CJ varies from −0.002 to −0.043). This similarity in $\Delta$CJ between *Double* and *Rosette* is to some extent surprising, considering that *Double* excludes *Intersect* circles. This showcases that the weight of *Rosette*  $\Delta$CJ comes from double circles instead of *Intersect* ones.

Finally, we investigated how the number of tools affects $\Delta$CJ. In Supplementary Fig. S15, $\Delta$CJ is plotted against the number of tools in each tool combination, circle enrichment technique, and filter. Generally, although particularly for *Rosette*, a higher number of tools slightly increased $\Delta$CJ. In certain cases (especially for *Double*), combinations involving more tools produce $\Delta$CJ values midway between individual combinations.

Our interpretation of this phenomenon is that adding more tools could introduce biases from tools such as segemehl or ecc_finder, which, as previously observed, may have lower detection accuracy. Thus, $\Delta$CJ values are higher, thereby slightly increasing the combined circles’ $\Delta$CJ. However, this increase in $\Delta$CJ from including additional tools is considerably smaller than the effect introduced by the filtering method or circle enrichment technique.

We observed the opposite effect with *Intersect*, where the addition of more tools skewed the $\Delta$CJ distribution toward lower values. As previously explained, this occurs because *Intersect* retains only circles consistently detected across multiple tools, inherently selecting those with greater detection reliability and thus lower $\Delta$CJ, at a cost of a lowered number of circles.

## Discussion

The accurate identification of eccDNA and circRNA is essential for understanding their biogenesis, functions, and implications in diseases. These circular molecules play significant roles in gene regulation and are increasingly recognized for their involvement in various pathological conditions, including cancer and neurodegenerative disorders, highlighting their potential use in disease diagnosis and monitoring [9–11, 52]. However, the lack of standardized evaluation frameworks and consensus protocols for their detection poses a significant challenge, often leading to inconsistencies across studies. To address this issue, we conducted a systematic benchmarking of existing detection methods using both *in silico* and biological datasets, aiming to clarify their strengths, limitations, and practical applicability.

Detection performance varied significantly depending on the type of circular molecule analyzed. Regarding eccDNA, we observed that ecc_finder-minimap2 and CIRCexplorer showed robust results, followed by Circle-Map and Circle_finder. However, applying any filtering strategy led to similar outcomes across all tools. These observations are consistent with findings reported by Li et al. [21] and Gao et al. [20], who also identified Circle-Map and Circle_finder as top-performing tools. Furthermore, Gao et al. [20] reported poor performance of ecc_finder-bwa, aligning with our results and indicating partial reproducibility between studies.

In the case of circRNAs, the comprehensive analysis by Zeng et al. [22], although not entirely replicable in our study, suggests a correlation between the number of circles detected and the *F*1-score for each tool. Conversely, Vromman et al. [23] observed a clear detection bias, finding segemehl among the tools with lower detection rates, contrasting sharply with our results. This discrepancy could arise from several factors, including the fact that segemehl, initially designed as an aligner, was later integrated into circRNA analysis workflows; consequently, its performance might be highly dataset-dependent. Additionally, Liu et al. [53] analyzed the effect of coverage similar to our study, although they did not observe significant impacts of coverage on detection, possibly due to their analysis not including higher coverage levels like ours, where we indeed observed an increase in FPs. They also reported better performance for CIRI and circRNA_finder compared to our findings, potentially explained by their use of CIRI-simulator [49] for circle simulation, which may inherently bias results in favor of these tools.

Focusing on biological datasets, we found the library preparation method to be one of the most influential factors for both eccDNA and circRNA detection (ATAC-seq vs. Circle-seq for eccDNA, and RNase+ vs. RNase− for circRNA). For eccDNA, we detected a greater number of circles using ATAC-seq compared to Circle-seq, conflicting with results reported by Gao et al. [20], who observed significantly higher detection rates (up to 2 orders of magnitude) for Circle-seq. This discrepancy might be attributed to sample origin or other internal methodological factors, though the exact reason remains unclear.

In contrast, our circRNA findings align well with studies such as Zeng et al. [22], who reported increased circle detection following RNase treatment (~1.5–3 times higher). Nonetheless, we observed that circle detection numbers strongly depend on the tools employed. One potential confounding factor influencing this variability is the alignment algorithm, which differs among tools. To minimize this variability, we standardized the aligners used as much as possible—employing STAR for circRNA and BWA for eccDNA detection. However, disparities in results still indicated significant aligner-specific factors influencing circle detection accuracy.

We also conclude that targeted amplification methods notably affect detection outcomes, potentially introducing additional biases. For example, Rolling Circle Amplification, used in Circle-seq, can lead to either overrepresentation or underrepresentation of circles [54]. Moreover, such amplification methods may impede the analysis of epigenetic signals, which are linked to gene expression variations and regulatory alterations. Consequently, non-targeted sequencing approaches such as ATAC-seq and RNA-seq are gaining interest, although further investigation is necessary to fully understand and address potential biases inherent in these methods. Additionally, the analysis derived from our results suggests a preference of use of targeted methods such as Circle-seq compared to untargated methods like ATAC-seq.

Delving deeper into the analysis, several structural effects deserve discussion, particularly the issue of FPs. Initially, we observed that coverage substantially influences FP detection in *in silico* data, a result whose applicability to biological data remains unclear but warrants consideration in analyses. In fact, such decrease in precision at high coverage may appear driven by accumulation of low-confidence split reads, and tool-specific sensitivity to noise. Therefore, high coverage may not per se be intrinsically detrimental, but rather that current algorithms lack sufficient FP control at high depth without stricter filtering.

We aimed to understand the origin of these FPs. Zeng et al. [22] defined FPs as circles detected in RNase(−) treatment but not in RNase(+), which theoretically amplifies the circRNA signal. Our findings highlight the need for further exploration of FP origins, particularly in *in silico* datasets, to enhance their applicability in biological studies. We propose that FPs may originate from 2 main sources: computational and biological effects. Computational effects include coverage-related issues, where increased sequence detection near the CJ may occasionally lead to incorrect identification due to partial flanking or sequencing errors, creating artificial circles. Additional computational factors encompass sequence length, the aligner used, and the detection algorithm itself. Biological effects primarily involve amplification methods—techniques excluding linear DNA or RNA can reduce FPs by preventing amplification of sequences resembling CJ, which may result from alternative splicing or genomic mutations and rearrangements that do not yield genuine circles. Consequently, methods involving purification steps (such as Circle-Seq for eccDNA and RNase(+) for circRNA) generally provide superior results compared to those lacking this step.

Another crucial factor affecting the analysis and significantly reducing FPs is the appropriate use of filters. Both *in silico* and biological datasets demonstrated a considerable decrease in detected circles upon filtering. Our findings align with Vromman et al. [23], who reported substantial reduction in circles with $\ge$5 reads mapped to the BSJ.

In our analysis, apart from applying a filter based on counts ($\ge$2 reads), we also implemented a duplicate filter. Generally, both filters performed adequately, though the filter-split occasionally yielded slightly superior results. A potential reason is that many tools do not provide precise CJ coordinates; rather, they often detect circles at adjacent coordinates, typically supported by low read counts (1 or 2) and occasionally mismatches that cause partial misalignment. Thus, many spurious circles escape duplicate filtering, making filter-split more effective in such cases.

An additional intriguing observation from our *in silico* analysis is related to circle length. We noted a marked decline in detected circles around 340 bp in both eccDNA and circRNA, possibly corresponding to dinucleosome length. However, since *in silico* data are independent of biological factors, this finding is surprising. It could be attributed to detection biases such as those observed in ecc_finder, which fails to detect shorter eccDNAs ($<$400 bp), an issue also reported by Li et al. [21]. Nonetheless, this issue would suggest that circle length biases associated with tool usage require further study.

Lastly, repetitive sequences significantly impact circle detection. Gao et al. [20] observed pronounced effects on detecting reads associated with LTR, SINE, and LINE elements, depending on whether the sequencing technology involved long or short reads, albeit representing a small fraction of total reads (0.1% for short reads). Our *in silico* results showed *F*-scores slightly above 0.8 for eccDNA and between 0.8 and 1 for circRNA for LINEs and SINEs, suggesting a slightly higher yet potentially problematic detection fraction in biological datasets due to the repetitive nature of these sequences. The most pronounced effect occurred with satellite sequences, where detection accuracy declined sharply. This phenomenon may substantially impact eccDNA detection in centromeric regions, which, due to their repetitive nature, could be inaccurately captured, potentially leading to significant functional loss in analyses. This effect is related to the lack of circle detection or lowered detection accuracy in low mappability regions, which has been shown in our analysis.

A significant limitation of current benchmarking studies is the absence of robust metrics for evaluating accurate circle detection in biological data. Often, comparisons rely on the number of detected circles or similarity between tools, neither of which provides true validation. Alternative methods such as external validations using qPCR or similar techniques cannot scale effectively beyond a few circles.

In this study, we employed the $\Delta$CJ metric, developed under the assumption that reads assigned to the CJ will equally distribute between its left and right sides. Consequently, significant deviations from this symmetry indicate potential incorrect assignments. After confirming the rationale through specific examples and verifying its applicability in *in silico* data, we found that the use of $\Delta$CJ in biological datasets provides valuable insights for evaluating tool effectiveness, purification methods, and filtering strategies.

Additionally, although the results are only suggestive and would require a more in-depth study, the use of $\Delta$CJ has uncovered a new possible mechanism for the generation of FP and FN circles, dependent on the mappability of the genome and the mapping quality. Mapping quality is generated during the read alignment process and represents the probability of a read being correctly assigned to that specific region. Reads with low read quality may arise from sequencing artifacts or from mapping to a somewhat similar sequence to the read, which may correspond to a secondary region that was propritized during the alignment. Thus, part of FP circles are mistakenly assigned to a region different from the CJ, leading to a low mapping quality alignment. On the other hand, FN circles may arise from a 2-fold combination of aligning reads to low mappability regions, which produce low-quality alignments due to the increased mismatch rate, and therefore are not accurately detected by the circle detection tools. Thus, circular molecules arising from low-mappability regions have a higher probability of being underdetected under short-read sequencing strategies. Future studies comparing mappability distributions of circles detected under short-read and long-read strategies would expand the insights of the effect of this metric on circle detection.

Therefore, the inclusion of $\Delta$CJ as a surrogate metric for circle detection quality, as well as mapping quality and region mappability metrics to confirm circle detection errors, may be beneficial for studies to improve the quality of reported circles, or to evaluate detection biases, where the higher-than-expected FPs are likely to be mitigated by this metric.

Despite its relevance, it is crucial to recognize the conceptual and practical limitations of $\Delta$CJ. For instance, some FP circles might escape detection due to factors unrelated to symmetry imbalances, limiting the metric to detecting only certain types of FPs. Additionally, the reliability of this metric requires a relatively high number of reads (preferably more than 20–30), restricting its effectiveness for detecting circles with low read counts. One advantage of analyzing read unbalances is that high-read FPs with significant asymmetry may potentially be reassigned to circles sharing one CJ coordinate, generating thus a corrected circle list. Lastly, we observed that for circRNAs, the metric is noisier, possibly because many circRNAs have boundaries defined precisely at intron–exon junctions, conflicting with the expected randomness of read distribution. To improve on these limitations, a more robust theoretical framework of circle read mapping to CJ that accounts for sequencing depth and circle length may be necessary.

One advantage of incorporating multiple detection tools in analyses is the improvement in eccDNA and circRNA detection, especially considering analysis workflows like those implemented in nf-core, which facilitate integration of multiple tools [28]. This observation aligns with Hansen [26], who identified commonly detected circles as *bona fide* circles. Other studies have similarly highlighted the variability in detection consistency among tools. For instance, Vromman et al. [23] reported that nearly 50% of circles detected were unique to a single tool—though the exact percentages varied considerably by tool—while ~10% were consistently detected across 10 or more tools. Likewise, Li et al. [21] suggest that including 2 or more tools can enhance the robustness of circle detection.

Vromman et al. [23] validated circles consistent across multiple tools, observing that employing 2 or more tools notably reduced FPs. However, while this outcome could theoretically extend to eccDNA, direct validation for eccDNA is lacking, and additional validation measures for various tool combinations are needed.

Despite the *bona fide* circle strategy being reported in previous works, our benchmark is the first suggesting a well-defined and demonstrated tool combination strategy, *Rosette*, that achieves the best balance between the number of circles reported and FP reduction.

The rationale for the *Rosette* strategy is that circles detected by multiple tools are likely to represent TP, whereas those identified by a single tool should be interpreted cautiously. Among all tested combinations, *Rosette* exhibited superior performance in simulated data by significantly lowering the FP rate while maintaining high sensitivity, even without additional filtering. Similarly, in biological data, it enhanced detection capacity without compromising accuracy. Nevertheless, further validation using deeper analysis of reads associated with detected circles would bolster the reliability of this approach.

Additionally, we observed that the effectiveness of combined detection strategies depends on the individual accuracy of each tool. This aligns with Vromman et al. [23], who found that the FP rate for combined tools approximates an average of the individual FP rates. Thus, the choice of combination strategy (particularly between *Rosette* and *Intersect*) depends on the analytical goal. In clinical settings or environments requiring higher positive predictive value, *Intersect* may be more advantageous despite detecting fewer circles. In contrast, *Rosette* may offer a sufficiently robust and reliable circle set suitable for broader research applications.

## Availability of source code and requirements

- Project name: benchmarking.
- Project homepage: https://github.com/ZabalaAitor/benchmarking.
- Notebook output repository: Zenodo [55].
- License: MIT License.
- SciCrunch RRID: SCR_027896.
- Operating system: Platform independent.
- Programming language: Python.

## Additional files

**Supplementary Material 1**. Performance analysis of detection software for eccDNA identification in *in silico* datasets.

**Supplementary Material 2**. Performance analysis of detection software for circRNA identification in *in silico* datasets.

**Supplementary Material 3**. Kolmogorov–Smirnov test for circular length distribution in *in silico* datasets.

**Supplementary Material 4**. Repeat element annotation for eccDNA in *in silico* datasets.

**Supplementary Material 5**. Repeat element annotation for circRNA in *in silico* datasets.

**Supplementary Material 6**. Genomic element annotation for eccDNA in *in silico* datasets.

**Supplementary Material 7**. Genomic element annotation for circRNA in *in silico* datasets.

**Supplementary Material 8**. Performance analysis of software combinations detection for eccDNA in *in silico unfilter* condition datasets.

**Supplementary Material 9**. Performance analysis of software combinations detection for eccDNA in *in silico filter-split* condition datasets.

**Supplementary Material 10**. Performance analysis of software combinations detection for eccDNA in *in silico filter-duplicates* condition datasets.

**Supplementary Material 11**. Performance analysis of software combinations detection for eccDNA in *in silico filter* condition datasets.

**Supplementary Material 12**. Dunn’s test results for software combination detection performance metrics for eccDNA in *in silico* datasets.

**Supplementary Material 13**. Performance analysis of software combinations detection for circRNA in *in silico unfilter* condition datasets.

**Supplementary Material 14**. Performance analysis of software combinations detection for circRNA in *in silico filter-split* condition datasets.

**Supplementary Material 15**. Performance analysis of software combinations detection for circRNA in *in silico filter-duplicates* condition datasets.

**Supplementary Material 16**. Performance analysis of software combinations detection for circRNA in *in silico filter* condition datasets.

**Supplementary Material 17**. Dunn’s test results for software combination detection performance metrics for circRNA in *in silico* datasets.

**Supplementary Material 18**. Circular detection in biological datasets.

**Supplementary Material 19**. $\Delta$CJ evaluation as a proxy measure of circle detection quality for eccDNA identification in *in silico* datasets.

**Supplementary Material 20**. $\Delta$CJ evaluation as a proxy measure of circle detection quality for circRNA identification in *in silico* datasets.

**Supplementary Material 21**. Performance analysis of detection software in biological datasets.

**Supplementary Material 22**. Dunn’s test results for software combination detection performance metrics in biological datasets.

**Supplementary Material 23**. Performance analysis of detection software combinations in biological datasets.

**Supplementary Figure S1**: Performance analysis of detection software for eccDNA and circRNA identification in *in silico* datasets.

**Supplementary Figure S2**: Circular length distribution analysis in *in silico* datasets.

**Supplementary Figure S3**: Repeat element analysis and genomic element analysis in *in silico* datasets.

**Supplementary Figure S4**. Performance analysis of software combinations for eccDNA and circRNA identification in *in silico* datasets.

**Supplementary Figure S5**: eccDNA detection in Circle-Seq data.

**Supplementary Figure S6**: eccDNA detection in ATAC-seq data.

**Supplementary Figure S7**: eccDNA detection in RNase(−) data.

**Supplementary Figure S8**: circRNA detection in RNase(+) data.

**Supplementary Figure S9**: IGV visualization of 3 eccDNA false positives in *in silico* datasets.

**Supplementary Figure S10**: Circular junction nucleotide difference ($\Delta$CJ) in *in silico* datasets.

**Supplementary Figure S11**: Circular junction mappability in *in silico* datasets.

**Supplementary Figure S12**: Circular junction read mapping quality (MAPQ) in *in silico* datasets.

**Supplementary Figure S13**: Circular junction nucleotide difference ($\Delta$CJ) of software combinations for eccDNA and circRNA identification in biological datasets.

**Supplementary Figure S14**: Comparation of detected circles between Rosette and Intersect.

**Supplementary Figure S15**: Performance of tool combinations for eccDNA and circRNA in biological datasets.

## Abbreviations

BSJ: backsplice junction; CJ: circular junctions; circRNA: circular RNA; $\Delta$CJ: the discrepancy in read assignment to each side of the breakpoint; DNASE1L3: deoxyribonuclease 1-like 3; eccDNA: extrachromosomal circular DNA; FN: false negative; FP: false positive; KS: Kolmogorov–Smirnov test; KW: Kruskal–Wallis test; LINE: Long Interspersed Nuclear Element; LTR: Long Terminal Repeats; MAPQ: mapping quality; NCBI: National Center for Biotechnology Information; pre-mRNA: precursor messenger RNA; SINE: Short Interspersed Nuclear Elements; snRNA: small nuclear RNA; TP: true positive; UTR: untranslated region; WGS: whole-genome sequencing; Ø: non-repetitive region.

## Supplementary Material

giag017_Supplemental_Files

giag017_Authors_Response_To_Reviewer_Comments_original_submission

giag017_GIGA-D-25-00520_original_submission

giag017_GIGA-D-25-00520_revision_1

giag017_Reviewer_1_Report_Original_submission Reviewer 1 -- 1/11/2026

giag017_Reviewer_2_Report_Original_submission Reviewer 2 -- 1/23/2026

giag017_Reviewer_2_Report_Revision_1 Reviewer 2 -- 2/2/2026

## Data Availability

The data sets supporting the results of this article are available in Zenodo [56] for processed data, and in the ENA repository, accession number PRJEB95764, for raw *in silico* generated data.
