# Supplementary figures and images for: Comparative analysis of eccDNA and circRNA tools shows increased accuracy of tool combination
Source: Gigascience. 2026 Feb 25;15:giag017. doi: 10.1093/gigascience/giag017 (PMC13154841; doi:10.1093/gigascience/giag017)

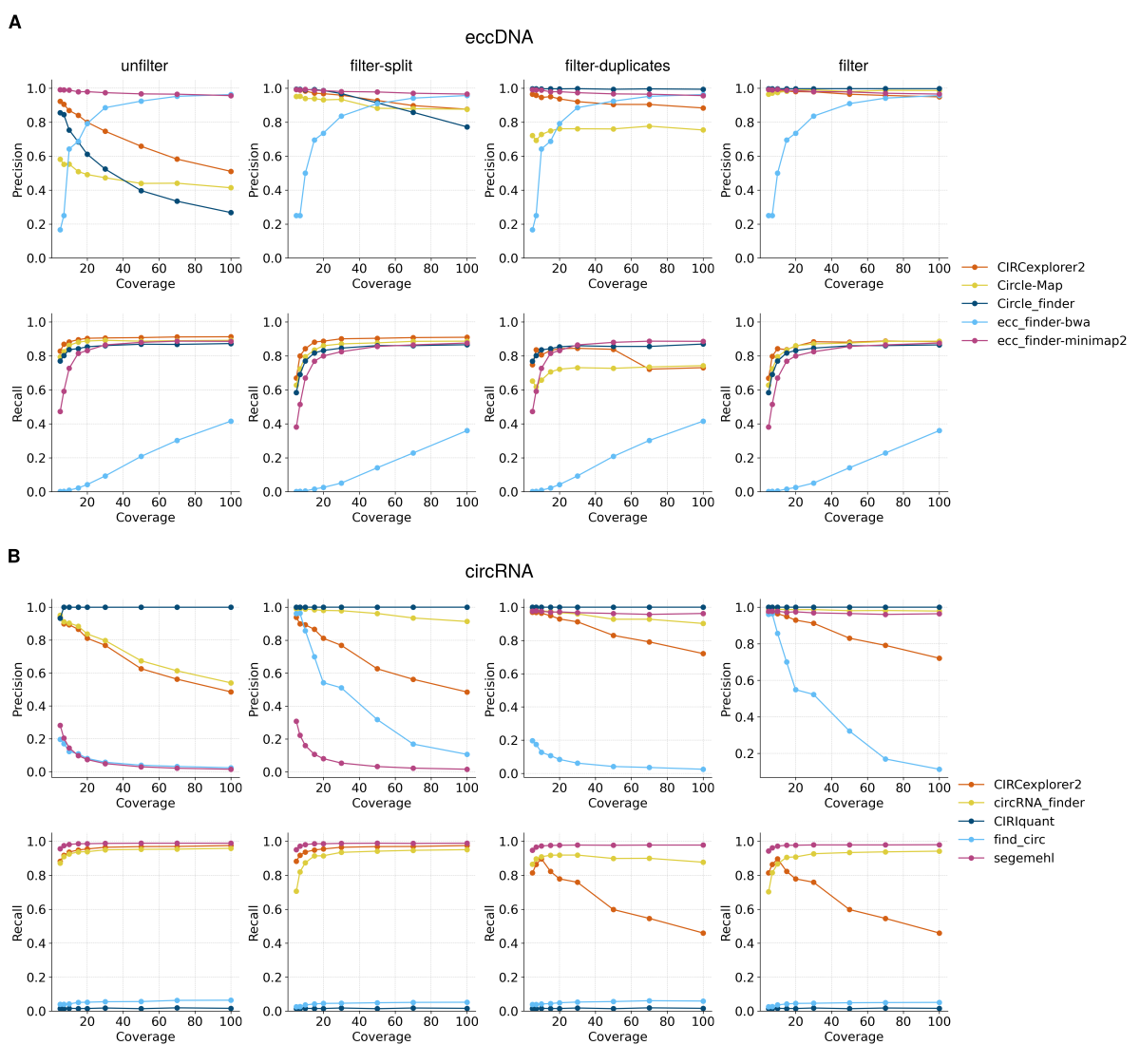

Supplement: giag017_Supplemental_Files [file giag017_supplemental_files.zip › supplementary1.png]

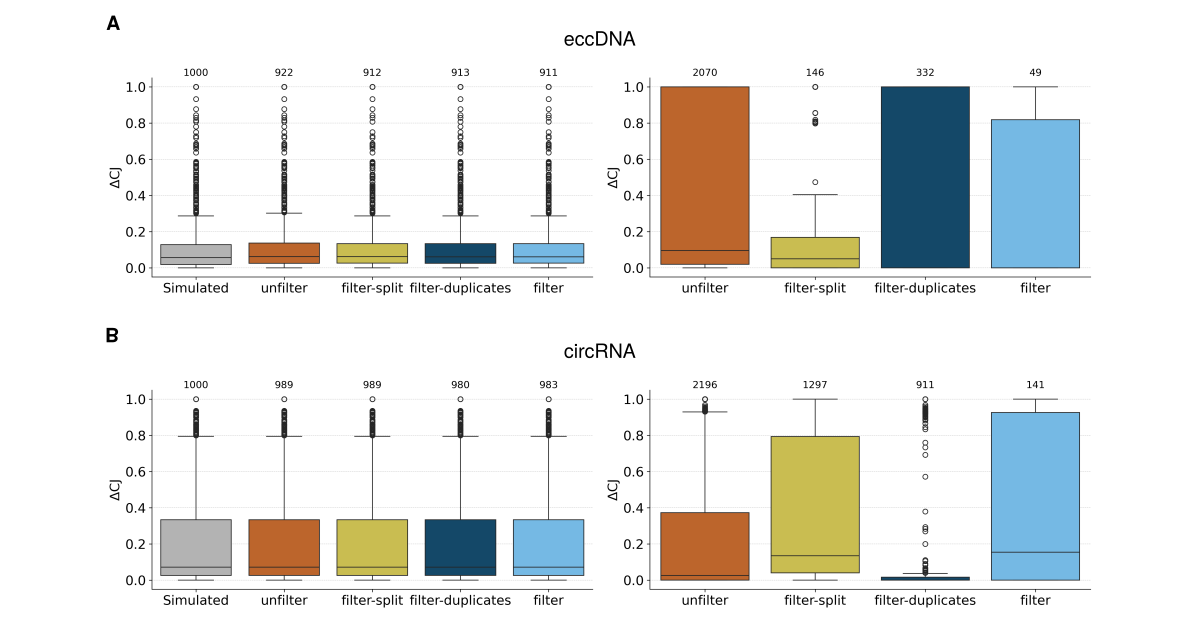

Supplement: giag017_Supplemental_Files [file giag017_supplemental_files.zip › supplementary10.png]

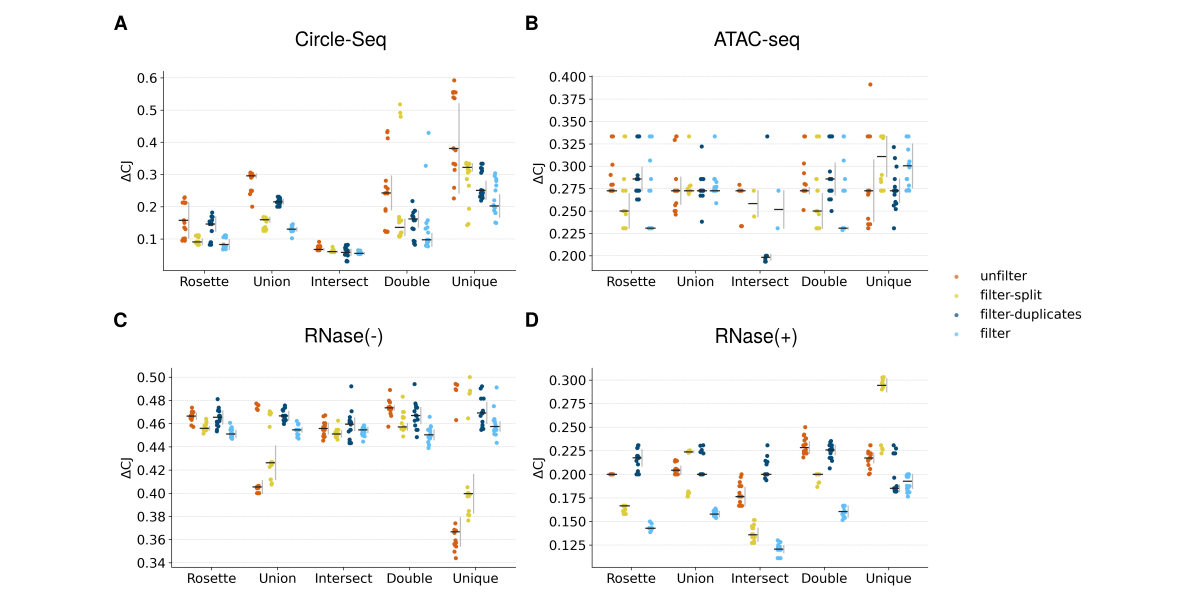

Supplement: giag017_Supplemental_Files [file giag017_supplemental_files.zip › supplementary11.png]

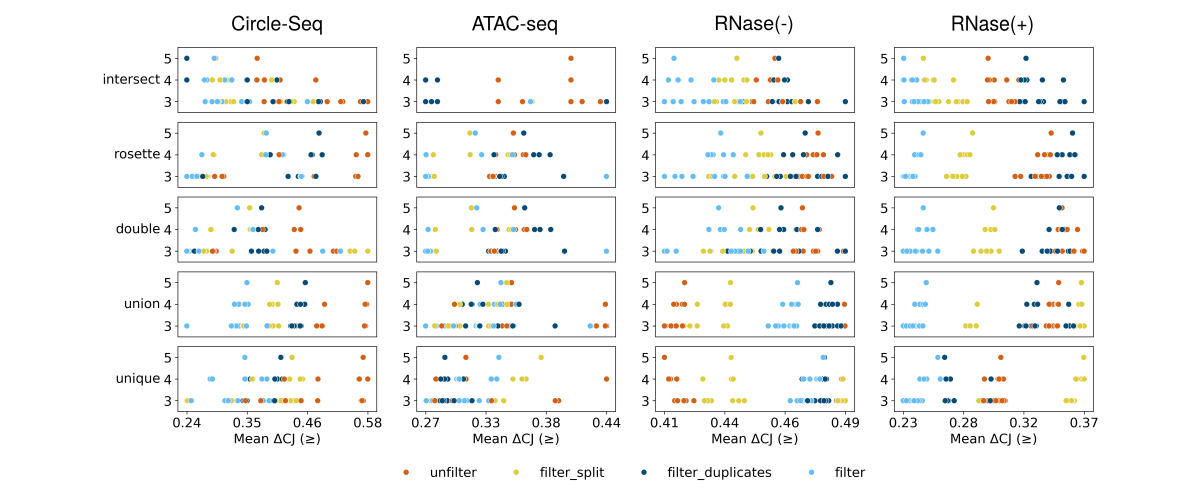

Supplement: giag017_Supplemental_Files [file giag017_supplemental_files.zip › supplementary12.png]

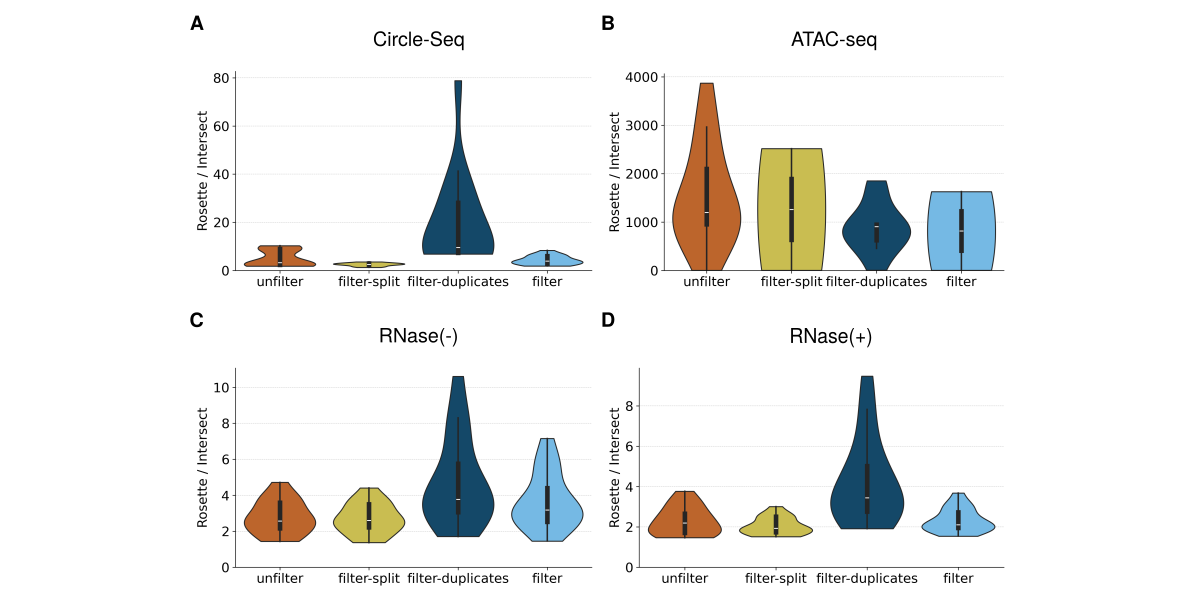

Supplement: giag017_Supplemental_Files [file giag017_supplemental_files.zip › supplementary13.png]

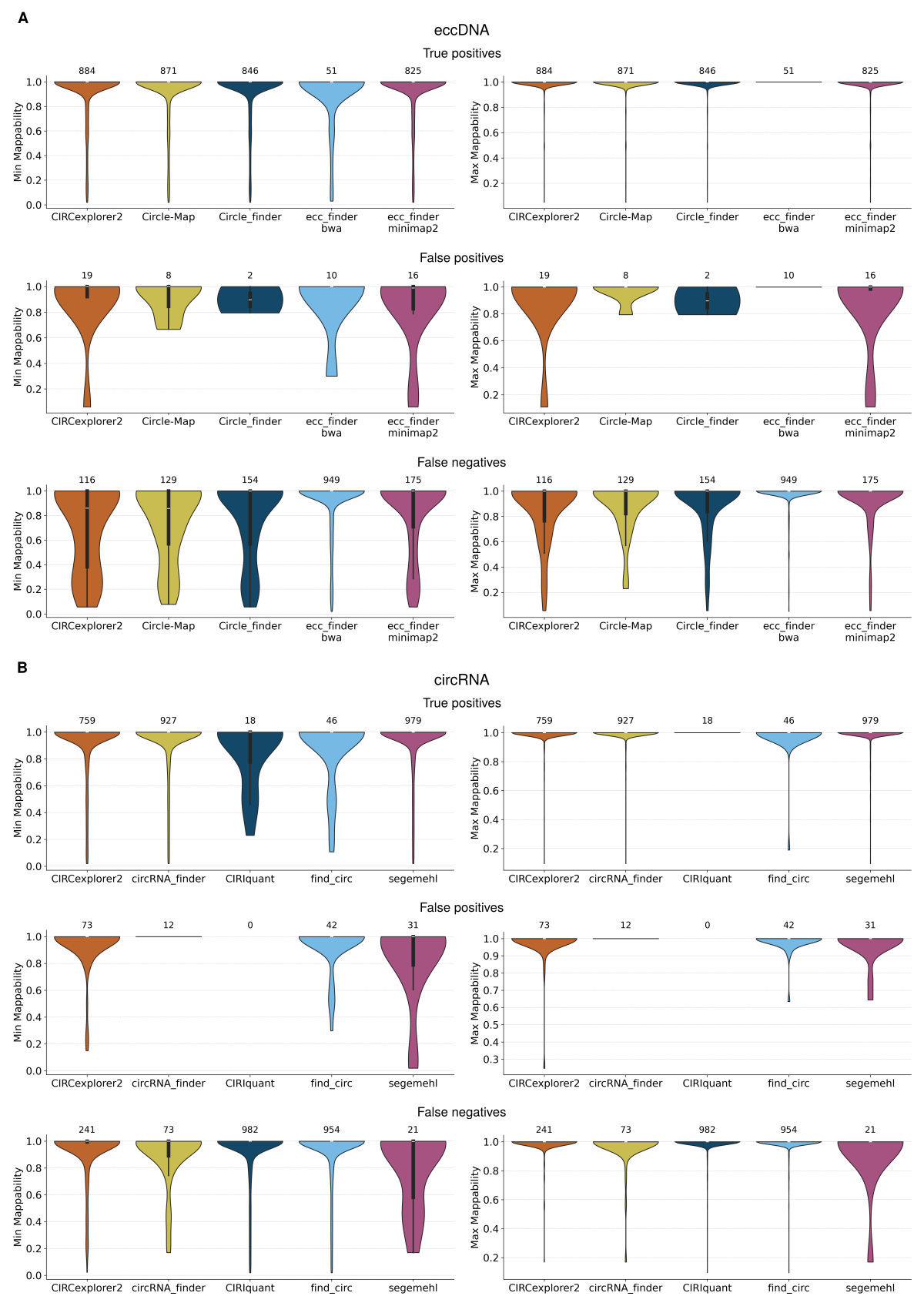

Supplement: giag017_Supplemental_Files [file giag017_supplemental_files.zip › supplementary14.png]

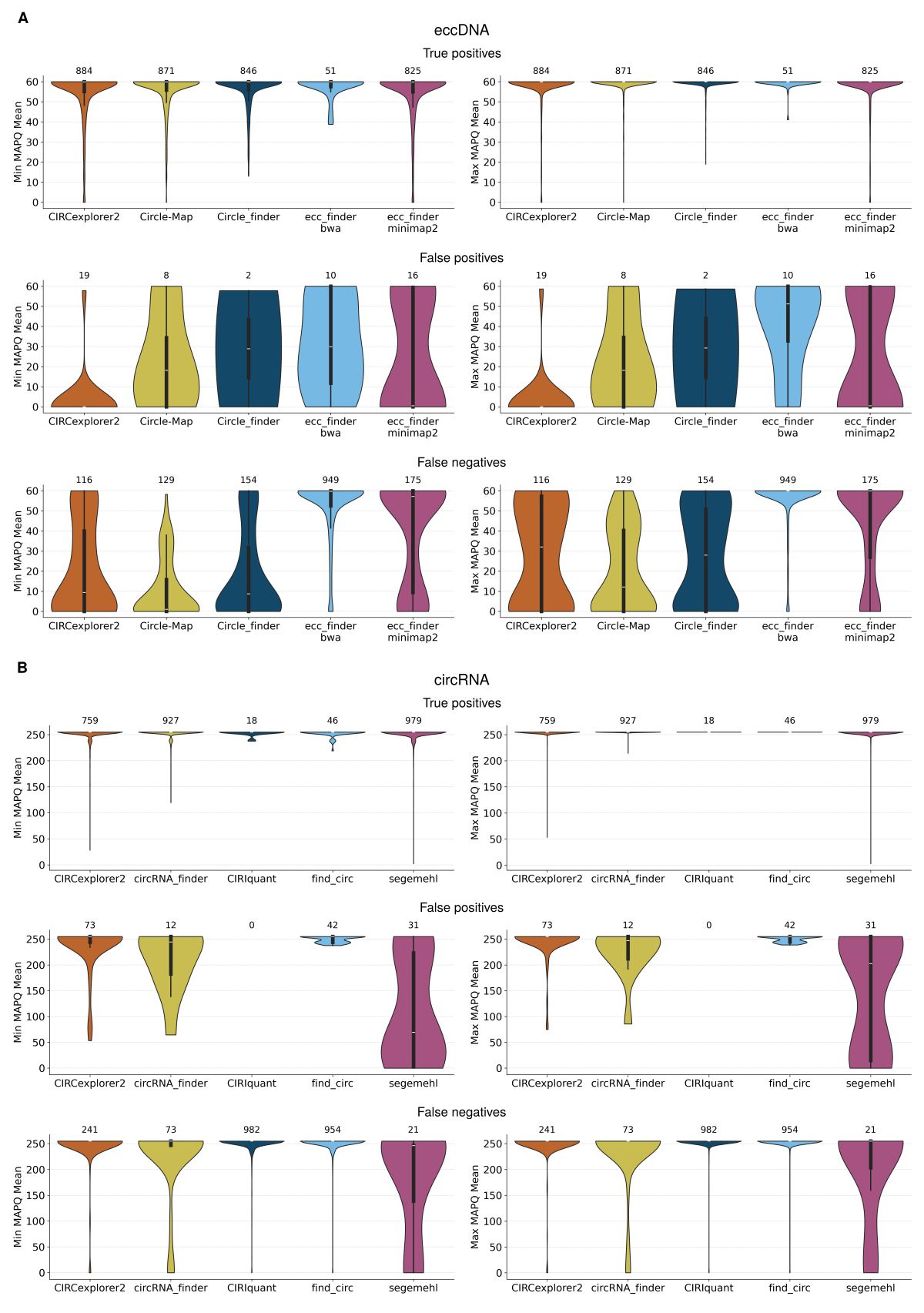

Supplement: giag017_Supplemental_Files [file giag017_supplemental_files.zip › supplementary15.png]

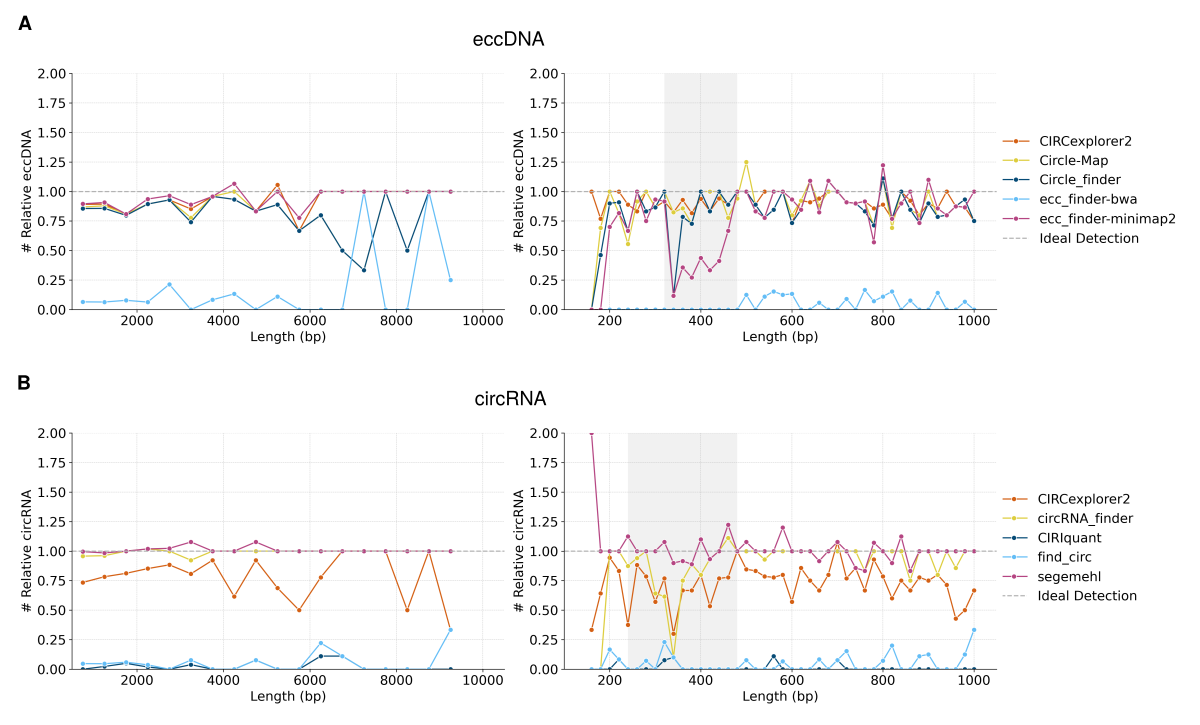

Supplement: giag017_Supplemental_Files [file giag017_supplemental_files.zip › supplementary2.png]

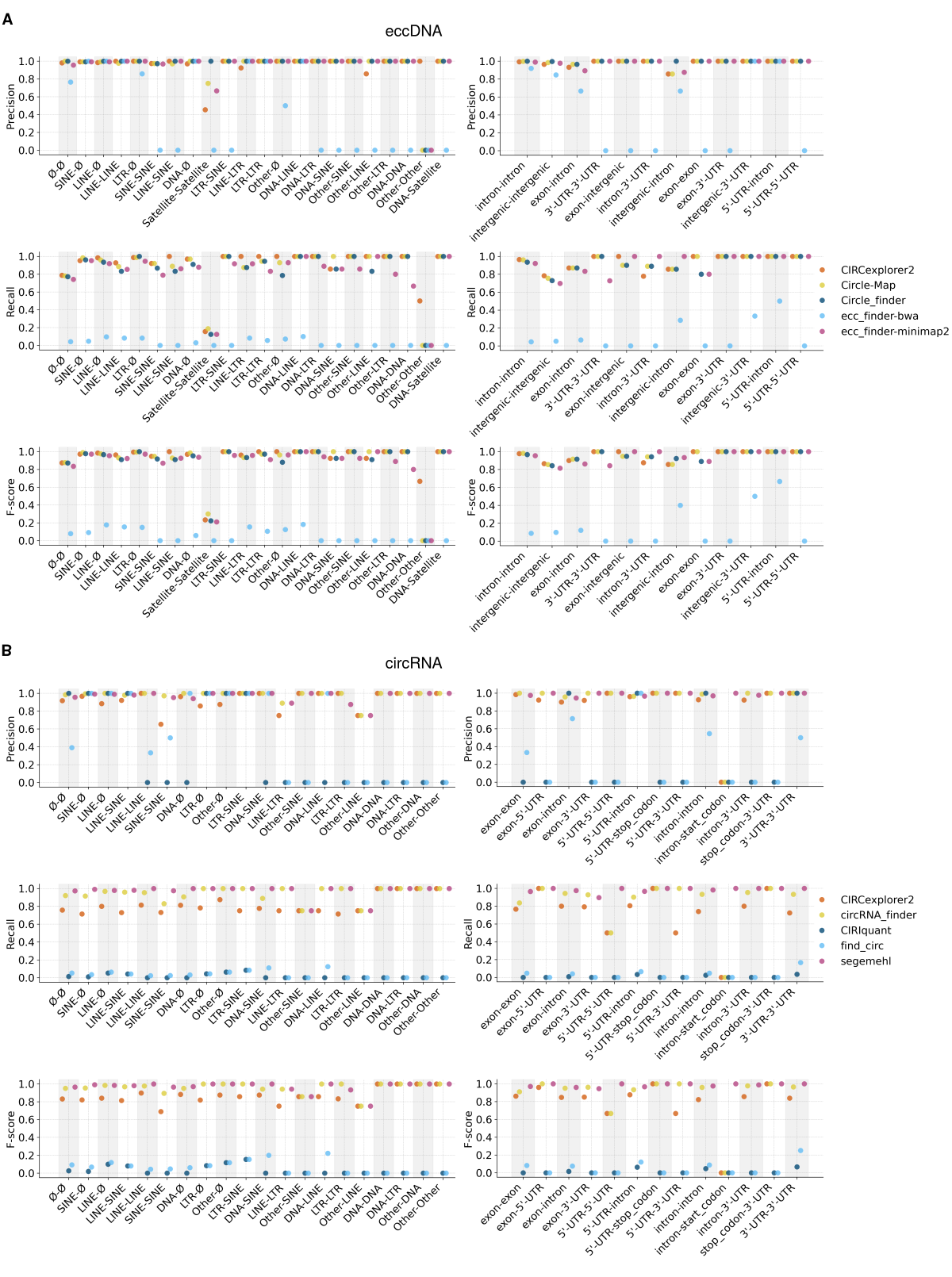

Supplement: giag017_Supplemental_Files [file giag017_supplemental_files.zip › supplementary3.png]

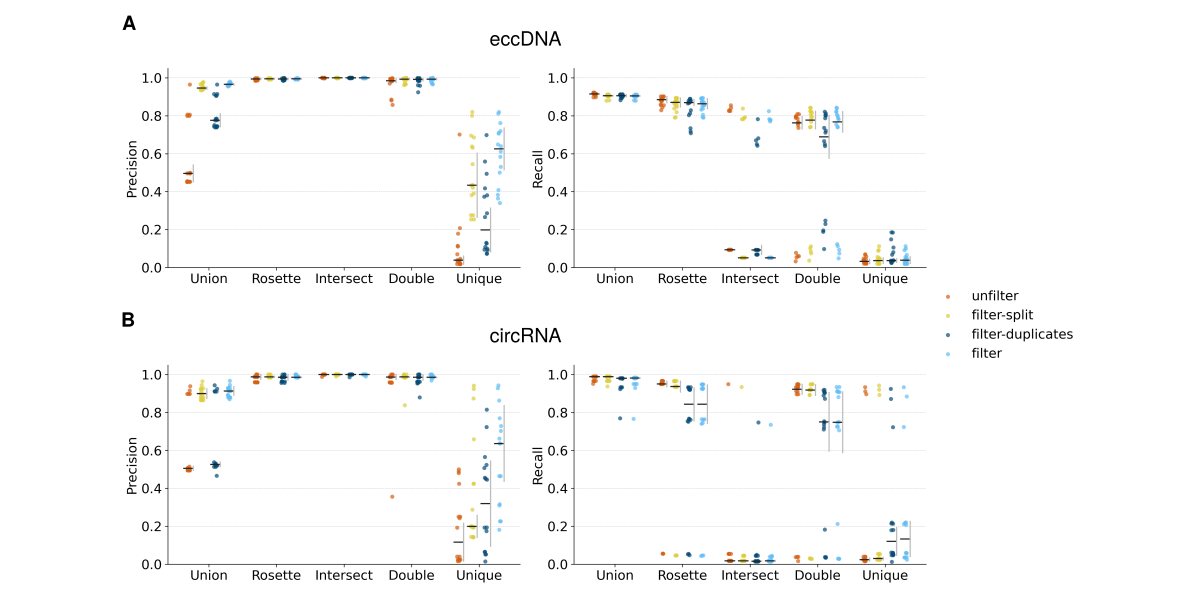

Supplement: giag017_Supplemental_Files [file giag017_supplemental_files.zip › supplementary4.png]

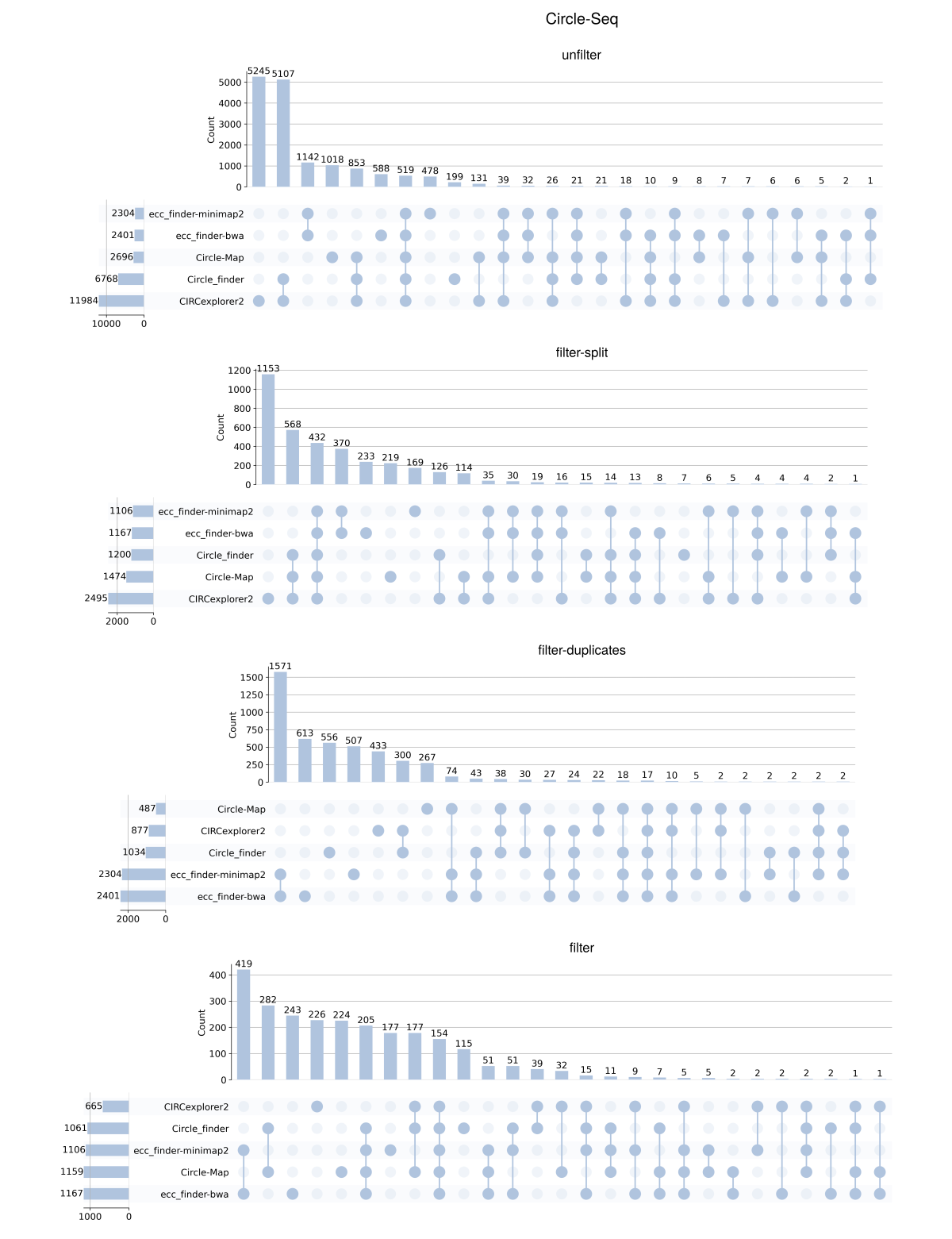

Supplement: giag017_Supplemental_Files [file giag017_supplemental_files.zip › supplementary5.png]

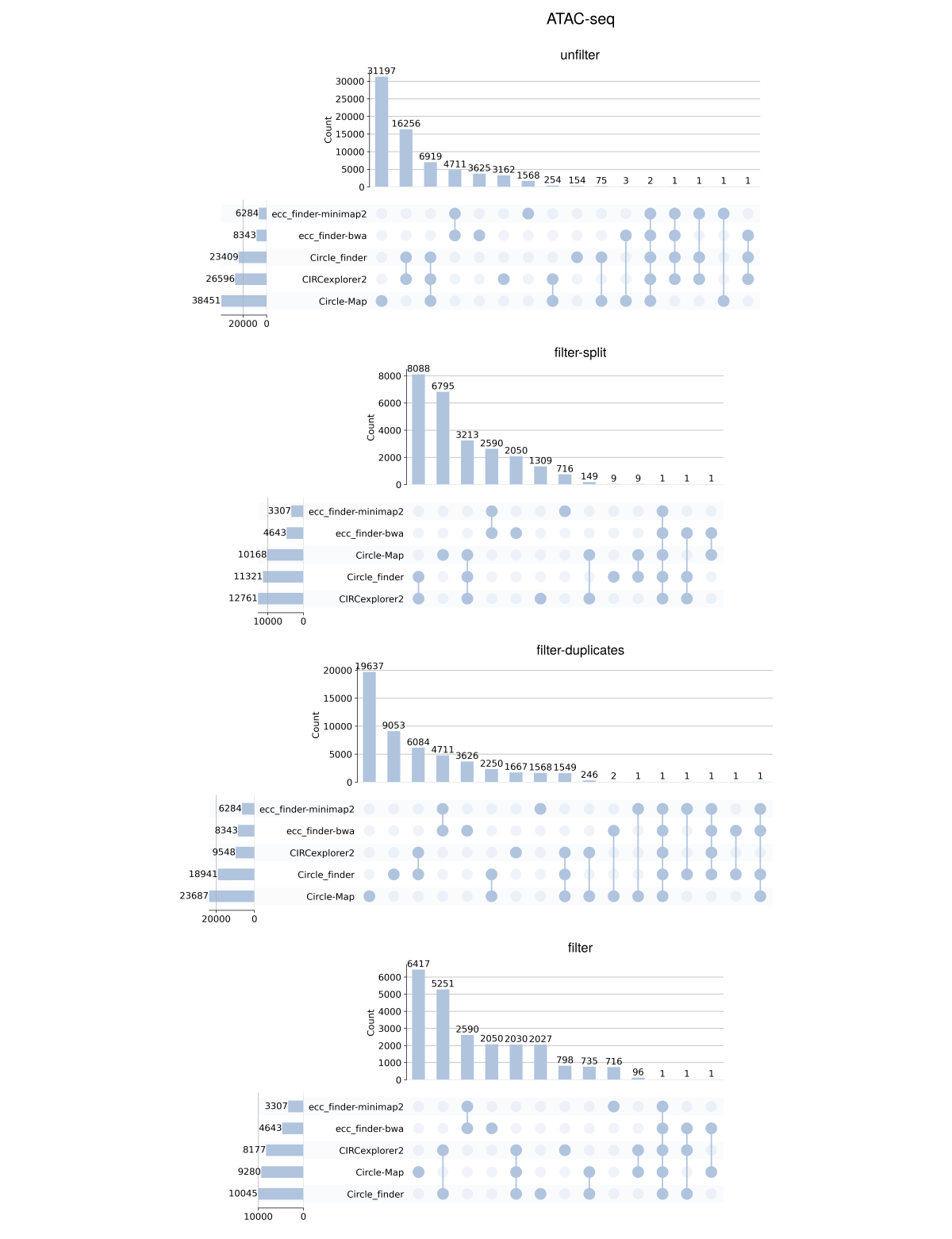

Supplement: giag017_Supplemental_Files [file giag017_supplemental_files.zip › supplementary6.png]

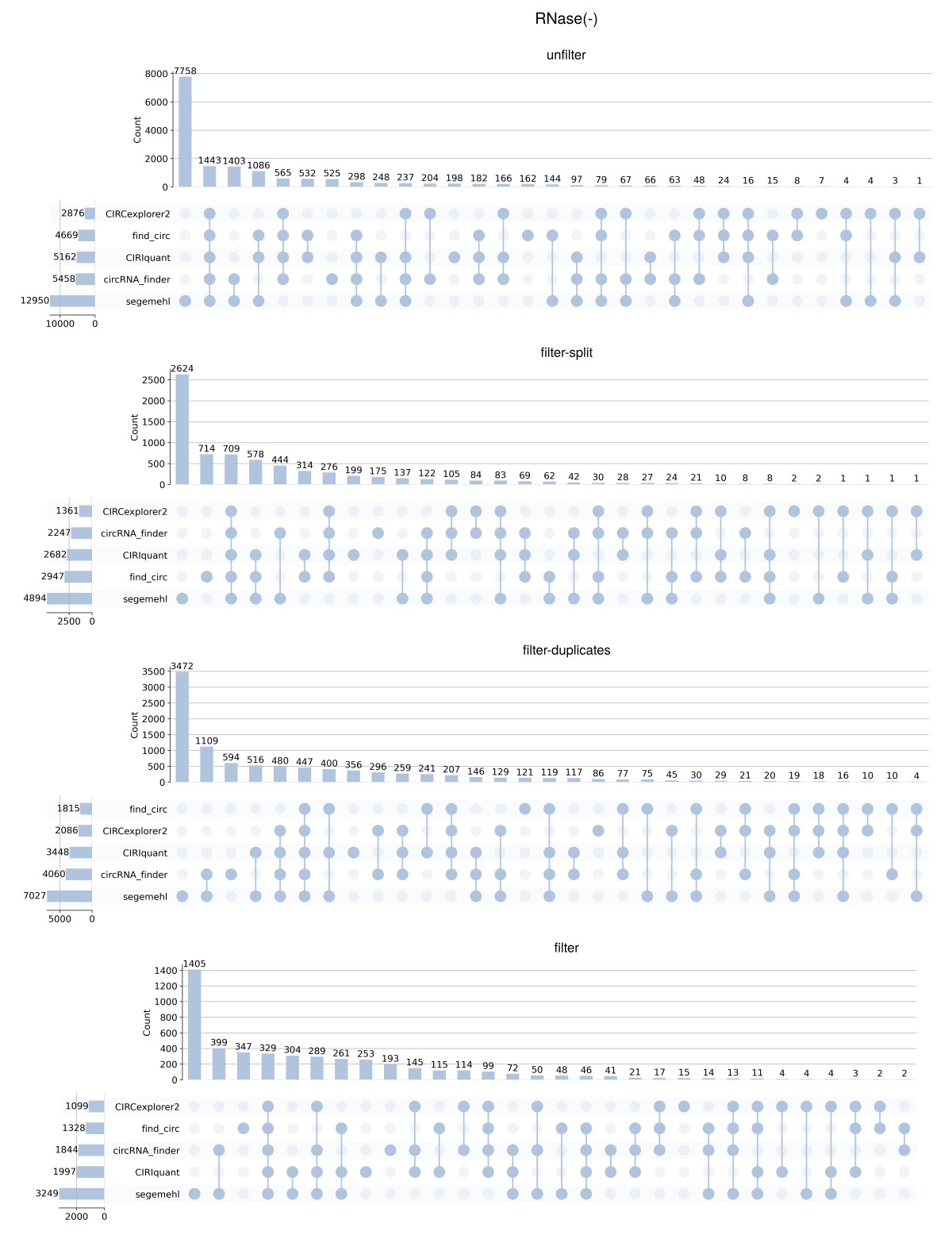

Supplement: giag017_Supplemental_Files [file giag017_supplemental_files.zip › supplementary7.png]

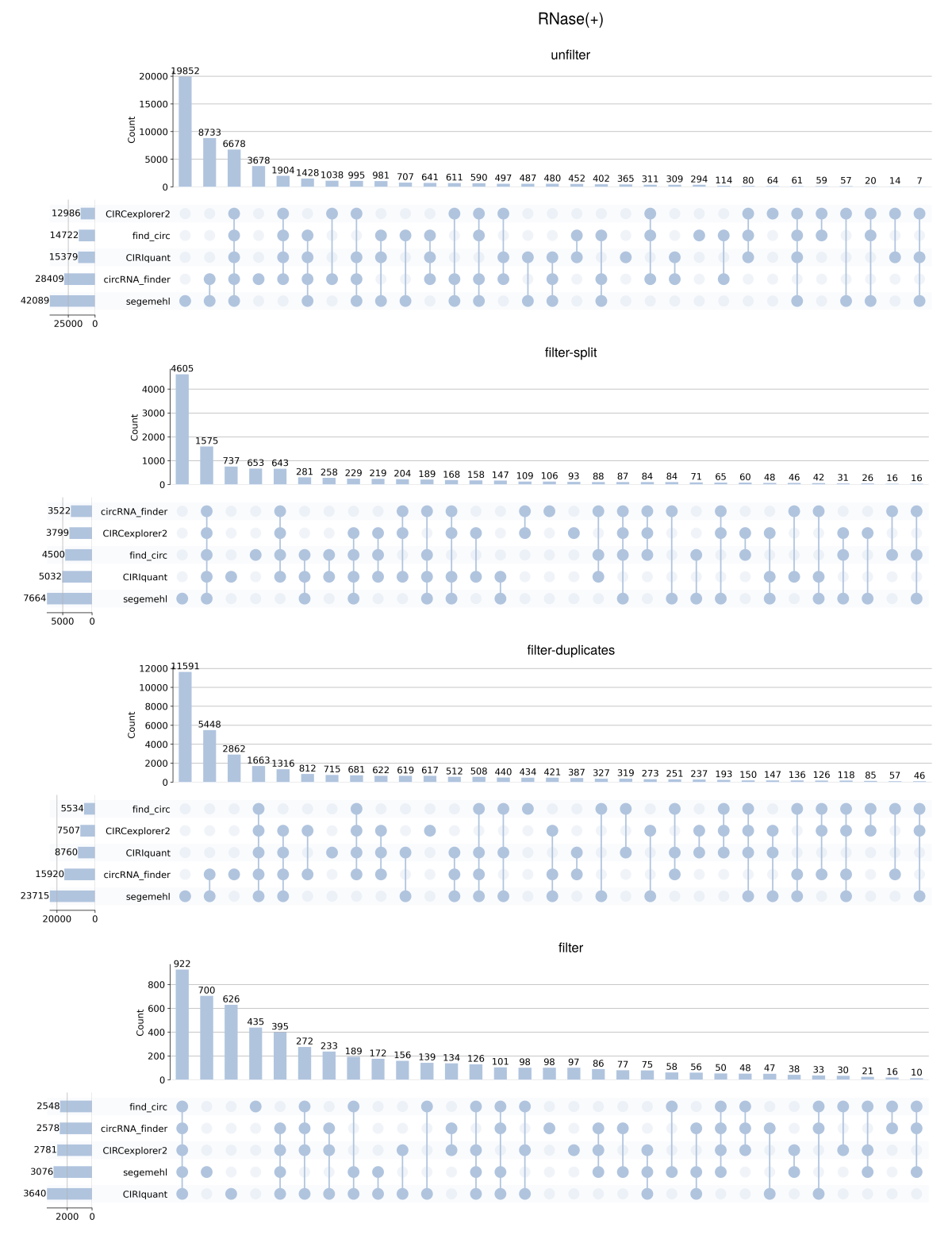

Supplement: giag017_Supplemental_Files [file giag017_supplemental_files.zip › supplementary8.png]

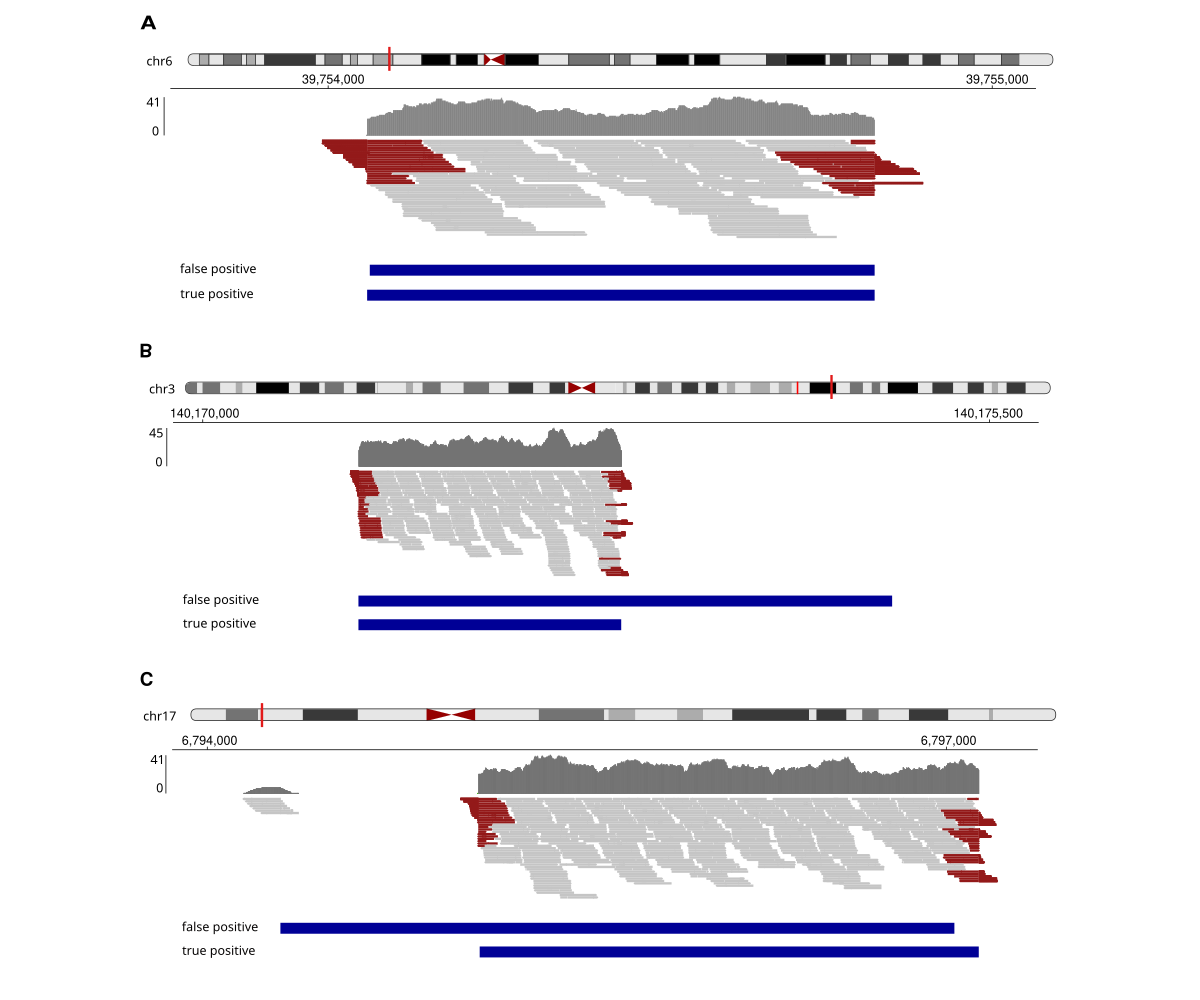

Supplement: giag017_Supplemental_Files [file giag017_supplemental_files.zip › supplementary9.png]
