# Supplementary material for: Comparative analysis of eccDNA and circRNA tools shows increased accuracy of tool combination
Source: Gigascience. 2026 Feb 25;15:giag017. doi: 10.1093/gigascience/giag017 (PMC13154841; doi:10.1093/gigascience/giag017)
Supplement: giag017_GIGA-D-25-00520_original_submission [file giag017_giga-d-25-00520_original_submission.pdf]

GigaScience

Comparative analysis of eccDNA and circRNA tools shows increased accuracy of tool combination

--Manuscript Draft--

**Manuscript Number:**	GIGA-D-25-00520
**Full Title:**	Comparative analysis of eccDNA and circRNA tools shows increased accuracy of tool combination
**Article Type:**	Research
**Funding Information:**	Hezkuntza, Hizkuntza Politika Eta Kultura Saila, Eusko Jaurlaritza (PRE_2025_1_0138) — Mr. Aitor Zabala<br>Eusko Jaurlaritza (IKUR-Nanoneuro) — Dr. Alex M. Ascension<br>Dietmar Hopp Stiftung — Dr. Iñigo Prada-Luengo<br>Instituto de Salud Carlos III — Dr. David Otaegui
**Abstract:**	Introduction: Circular nucleic acids such as extrachromosomal circular DNA (eccDNA) and circular RNA (circRNA) are increasingly recognized for their biological relevance and potential as biomarkers in disease contexts. Despite their growing importance, their detection remains challenging due to tool-specific biases, limited validation frameworks, and high variability in performance across datasets.<br>Methods: We benchmarked 10 circle detection tools across diverse conditions using both simulated and biological datasets. Our evaluation included classical performance metrics and a novel internal measure of read distribution symmetry (ΔCJ) to assess circle prediction confidence. We explored the impact of sequencing protocols, filtering strategies, and combined tool consensus.<br>Results: We found that detection accuracy was highly influenced by sequencing depth, alignment algorithm, and experimental enrichment protocols. ΔCJ proved effective in flagging potential false positive circles, showing improved accuracy of Intersect (circles detected by all tools) and Rosette (circles detected by ≥2 tools) combinations.<br>Discussion: This study offers a broad evaluation of circular detection tools, suggesting that the combination of ≥ 3 tools is necessary for a correct prediction. These insights will inform future experimental design and data analysis pipelines in both experimental and clinical settings.
**Corresponding Author:**	David Otaegui, Ph.D<br>Biogipuzkoa HRI<br>San Sebastián, Basque Country SPAIN
**Corresponding Author Secondary Information:**	
**Corresponding Author's Institution:**	Biogipuzkoa HRI
**Corresponding Author's Secondary Institution:**	
**First Author:**	Aitor Zabala
**First Author Secondary Information:**	
**Order of Authors:**	Aitor Zabala<br>Alex M. Ascension<br>Iñigo Prada-Luengo<br>David Otaegui
**Order of Authors Secondary Information:**	
**Additional Information:**	
**Question**	**Response**

Powered by Editorial Manager® and ProduXion Manager® from Aries Systems Corporation

Are you submitting this manuscript to a special series or article collection?	No
**Experimental design and statistics**<br><br>Full details of the experimental design and statistical methods used should be given in the Methods section, as detailed in our Minimum Standards Reporting Checklist. Information essential to interpreting the data presented should be made available in the figure legends.<br><br>Have you included all the information requested in your manuscript?	Yes
**Resources**<br><br>A description of all resources used, including antibodies, cell lines, animals and software tools, with enough information to allow them to be uniquely identified, should be included in the Methods section. Authors are strongly encouraged to cite Research Resource Identifiers (RRIDs) for antibodies, model organisms and tools, where possible.<br><br>Have you included the information requested as detailed in our Minimum Standards Reporting Checklist?	Yes
**Availability of data and materials**<br><br>All datasets and code on which the conclusions of the paper rely must be either included in your submission or deposited in publicly available repositories (where available and ethically appropriate), referencing such data using a unique identifier in the references and in the "Availability of Data and Materials" section of your manuscript.<br><br>Have you have met the above requirement as detailed in our Minimum	Yes

Standards Reporting Checklist?	
GigaScience has policies and guidelines in place for the use of generative AI-writing tools such as ChatGPT. If you have used such writing tools to assist with writing the manuscript this must be declared and cited in the text. Authors should not list AI-writing tools and other AI-assisted technologies as an author or co-author and should acknowledge that they are fully responsible for text generated or refined by AI-writing tools.<p><br><br>A summary of use (particularly in the introduction or among methods) needs to be included at the end of the paper, and the outputs should also be included as a supplementary file hosted in GigaDB or other open repositories. Please read our guidelines for more information. <p><br><br>By submitting to GigaScience, you are aware of the journal's AI-writing tools policy, and if you have declared use of such tools below, you have acknowledged this where appropriate in your manuscript and have made a summary of use and outputs available. <p><br><b>AI-assisted writing tools have been used in the preparation of this manuscript?	Yes

 Click here to access/download;LaTeX - Main Document (TeX file);main.tex

```
This is pdfTeX, Version 3.141592653-2.6-1.40.26 (TeX Live 2024)
(preloaded format=pdflatex 2024.8.2)  19 DEC 2025 07:30
entering extended mode
 restricted \write18 enabled.
 %&-line parsing enabled.
**main.tex
(./main.tex
LaTeX2e <2024-06-01> patch level 2
L3 programming layer <2024-05-27>

! LaTeX Error: File `oup-contemporary.cls' not found.

Type X to quit or <RETURN> to proceed,
or enter new name. (Default extension: cls)

Enter file name:
! Emergency stop.
<read *>

l.11 ^^M

*** (cannot \read from terminal in nonstop modes)


Here is how much of TeX's memory you used:
 19 strings out of 473583
 444 string characters out of 5732343
 1925908 words of memory out of 5000000
 23012 multiletter control sequences out of 15000+600000
 558069 words of font info for 36 fonts, out of 8000000 for 9000
 1141 hyphenation exceptions out of 8191
 19i,0n,29p,95b,17s stack positions out of
10000i,1000n,20000p,200000b,200000s
!  ==> Fatal error occurred, no output PDF file produced!
```

Manuscript

Click here to access/download;Manuscript;Circular_Detection.pdf

GigaScience, 2025, 1–32

doi: xx.xxxx/xxxx
Manuscript in Preparation
Paper

PAPER

Comparative analysis of eccDNA and circRNA tools shows increased accuracy of tool combination

Aitor Zabala[1,†], Alex M. Ascensión[1,†], Iñigo Prada-Luengo[2,3] and David Otaegui[1,4,*]

[1]Group of Neuroimmunology, Biogipuzkoa Health Research Institute, San Sebastián, Spain and [2]University of Copenhagen, Center for Health Data Science, Section for Health Data Science and Artificial Intelligence, Department of Public Health, Faculty of Health and Medical Sciences, Copenhagen, Denmark and [3]Rigshospitalet Copenhagen University Hospital, Center for Genomic Medicine, Copenhagen, Denmark and [4]Center for Biomedical Research Network in Neurodegenerative Diseases (CIBER-CIBERNED-ISCIII), Madrid, Spain

[*]davidangel.otaeguibichot@osakidetza.eus
[†]Contributed equally.

Abstract

Introduction: Circular nucleic acids such as extrachromosomal circular DNA (eccDNA) and circular RNA (circRNA) are increasingly recognized for their biological relevance and potential as biomarkers in disease contexts. Despite their growing importance, their detection remains challenging due to tool-specific biases, limited validation frameworks, and high variability in performance across datasets.
Methods: We benchmarked 10 circle detection tools across diverse conditions using both simulated and biological datasets. Our evaluation included classical performance metrics and a novel internal measure of read distribution symmetry (ΔCJ) to assess circle prediction confidence. We explored the impact of sequencing protocols, filtering strategies, and combined tool consensus.
Results: We found that detection accuracy was highly influenced by sequencing depth, alignment algorithm, and experimental enrichment protocols. ΔCJ proved effective in flagging potential false positive circles, showing improved accuracy of *Intersect* (circles detected by all tools) and *Rosette* (circles detected by $\geq$2 tools) combinations.
Discussion: This study offers a broad evaluation of circular detection tools, suggesting that the combination of $\geq$ 3 tools is necessary for a correct prediction. These insights will inform future experimental design and data analysis pipelines in both experimental and clinical settings.

Key words: extrachromosomal circular DNA; circular RNA; benchmark; multi-tool integration; bioinformatics

Introduction

Extrachromosomal circular DNA (eccDNA) and circular RNA (circRNA) are covalently closed-loop structures formed via DNA circularization events and the back-splicing process of precursor messenger RNA (pre-mRNA), respectively [1, 2]. Both types of molecules are common in eukaryotic organisms and are abundant in various types of cells and tissues [1, 3, 4, 5, 6, 7, 8]. Due to their association with various diseases, their potential use as disease biomarkers has garnered significant interest [9, 10, 11]. In this study, we focus on eccDNA defined as circular DNA elements in the small-to-moderate size range (tens to a few thousand base pairs). We distinguish these from larger, tumor-associated ecDNA elements (commonly >10 kbp) that are typically reported in oncogene amplification studies; because ecDNA and eccDNA differ in size, biogenesis and functional impact, we use the term eccDNA throughout to indicate the smaller class studied here.

eccDNA and circRNA isolation and detection poses several challenges. eccDNA and circRNAs are typically sequenced using targeted approaches, such as Circle-Seq, which is followed by nuclease

Compiled on: December 19, 2025.
Draft manuscript prepared by the author.

treatments to eliminate linear sequences and enrichment processes to amplify circular sequences [12, 13]. However, targeted sequencing is currently under debate, and alternative non-enriched techniques, including ATAC-seq and RNA-seq, are gaining significance [14, 15].

The prediction of genomic coordinates of eccDNA and circRNA circular junctions (CJ)–DNA breakpoint for eccDNA and backsplice junctions (BSJs) for circRNA–requires specialized algorithms capable of identifying reads that map to that CJ. First, reads are aligned to the reference genome and discordant reads are extracted. The unmapped reads are then remapped in reverse orientation to identify putative CJ connections. Various filtering criteria can be applied, such as the number of reads assigned to the CJ and the splicing signal that flanks the sites in the circRNA [16, 17]. For the detection of circRNA, in addition to traditional methods, a pseudoreference alignment approach can also be employed. In this approach, circular read candidates are aligned with a synthetic reference that includes circular sequences to identify and validate BSJs [18, 19].

Numerous computational software programs have been developed and tested for detecting eccDNA and circRNA. Insights from comparisons based on eccDNA [20, 21] and circRNA [22, 23, 24] revealed significant differences in the detection capabilities of these software, particularly in terms of the total number of circles identified. Consequently, addressing the high rate of false positives (FP) caused by technical artifacts or transcripts derived from uncommon events, such as exon duplication or trans-splicing events, remains a critical challenge [12, 25]. One way to mitigate this issue is by combining two or more prediction software tools, identifying only the circles that are shared between them [26]. Additionally, other software works at a lower level, merging read detection results from different tools [27]. In circRNA studies, this approach defines the so-called *bona fide* circles, increasing confidence in their detection.

Regarding the establishment of common protocols for circular analysis, *nf-core* [28] provides a collection of community-driven high-quality Nextflow [29] pipelines for analyzing eccDNA [30] and circRNA [16]. These pipelines can help develop standardized protocols for detecting circular molecules. Both pipelines are compatible with targeted sequencing methods as well as whole-genome sequencing (WGS) and ATAC-seq for eccDNA and RNA-seq for circRNA.

Although these tools are widely used, studies still lack a rigorous and standardized evaluation framework, leading to considerable variability in their results. This inconsistency is largely due to the fact that most benchmarks rely solely on *in silico* data, which often produces divergent outcomes. These discrepancies arise from: (1) sequencing artifacts, (2) differences in how circles are formed in repetitive regions—leading to higher FP rates, and (3) biological post-processing steps that cannot be fully replicated computationally [24, 31]. The reliance on *in silico* data is primarily due to the absence of a reliable proxy for assessing circle detection quality. As a result, most benchmarks simply compare detection outputs across tools and conditions, which is insufficient for evaluating performance on real biological data.

In this study, we present a comprehensive evaluation of five eccDNA and five circRNA detection tools using both *in silico* datasets with an array of coverages and circle sizes, as well as biological datasets produced with different methods. We show that moderate coverages (×10–×20), combined with split-read filters, minimize FP circles. To overcome the limitations of individual detection tools, we propose the *Rosette* combination, which retains only those circles supported by at least two tools, and achieves the optimal balance between precision and recall. To evaluate the accuracy of circle detection, we introduce the ΔCJ parameter—the discrepancy in read assignment to each side of the breakpoint—as a proxy for detection quality, and validate our approach on biological datasets, thereby enhancing the reliability of circular molecule quantification.

Methods

eccDNA and circRNA detection software

In this study, we compared 5 eccDNA detection software–CIRCexplorer2 (v2.3.8), Circle-Map (v1.1.4), Circle_finder, ecc_finder-bwa (v1.0.0), and ecc_finder-minimap2 (v1.0.0)–and 5 circRNA detection software–CIRCexplorer2 (v2.3.8), circRNA_finder (v1.2), CIRIquant (v2.1.0), find_circ (v1.2), and segemehl (v0.3.4)–. All of the software tools used in this study are integrated into the nf-core framework, with the exception of ecc_finder. We performed the detection of eccDNA using nf-core/circdna (v1.1.0) [30], and the detection of circRNA using nf-core/circrna (dev) [16]. Sequence reads were aligned to the human reference genome GRCh38 (NCBI) and the mouse reference genome GRCm38 (Ensembl).

For read alignment, eccDNA sequencing reads were mapped to the reference genome using BWA (v0.7.17-r1188), while circRNA sequencing reads were aligned using STAR (v2.7.11b). Alignments were performed against the human reference genome GRCh38 (NCBI) and the mouse reference genome GRCm38 (UCSC)

Although all software's detection is base on identifying the circular junction, their strategies for identifying circles is different.

eccDNA

CIRCexplorer2 is the upgraded version of CIRCexplorer [32]. It was primarily developed to detect circRNA, but it can also identify eccDNA. CIRCexplorer2 integrates additional aligner options beyond the original TopHat2 [33], including STAR [34], MapSplice [12] and segemehl, to accommodate different RNA-seq mapping preferences. CIRCexplorer2 alignes reads to the reference genome using various aligners and detects non-colinear reads. Next, CIRCexplorer2 analyzes these non-colinear alignments to detect the exact location of the CJ. Additionally, CIRCexplorer2 reanalyzes reads that were originally mapped to linear exon-exon junctions. For circRNA, it also performs *de novo* assembly of linear reads to discover novel exons and splicing events. Additionally, unmapped reads are realigned to capture any missed circular structures. In this study, DNA was mapped using BWA [35], and RNA was mapped using STAR. For *in silico* circRNA detection, we selected an intermediate non-annotated file due to the lack of detected circRNAs in the annotated output generated by the nf-core CIRCexplorer module.

Circle-Map identifies eccDNA breakpoints by utilizing discordantly mapped reads and mapping soft-clips using probabilistic models. First, Circle-Map detects eccDNA candidate reads, including discordant read pairs, soft-clipped reads, and hard-clipped reads, using the BWA aligner. Next, it constructs a breakpoint graph based on these candidate reads. Finally, soft-clipped reads are realigned using a probabilistic model to accurately determine the eccDNA breakpoints and achieve nucleotide-level resolution.

Circle_finder is designed to identify eccDNA from WGS and ATAC-seq data by analyzing read pairs using BWA. It collects all read pairs where one read maps uniquely to the genome in a contiguous manner and the other read maps as a split read flanking the mapped read. The start of the split read and the end of the contiguous read are then annotated as the start and end points of the eccDNA

ecc_finder detects circular breakpoints based on discordant reads and split reads. Once discordant reads and split reads are identified, only reads spanning the same boundary are retained to define the breakpoint. By default, ecc_finder uses the BWA aligner, but Minimap2 [36] can also be used.

circRNA

circRNA_finder uses STAR to directly identify chimeric junction reads from RNA-Seq data. After the initial alignment, the algorithm filters these chimeric reads to detect potential CJs based on predefined criteria. The filtering process includes evaluating the uniqueness of the mapped reads, allowing for a limited number of

mismatches, and ensuring that the distance between splice donor and acceptor sites is within a specified range.

CIRIquant extends the functionality of CIRI2 by implementing a pseudo-reference-based approach for circRNA detection. Initially, reads are aligned to the reference genome using BWA, and unmapped reads are considered as potential circRNA candidates. CIRIquant then uses CIRI2 to detect circRNAs, but it also supports BSJ bed files created by other software. A pseudo-reference consisting of circular sequences is generated, and all circular candidate reads are aligned to the pseudo-reference using HISAT2. Reads that map concordantly within a 10 bp region of the BSJ are classified as circular reads. Additionally, CIRIquant can perform RNase correction, linear RNA quantification, and circRNA differential expression analysis.

find_circ uses a segment-based approach to identify circRNA. First, reads are mapped to the reference genome using Bowtie2, and reads that align contiguously are discarded. From the remaining reads, 20 nucleotides from both ends are extracted and aligned to obtain unique anchor positions within spliced exons. Anchors that align in the reverse orientation are identified as circRNAs. The anchor alignments are then extended to the BSJ, flanked by GU/AG splice sites.

Segemehl is able to identify multiple types of splice junctions. It aligns RNA-Seq reads to a reference genome while accounting for complex splicing patterns that are characteristic of circular RNAs. The software detects chimeric reads that span back-splice junctions, where the 3' end of one exon is joined to the 5' end of another in a circular fashion.

in silico datasets

CircleSim

CircleSim is a specific simulation software for circular and linear reads. It is implemented in Python 3 and consists of three modules: 1) *coordinates*: generates coordinates based on a provided length distribution; 2) *reads*: simulates circular or linear short-read sequencing; and 3) *join*: merges circular and linear FASTQ files.

The *coordinates* module selects a chromosome at random based on length-associated probabilities. For both DNA and RNA, the first nucleotide of the CJ is randomly chosen across the entire genome and transcriptome, respectively. The position of the second nucleotide is determined by the length of the circular region, which can be modeled using either a uniform or a lognormal distribution. The uniform distribution is defined by specified minimum and maximum lengths, while the lognormal distribution is characterized by its mean and standard deviation, with options to set minimum and maximum lengths as well.

The *reads* module simulates sequencing based on the read and insert lengths, and a coverage defined as:

$$\text{coverage} = \frac{\text{n\_reads} \cdot \text{reads\_length} \cdot 2}{\text{circle\_length}}$$

For circular molecules, a nucleotide within the circle is selected randomly, and the distance to the CJ determines whether the read is concordant, discordant, or a split-read. Concordant reads are those mapped in the expected orientation, discordant reads are mapped in the opposite orientation, and split-reads have an unmapped portion because they span across the CJ. *CircleSim* includes an option to increase the proportion of reads near the CJ. For linear molecules, the start of the read is randomly selected between the start of the sequence and the position defined as the end of the sequence minus the insert length, ensuring that all reads are concordant.

The source code is released under the MIT license and is freely available at https://github.com/ZabalaAitor/CircleSim.

in silico datasets

We used *CircleSim* (v1.0.0) to generate 1,000 eccDNAs and circRNAs from the canonical chromosomes and transcripts for the GRCh38.p14 version (NCBI) of the human genome, respectively. The circles were simulated using a log-normal size distribution implemented in the *scipy.stats.lognorm* function with parameters $s = 1$, $loc = 0$, and $scale = 1000$ and circle lengths ranging from 175 bp to 10,000 bp. The reads were simulated based on short-read sequencing with a read length of 150 bp, an insert length of 500 bp, a sequencing error rate of 0.001, and a mutation rate of 0.01 under the Kimura mutation model, with coverage depths of ×5, ×7, ×10, ×15, ×20, ×30, ×50, ×70, and ×100.

Biological datasets

We downloaded a dataset from human muscle tissue from SRA database (accesion number SRR6315430), where eccDNA was enriched and sequenced using Circle-Seq [5]. We also used a dataset generated by The Chinese University of Hong Kong (CUHK) Circulating Nucleic Acids Research Group from a knockout mouse models with deficiencies in deoxyribonuclease 1 like 3 (DNASE1L3) from EGA database (accesion number EGAS00001005873), where cell-free eccDNA (cf-eccDNA) was sequenced using ATAC-seq [37] sequencing. These two datasets are labelled as "Circle-Seq" and "ATAC-seq" correspondingly. For circRNA, we downloaded two HELA datasets [38] from SRA database: an original sample (accesion number SRR1637090) and the corresponding sample after RNase R treatment (accesion number SRR1636986), labelled "RNase(-)" and "RNase(+)". We used 'prefetch' and 'fasterq-dump' from the SRA Toolkit (https://github.com/ncbi/sra-toolsv2.9.1) to download and convert the SRA files to FASTQ format.

The number of reads associated to each dataset is the following: Circle-Seq - 12,829,402; ATAC-seq - 12,287,079; RNase(-) - 35,685,310; RNase(+) - 23,505,713.

Circle filtering strategies

To improve the reliability of predicted circular elements, we implemented four filtering strategies: *unfilter*, *filter-split*, *filter-duplicates*, *filter*. These strategies aim to reduce FPs and emphasize consistently detected circles across different algorithms. The filtering strategies used in this study are described below:

- ***unfilter***: Includes all raw detections without applying any filtering criteria.
- ***filter-split***: Retains only circles supported by at least two split reads.
- ***filter-duplicates***: Removes overlapping circles. Two circles are considered overlapping if their coordinate-defined regions share at least one base. Among overlapping circles, the one with the highest number of supporting split reads is retained. If split read information is unavailable, the longest circle is kept.
- ***filter***: Applies both *filter-split* and *filter-duplicates*. Specifically, it first retains circles with at least two supporting split reads, and then removes overlapping circles as described above.

All tools used in this study provide split read information, except for *CIRCexplorer2* in the context of *in silico* circRNA detection, in which split information is not considered.

Circle combination strategies

To assess the reliability of circle detection across multiple tools, we evaluated the performance of different tool combination strategies (**Figure 1**):

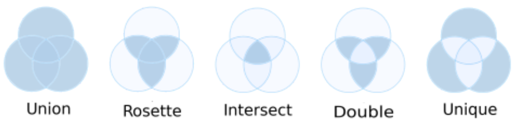

Figure 1. Description of the combining strategies. Visualization of the different combining strategies: *Union*, *Rosette*, *Intersect*, *Double*, and *Unique*.

- ***Rosette***: Includes circles detected by at least two different tools in a combination of three or more tools.
- ***Union***: Includes all circles detected by any of the tools.
- ***Intersect***: Includes only circles detected by all tools in the set.
- ***Unique***: Includes circles detected by only one tool.
- ***Double***: Includes circles detected by at least two tools, excluding both *Unique* and *Intersect* detections.

The combinations shown in the main plots are based on sets of three or more tools, in accordance with the minimum requirement of three tools for the *Rosette* strategy. However, two tool combinations for *Union*, *Intersect*, and *Unique* are included in the Supplementary Material for completeness.

Statistical analysis of *in silico* data

Base metrics

A threshold of 20 bp between the analyzed circles was applied to determine if two circles were considered the same. Detection accuracy in simulated data was analyzed using precision, recall, and F-score metrics.

$$\text{Precision} = \frac{TP}{TP + FP}$$

$$\text{Recall} = \frac{TP}{TP + FN}$$

$$\text{F-score} = \frac{2 \cdot \text{Precision} \cdot \text{Recall}}{\text{Precision} + \text{Recall}}$$

TP = true positives, FP = false positives, FN = false negatives.

Circular junction detection precision

To evaluate the offset of the CJ detection, we calculated the ratio of number of circles detected with an offset of 1 bp ($\text{eccDNA}_{T=1}$ for eccDNA and $\text{circRNA}_{T=1}$ for circRNA) and the maximum number of circles detected (eccDNA_{max} for eccDNA and circRNA_{max} for circRNA). Higher ratios indicate that most circles are detected with only a small offset of 1bp, while lower ratios indicate that a higher offset is required.

Length distribution

The distribution of eccDNA and circRNA lengths was analyzed and compared using the Kolmogorov-Smirnov (KS) test. To account for the non-uniformity of the distributions, we first examined the full range of circle lengths (175–10,000 bp). We then focused specifically on short circles, defined as those within the 175–1,000 bp range. Finally, we identified a specific length interval, referred to as the “square” range (shown in **Figure 4**), where the observed distribution was lower compared to the simulated distribution. The circular length distribution was plotted using both absolute and relative counts. The relative counts were calculated by normalizing against the counts from the simulated data. To enhance the clarity of the distribution plots, a sliding window of size 5 was applied to smooth the distribution curves. This technique allowed for more precise visualization of the trends across different tools, facilitating the comparison between predicted lengths and the ground truth, represented by the simulated circles. For this analysis, we used *in silico* datasets generated at ×30 coverage, ensuring consistency across comparisons.

Repeat element annotation

For the repeat element analysis, we utilized the GRCh38 (NCBI) genome assembly in conjunction with RepeatMasker open (v4.0.5) and the Repeat Library (January 31, 2014). The RepeatMasker software, using the specified library, allowed us to identify and classify repetitive elements within the genome. Repeat elements were categorized into several classes, including *LINEs* (Long Interspersed Nuclear Elements), *SINEs* (Short Interspersed Nuclear Elements), *DNA* (DNA transposons), *satellite* (satellite DNA), and *other* elements such as *Long Terminal Repeats (LTR)*, *simple repeats*, *low-complexity regions*, *small nuclear RNA (snRNA)*, and *unknown* elements. Junctions that did not overlap with any annotated repeat were labeled as *non-repetitive* (∅). For this analysis, we used *in silico* datasets generated at ×30 coverage, ensuring consistency across comparisons. This analysis was performed to ensure comprehensive annotation of repeat sequences at each coordinate of the CJ. For this analysis, we used *in silico* datasets generated at ×30 coverage.

Genomic element annotation

For the genomic element analysis, we used the GFF annotation file corresponding to the GRCh38 version (NCBI) of the human genome. This analysis provides a comprehensive annotation of genomic elements at each CJ coordinate. Coordinates were annotated based on detailed genomic features, including *3’-UTR (3’ Untranslated region)*, *5’-UTR (5’ Untranslated region)*, and *other* elements such as *start codon*, *stop codon*, and *selenocysteine positions*. Coordinates not annotated in this step were evaluated for overlap with *exon* regions. Those that still remained unannotated were checked against *intronic* regions. Finally, any coordinates not matching any of the above categories were retained as *intergenic*. For this analysis, we used *in silico* datasets generated at ×30 coverage.

Statistical analysis of biological data

Similarity analysis

eccDNA and circRNA detection results were visualized using UpSet plots [39], which allow visualization of overlapping circles defined with a threshold of 20 bp.

Circular junction difference (△CJ)

When a read spans the CJ, the split, unmapped portion of the read can be associated either with the left or right side of the junction. Assuming both sides are equally likely, a strong imbalance in read association suggests potential artifacts, as the supporting reads may originate from other circles or misalignments.

Based on this hypothesis, if N is the number of reads spanning a circle’s CJ, k_L is the number assigned to the left side, and $k_R = N - k_L$ to the right side, we define the metric △CJ as:

$$\triangle\text{CJ} = \frac{|k_L - k_R|}{N}$$

To obtain k_L and k_R, we extracted reads overlapping windows

centered on the circle start and end positions, respectively. Each window extended $N_{\text{offset}} = 20$ nucleotides upstream and downstream of the junction coordinate to capture all potentially spanning reads. Unique read identifiers were collected within these windows from the aligned BAM file using the `pysam` package for efficient read retrieval. The total number of CJ reads, N, corresponds to the union of reads found in both windows.

To improve robustness against alignment artifacts, ΔCJ integrates two complementary corrections: junction region mappability bias and read-level mapping quality weighting.

- ***Junction region mappability***: The assumption of equal mapping probability (p=0.5) for both sides of the junction does not hold when local sequence mappability differs. To account for this, the expected probability of left-side support was estimated from a mappability track:

$$p_L = \frac{M_L}{M_L + M_R}, \quad p_R = 1 - p_L$$

  where M_L and M_R represent the average mappability scores over 30-nucleotide regions located immediately inside the circle at each junction boundary. Mappability values were extracted from UCSC 50-mer multi-track bigWig files (https://hgdownload.soe.ucsc.edu/gbdb/hs1/hoffmanMappability/) using the `pyBigWig` library.
  These scores were used to set the expected probability parameter for a two-sided binomial test assessing whether the observed left/right distribution significantly deviated from the expected mappability-driven balance.
- ***Read-level mapping quality weighting***: To reduce the influence of poorly aligned or multi-mapped reads, each read i contributes to the counts k_L or k_R according to its individual mapping quality (MAPQ_i). Each read was assigned a weight $w_i \in [0, 1]$ based on the probability of correct alignment derived from its MAPQ value:

$$w_i = 1 - 10^{-\frac{\text{MAPQ}_i}{10}}$$

  This probabilistic weighting reflects the exponentially decreasing error probability encoded in MAPQ values, thus giving more nuanced confidence weights to individual reads instead of a linear approximation.
  The effective read counts were obtained by summing the weights of all reads overlapping each junction side:

$$k_L = \left\lceil \sum_{i \in CJ_1} w_i \right\rceil, \quad k_R = \left\lceil \sum_{i \in CJ_2} w_i \right\rceil, \quad N = k_L + k_R$$

  and the junction imbalance metric was computed as:

$$\Delta\text{CJ} = \frac{|k_L - k_R|}{N}$$

Values of ΔCJ close to 0 indicate a balanced read distribution, whereas ΔCJ = 1 means that all reads associate with only one side of the junction. Of note, the ceiling function is used to approximate the number of reads to a non-zero integer, which is required for the following steps.

While informative, this metric has limitations, particularly at low N, where extreme ΔCJ values might occur. To address this, we modeled the probability of observing a particular distribution of reads (k_L and k_R) to identify circles where such probabilities are exceedingly low.

The number of reads associated with one side of the CJ, X, can be modeled using a binomial distribution: $X \sim B(N, 0.5)$ for the ideal scenario where mappability is not considered ($M_L = M_R \rightarrow p_L = p_R = 0.5$). Thus:

$$P(X = k) = \binom{N}{k} \cdot 0.5^k \cdot 0.5^{N-k} = \binom{N}{k} \cdot 0.5^N$$

Considering that assignment to either side of the CJ is arbitrary, the final distribution must account for symmetry:

$$P(X = k) = \left(\binom{N}{k} + \binom{N}{N-k}\right) \cdot 0.5^N = 2\binom{N}{k} \cdot 0.5^N \quad , \quad k = \{0, ..., N/2\}$$

Thus, the cumulative probability of assigning up to k reads out of N to one side of the CJ is:

$$p = P(X \le k) = 2 \cdot 0.5^N \sum_{i=0}^{k} \binom{N}{i}$$

For even N values, the cumulative distribution can exceed 1 due to double-counting at the midpoint $P(X = N/2)$ For example, when $N = 6$ and $k = 3$, the cumulative probability is: $p = 2 \cdot 0.5^6 (\binom{6}{0} + \binom{6}{1} + \binom{6}{2} + \binom{6}{3}) = 1.3125$. This happens because $P(X = 3)$ is counted twice. In contrast, when $N = 7$, for $k = 3$, $p = 2 \cdot 0.5^6 (\binom{7}{0} + \binom{7}{1} + \binom{7}{2} + \binom{7}{3}) = 1$, as the complement of $\binom{7}{3}$ is $\binom{7}{4}$. Thus, in even cases where $k = N/2$, the cumulative probability is set to 1 since midpoints are not relevant for identifying outliers, which are our primary interest.

For scenarios with unequal mappabilities, probabilities are calculated in a similar fashion. In this case, two variables arise: X_L, the number of k_L reads, which follows a binomial distribution $B(k, p_L)$; and X_R, the number of k_R reads, which follows a binomial distribution$B(k, p_R)$. Thus:

$$P(X_L = k_L) = \binom{k}{k_L} p_L^{k_L} (1 - p_L)^{k-k_L}$$
$$P(X_R = k_R) = \binom{k}{k_R} p_R^{k_R} (1 - p_R)^{k-k_R}$$

Considering that both $k_L | k_R$ and $p_L | p_R$ pairs are related; distributions associated to X_L and X_R are also related:

$$\begin{aligned} P(X_L = k_L) &= \binom{k}{k_L} p_L^{k_L} (1 - p_L)^{k-k_L} \\ &= \frac{k!}{k_L!(k - k_L)!} p_L^{k_L} (1 - p_L)^{k-k_L} \\ &= \frac{k!}{k_L! k_R!} p_L^{k_L} p_R^{k_R} \\ &= \frac{k!}{(k - k_R)! k_R!} (1 - p_R)^{k-k_R} p_R^{k_R} \\ &= \binom{k}{k_R} p_R^{k_R} (1 - p_R)^{k-k_R} = P(X_R = k_R) \end{aligned}$$

Therefore, since $P(X_L = k_L) = P(X_R = k_R)$, the cumulative distributions $P(X_L \le k_L)$ and $P(X_R \ge k_R)$ are equivalent.

Thus, the cumulative probability of assigning up to k_L reads to the left side of the CJ and up to k_R reads to the right side of the CJ can be computed using only one of the sides (e.g. left):

$$p = P(X_L \le k_L) + P(X_R \ge k_R) = 2 \cdot P(X_L \le k_L) = 2 \sum_{i=0}^{k_L} \binom{k}{i} p_L^i (1 - p_L)^{k-i}$$

Under the ideal mappability condition, the minimum N required to achieve a cumulative probability $P(X \le 1)$ below 0.05 is: $P(X \le$

$1) < 0.05 \;\Rightarrow\; 2 \cdot 0.5^N(\binom{N}{0} + \binom{N}{1}) = 2(N+1) \cdot 0.5^N < 0.05 \;\Rightarrow\; N > 8.58$

The solution was retrieved numerically. For $P(X \leq 1) < 0.01$ the solution is $N > 11.25$.

Using these findings, we defined three metrics for evaluating circle detection quality: (1) the proportion of circles with $N \geq 9$, (2) ΔCJ for selected circles, and (3) the proportion of circles fulfilling $p < 0.05$ based on probabilities derived from observed k and N values. For the third metric, we applied the Benjamini-Hochberg correction, designating circles with adjusted probabilities ($p_{adj} < 0.05$) as significantly skewed compared to the expected baseline distribution.

Results

Study design

Given the variability in individual tool performance and the differences between eccDNA and circRNA detection methods, our study evaluated five widely used tools for eccDNA detection—Circle-Map [17], CIRCexplorer2 [40], Circle_finder [41], ecc_finder-bwa, and ecc_finder-minimap2 [42]—, as well as five tools for circRNA detection—CIRCexplorer2, circRNA_finder [43], CIRIquant [19], find_circ [8], and segemehl [44]—. Notably, all tools except ecc_finder-bwa, and ecc_finder-minimap2 are incorporated into nf-core pipelines, which provide standardized, reproducible protocols for detection and analysis, ensuring fair comparisons and minimizing variability due to differences in default parameters or preprocessing. By conducting this combined evaluation, we aimed to systematically identify shared strengths and distinct limitations across these two circular molecule detection approaches.

The study consists of two separate parts making use of *in silico* and biological data. For the *in silico* analysis, we developed *CircleSim* to generate simulated data that approaches biological distributions; thus generating two separate datasets for eccDNA and circRNA with a wide range of coverages from ×5 to ×100. Reads from both *in silico* and biological data were assessed by each tool, reported circles were then filtered based on different criteria (described in Materials and Methods) and the resulting circles were then ordered based on the combinations of tools that reported them.

For *in silico* data, we evaluated a diverse range of metrics for each individual tool including (1) precision, recall, and F-score metrics, (2) variation in the coordinates reported by the tools and (3) deviations in circle detection associated to circle length.

For biological data, we analyzed Circle-Seq and ATAC-seq datasets for eccDNA, and RNA-seq datasets for an original sample—RNase(-)—and the corresponding sample after RNase R treatment—RNase(+)— for circRNA. We evaluated the same downstream filters as in the *in silico* data; as well as the tool combinations. Aside from circle detection patterns, we developed a new metric based on the discordance of reads assigned to the CJ split site (ΔCJ) as a proxy to evaluate the "quality" of circle detection (**Figure 2**).

Circle detection evaluation in *in silico* data

False positive detection is biased toward high coverage

Detection accuracy for eccDNA and circRNA can be significantly affected by sequencing coverage, especially given their low abundance and the specialized methods required to identify them [45]. To investigate this relationship, we evaluated how varying sequencing coverages affect the detection accuracy of eccDNA (**Figure 3A**) and circRNA (**Figure 3B**). We standardized our evaluation by defining a threshold of 20 bp to determine when two circles should be considered identical.

Our initial results indicated that higher sequencing coverage negatively impacted overall detection performance for simulated circles, resulting in lower F-scores at higher coverage levels (**Figure 3A** and **B**, ***unfilter***). This decline primarily reflects a significant drop in precision, despite a modest increase in recall (**Figure S1**, ***unfilter*** and **Supplementary Material 1-2**). In other words, higher coverage allowed for the detection of more true positives (TP) but also substantially increased the number of false positives (FP), ultimately reducing overall accuracy.

To address this FP detection, we evaluated four filtering methods (described in Materials and Methods): (1) *unfilter*, (2) *filter-split*, (3) *filter-duplicates*, and (4) *filter*. Overall, we observed that applying any filter form increases the F-score, showing a plateau at coverages around ×10 to ×20. For good measure, the following analyses were performed using a ×30 coverage (**Table 1**).

Focusing on individual filter comparison, we observed that *filter-split* was generally more effective than *filter-duplicates*, although removing duplicates sometimes enhanced the effectiveness of split read-based filtering (e.g. Circle-Map in eccDNA). Among eccDNA detection tools, CIRCexplorer2 (F-score=0.929), Circle-Map (F-score=0.927), Circle_finder (F-score=0.916), and ecc_finder-minimap2 (F-score=0.896) were the most accurate (**Figure 3A**, ***filter***). For circRNA detection, circRNA_finder (F-score=0.956) and segemehl (F-score=0.974) were identified as the most effective software (**Figure 3B**, ***filter***). However, segemehl was dependent on duplicate removal (F-score=0.973) due to the high number of FPs, which were not adequately addressed by using only split read-based filtering (F-score=0.103). It is worth noting that CIRCexplorer2 output for circRNA simulated data lacked information on reads mapped to the BSJ, which diminished the effectiveness of the split read-based filtering method. Consequently, the filtering approach only managed to filter out overlapping circles and retain longer circles. Surprisingly, find_circ (F-score=0.085) and CIRIquant (F-score=0.035) remained extremely inaccurate even after filtering. This effect is again driven by low recall values, although precision also dropped for find_circ with higher coverages; indicating that these two methods are prone to FP detection (**Figure S1** and **Supplementary Material 1-2**)

Therefore, these results show that high coverages may be unnecessary, if not detrimental for circle detection, incrementing the number of FP circles.

High coverage may affect the offset of circular junction detection

Accurate CJ—DNA breakpoint for eccDNA and BSJ for circRNA—coordinates are necessary for proper circle identification. Stemming from our hypothesis that circular coordinate accuracy may be affected by coverage, we analyzed how stable circle identification was with different coverages. To do this, we calculated the ratio of circles detected with an offset of 1 to the total number of detected circles using *filter*. Ratios approaching 1 indicate that most circles are accurately captured with little-to-no offset, while lower ratios indicate that higher offsets are required, and therefore circle detection is less accurate. Values of these ratios are depicted in **Figures 3C** and **D** for eccDNA and circRNA, respectively.

In eccDNA we observed that for CIRCexplorer2, Circle-Map and Circle_finder the ratio was not affected by coverage, and remained at around 0.9. On the other hand, the ratio decreased with coverage for ecc_finder-minimap2, indicating that, although more circles were detected, even if those circles were "correct" based on the F-score, they were detected with a higher offset from the coordinate of the simulated circle, indicating that CJ sequence was affected by some tool-related factor. Lastly, ecc_finder-bwa showed a "stabilization" of the ratio at around 0.4 with increasing coverage, but the ratio is still low.

Regarding circRNA, two ratio trends arise. On the one hand, tools with low overall detection (find_circ and CIRIquant) show reduced ratios; whereas tools with high accuracy (segemehl and circRNA_finder) show higher ratios above 0.8. Interestingly, CIRCexplorer2, which showed a decrease in circle detection accuracy with increased coverage, shows also a decreased ratio.

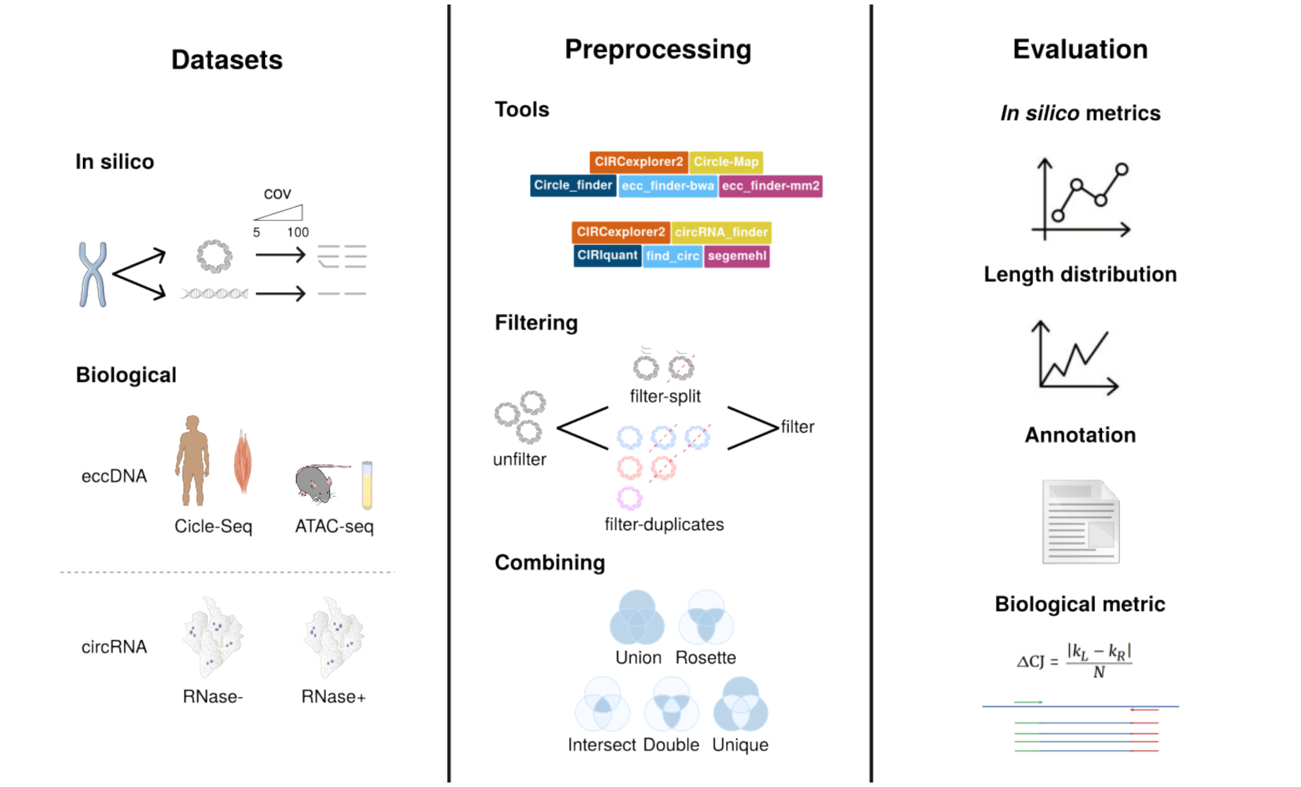

Figure 2. Overview of the study design and analysis workflow. The *in silico* datasets were used to evaluate detection performance, length distribution, and repeat and genomic element enrichment. Biological datasets, including Circle-Seq and ATAC-seq for eccDNA and RNA-seq (RNase−/RNase+) for circRNA, were analyzed using the same filtering and combination strategies. A novel ΔCJ metric was developed to assess the consistency of CJ reads and overall circle detection quality. Icons were taken from Bioicons under CC-BY 3.0 Unported license and NIH Bioart.

Table 1. F-score values at coverage ×30 for eccDNA and circRNA detection tools under the different filtering conditions.

	Tool	*unfilter*	*filter-split*	*filter-duplicates*	*filter*
eccDNA	CIRCexplorer2	0.819	**0.929**	0.882	**0.929**
	Circle-Map	0.618	0.902	0.746	**0.927**
	Circle_finder	0.652	0.903	0.924	**0.916**
	ecc_finder-bwa	0.168	0.096	**0.168**	0.096
	ecc_finder-minimap2	0.916	0.896	**0.916**	0.896
circRNA	CIRCexplorer2	0.856	**0.856**	0.829	0.829
	circRNA_finder	0.867	**0.957**	0.939	0.956
	CIRIquant	0.035	0.035	0.035	0.035
	find_circ	0.056	0.084	0.057	**0.085**
	segemehl	0.094	0.103	0.973	**0.974**

Therefore, it is clear for both eccDNA and circRNA that tools with low F-scores tend to show low ratios showcasing that for tools with higher FP detection values, detected circles are also inaccurately detected, with higher coordinate offsets than their counterparts.

Specific tools showed a circle length anomaly for short circles

After observing that circle detection accuracy was variable across tools, we were interested in testing biases in the detection of circles of specific lengths. Theoretically, since *in silico* circles are generated at random positions in the genome, circle length should not pose a bias in their detection. Expectedly, tools with good circle detection accuracy showed length distributions similar to the expected distribution; whereas for tools with lower accuracies the detection is equally reduced across length (**Figures 4** and **S2**, **left**). This effect was present both for eccDNA (ecc_finder-bwa, KS test, KS=0.55 and p=3.97·10^{-3}) and circRNA (CIRIquant, KS=0.75 and p=9.55·10^{-6}; find_circ, KS=0.60 and p=1.12·10^{-3}).

Considering that the original circle distribution is condensed in shorter circles, we performed an additional analysis on short circles, ranging from 175 to 1,000 bp. Interestingly, a distinct reduction of detected circles occurred in the range of 320 to 480 bp for eccDNA (**Figures 4A** and **S2A**, **right**) and 240 to 480 bp for circRNA (**Figures 4B** and **S2B**, **right**). Although this reduction was more apparent for tools that already show a low circle detection, some tools with good performance showed much lower than expected counts near the 400bp mark in eccDNA Circle_finder (KS=0.38, and p=0.66) and ecc_finde-minimap2 (KS=0.75, and p=1.87·10^{-2}) in eccDNA; and CIRCexplorer2 (KS=0.75, and p=1.87·10^{-2}) and circRNA_finder (KS=0.50, and p=0.283) in circRNA (**Supplementary Material 3**).

Tools showed a biased detection of specific repeated and genomic elements

The genome is not uniform across its sequence, having areas with specific repetitive sequences that, we hypothesize, may affect the ability to detect circles. We are also interested, specially for circRNAs, if there are notable differences in the detection depending on the genomic elements they originated from.

Across the different genomic regions, the repeat element analysis showed the greatest limitation in the detection of satellite ecDNA. Among the 32 eccDNAs laying in satellite regions, all tools showed a lack of accuracy in detecting these circles (F-score 0.2, **Figure 5A**, **left**). These low scores were mostly driven by a lack of

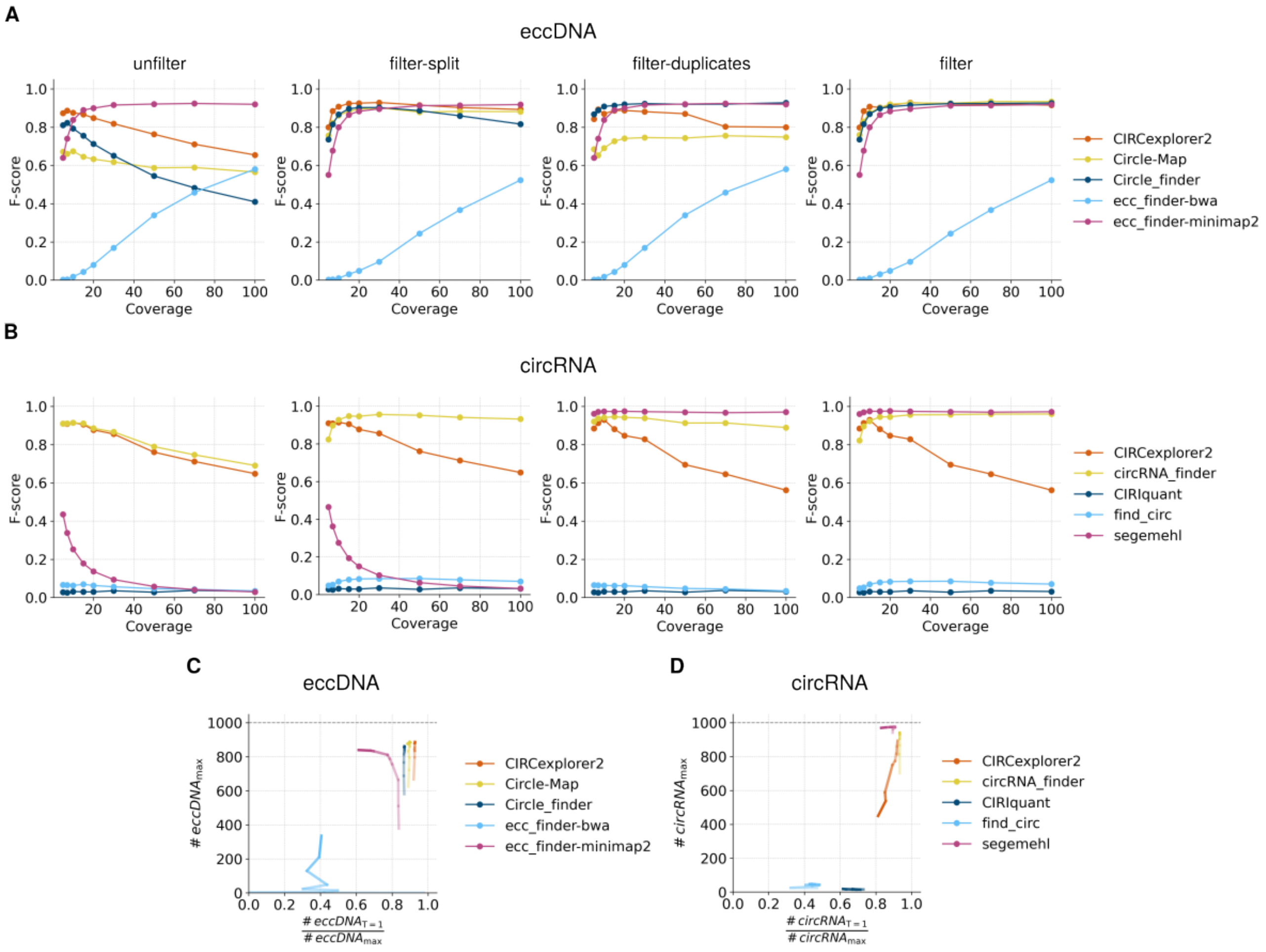

Figure 3. Performance analysis of detection software for eccDNA and circRNA identification in *in silico* datasets. F-score values for (**A**) eccDNA and (**B**) circRNA detection across four filtering conditions: *unfilter*, *filter-split*, *filter-duplicates*, and *filter*. Evaluation of circular predictions at coverage x30 for (**C**) eccDNA and (**D**) circRNA based on the proportion of circles detected with an offset of 1 in the detected coordinate vs real coordinate, relative to the total number of circles detected using *filter* data. Color intensity indicates coverage level, with higher intensity corresponding to greater coverage.

recall, although 3/5 tools showed reduced precision values (0.4 – 0.7) showing that FP circles were also assigned to satellite regions (**Figure S3A**, **left**). For the rest of elements the F-scores were generally high, with moderate decreases in regions without a demarcated repeating element (∅).

There was a marked decrease in accuracy for circles detected in "other"–repetitive elements not marked within the SINE, LINE and similar categories–; however, it is important to note that only two eccDNAs were generated in these regions, meaning that conclusions should be drawn with caution (**Supplementary Material 4**).

In the case of circRNA, no satellite regions were created since they were generated from the transcriptome. Interestingly, CIRCexplorer2 and circRNA_finder exhibited a decrease in their original F-score in SINE regions (**Figures 5B** and **S3B**, **left**; and **Supplementary Material 5**), although this does not happen with eccDNAs.

The analysis of genomic elements showed a considerable decrease in the F-score for eccDNA in intergenic regions (F-score 0.8), due to a low recall. This suggests a significant limitation in the detection of eccDNA in these areas (**Figures 5A** and **S3A**, **right**; and **Supplementary Material 6**). In contrast, circRNA detection showed a decrease in the F-score specifically in the 5'UTR regions. However, it is important to note that, in this case, only two circRNAs were generated in these regions, so the conclusions should be interpreted with caution (**Figures 5B** and **S3B**, **right**; and **Supplementary Material 7**).

Combination of 3 or more tools improved circle detection in in silico data

Although individual tools may perform correctly in specific analyses, we hypothesise that the combination of several tools may improve the accuracy of circle detection, leveraging the strengths and reducing individual tool biases. This is particularly necessary for biological data, where the ground truth is unknown and circle detection may be more difficult to perform due to unknown variation in their sequences.

Although this claim is already supported in the literature, mentioned as *bona fide* circles [26], these circles are usually defined by the combination of two tools. Our aim is to extend it by including three or more tools, under the assumption that the inclusion of more tools may increase the credibility of the detection of common circles. We evaluated the performance of software combinations using five different strategies (described in the Materials and Methods): *Union*, *Rosette*, *Intersect*, *Double*, and *Unique*. Briefly, *Union* is the set of all circles; *Intersect* is of circles detected by all tools while *Unique* refers to circles detected by one tool. *Rosette* is defined as the set of circles detected by two or more tools; whereas *Double* includes circles detected by two or more tools, excluding *Intersect* circles. *Double* circles are used as a comparison to *Rosette*, to see the effect of a more lenient detection of circles. Each strategy is evaluated with 16 tool combinations across the four filtering methods.

Two of the worst combination strategies were *Intersect* and *Unique*, both in eccDNA (**Supplementary Material 8-11**) and cir-

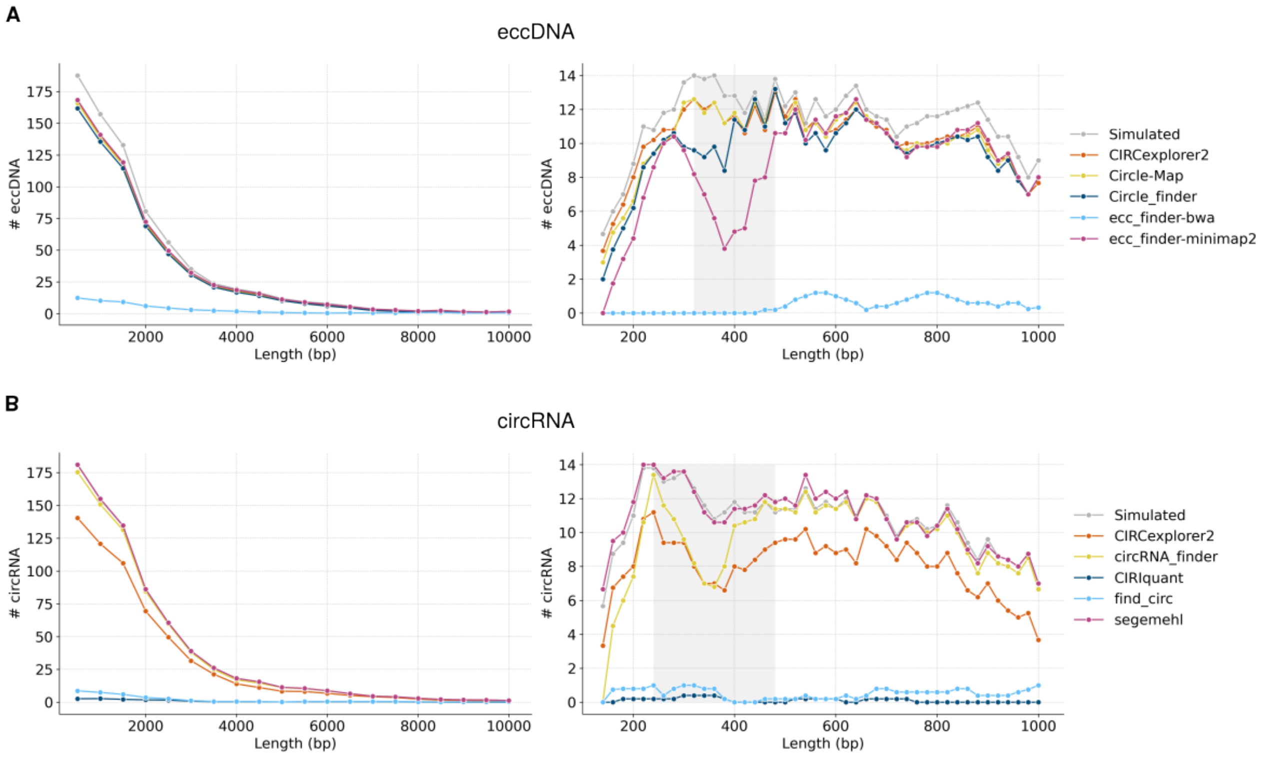

Figure 4. Circular length distribution in *in silico* datasets. Length distribution of detected (**A**) eccDNA and (**B**) circRNA across all size ranges (**left**) and within the short-length range (**right**) in *in silico* datasets at coverage x30. A grey-shaded area highlights the length interval where detection performance was lowest (eccDNA: 320-480 bp; circRNA: 240-480 bp). To enhance the clarity of the distribution plots, a sliding window of size 5 was applied to smooth the distribution curves.

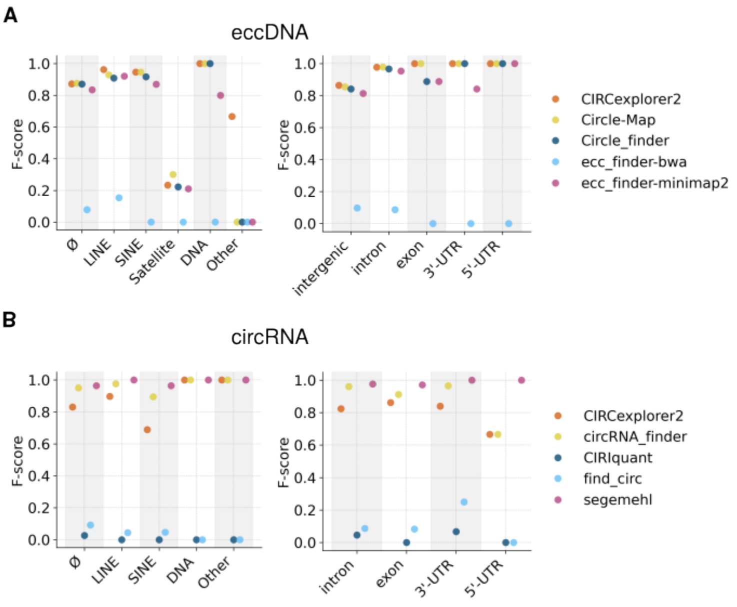

Figure 5. Repeat and genomic element analysis in *in silico* datasets. F-score values of repeat elements (**left**) and genomic features (**right**) associated with detected (**A**) eccDNA and (**B**) circRNA in *in silico* datasets at coverage x30. In circRNAs no intergenic circles were generated and thus the region is not included in this plot.

cRNA (**Supplementary Material 13-16**) (**Figure 6**). The low F-scores were explained by a low precision and recall in *Unique*, and by a low recall but high precision in *Intersect*. *Unique* circles show a low precision and recall values, indicating that simulated (true) circles are not likely to be detected by one tool (low recall) and, also, a *Unique* circle is likely not to be a true circle (low precision).

Nonetheless, for some tool combinations–the best performing ones–*Intersect* yields high precision values. In fact, we see a bimodal distribution for recall, indicating that some tool combinations detect the simulated circles more accurately; although this detection is thus highly dependent on the combination of tools. This effect will be later discussed for biological data.

Regarding *Union* strategy, where all circles detected by all tools are included, it showed high F-scores (>0.8 for most combinations), although their precision values were low, especially for *unfilter* circles. This effect may be driven by the inclusion of *Unique* circles,

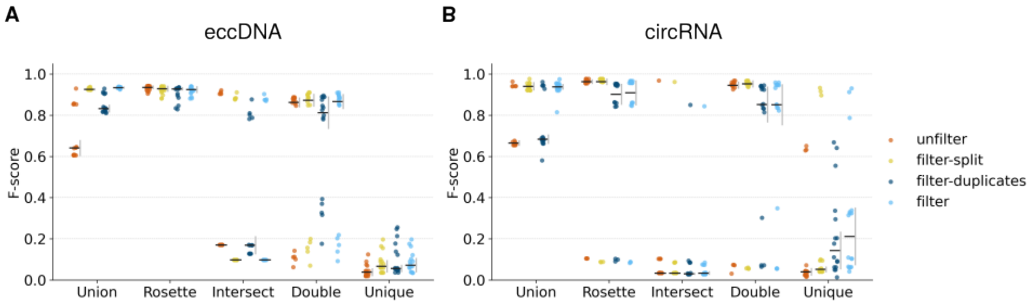

Figure 6. Performance analysis of software combinations for eccDNA and circRNA identification in *in insilico* datasets. Strip plot of the F-score of software combination strategies—*Union*, *Rosette*, *Intersect*, *Double*, and *Unique*—were evaluated under four filtering conditions: *unfilter*, *filter-split*, *filter-duplicates*, and *filter*. F-score values are shown for (**A**) eccDNA and (**B**) circRNA. For each combination-filter pair, the horizontal bar represents the mean value and the vertical gray bar represents the standard deviation.

which already are linked to low F-scores. However, this showcases that a proper filtering may greatly improve the detection quality.

As expected, applying the filtering step led to a slight decrease in recall, which is attributable to an increase in false negatives (FN), and improved precision values, specially for *Union* and *Unique* methods. However, this effect was less pronounced for *filter-duplicates* than for *filter-split*. Thus, F-scores were overall higher in *filter-split* than *filter-duplicates*; and sometimes were similar to *unfilter* or *filter* depending on the combination strategy.

Lastly, the two main strategies to discuss are *Rosette* and *Double*. Both strategies offer a balance between precision and recall. Focusing on eccDNA, *Rosette* yielded higher F-scores than *Double* after *filter-split* (two-tailed Dunn's test, p=0.016) as a result of a lower, although not significant, recall (p=0.253). This difference was visible for the rest of filtering strategies, where *Rosette* showed a similar if not higher recall (**Supplementary Material 12**). For circRNA, *Rosette* and *Double* showed similar results. In fact, both F-scores, precision and recall values were non significant (p=1 for all) (**Supplementary Material 17**).

Overall, these findings highlight that the combination of three or more tools applied by *Rosette* improves detection accuracy for both eccDNA and circRNA. The choice between methods depends on whether maximizing precision, recall, or if a balanced performance is the priority. Additionally, filter option may affect the quality of the detection, with *filter* and *filter-split* showing better results than the rest of filtering options.

Circle detection evaluation in biological data

Analysis of eccDNA and circRNA in biological data were performed with datasets with different processing techniques. For eccDNA, datasets generated using Circle-Seq and ATAC-seq were used; and for circRNA a dataset without RNAse enrichment–RNAse(-)–and with RNAse enrichment–RNAse(+)–were used. Each method has specific particularities affecting the quantity and quality of circle detection that will be further discussed.

Combined tool usage and filter-split improved eccDNA and circRNA detection

To better understand the detection dynamics among circular detection tools, filtering strategies, and circle enrichment techniques, **Figures S5-S8** show, for each method, the number of circles detected by each combination of tools (**Supplementary Material 18**).

In eccDNA, we observed higher detection rates using ATAC-seq compared to Circle-Seq. For instance, for the *unfilter* case, ATAC-seq detected 67,930 circles (5528.60 circles per million of reads) whereas Circle-Seq detected 15,498 circles (1208.04 circles per million of reads). However, Circle-Seq exhibited a higher proportion of circles retained by multiple tools–28.0% (689/1,474) retained when filtered for detection by three or more tools, compared to 8.94% (2,032/22,713) in ATAC-seq–. Although the total amount remains higher in ATAC-seq, Circle-Seq proportionally showed more inner consistence in circle detection.

Furthermore, the number of circles detected by four out of five tools is extremely low in ATAC-seq (3/67,390 = 0.004% unfiltered circles), while Circle-Seq had a significantly higher ratio (642/15,489 = 4.14%) (χ^2 test with Yates correction, χ^2 = 2790.67, p < 0.0001). These observations suggest that Circle-seq captures circles with more robust detection across tools, which potentially indicates a higher-quality capture.

Regarding filtering strategies, both for ATAC-seq and Circle-Seq, *filter-split* retained fewer circles compared to *filter-duplicates* (36.7% vs. 74.2% for ATAC-seq and 23.02% vs. 31.6% for Circle-Seq). Nevertheless, the proportion of circles detected by three or more tools is considerably higher using *filter-split* (12.9% vs. 3.07% in ATAC-seq and 32.0% vs. 5.62% in Circle-Seq). Assuming that circles detected by multiple tools represent higher confidence detections, these results suggest that *filter-split* selects circles of higher confidence compared to *filter-duplicates*.

In terms of tool-specific performance for ATAC-seq, we identified tool groups with high joint detection (**Figure S5**): Circle_finder combined with CIRCexplorer2, Circle-Map individually, and ecc_finder-minimap2 combined with ecc_finder-bwa. These groups persist regardless of the filtering strategy, although circles detected exclusively by Circle-Map decrease when applying *filter-duplicates* and *filter*. In Circle-Seq, the groups ecc_finder-minimap2 combined with ecc_finder-bwa, Circle_finder combined with CIRCexplorer2, and CIRCexplorer2 individually appear (**Figure S6**). However, only ecc_finder-minimap2 combined with ecc_finder-bwa remains robust across different filters. This indicates clear similarities in circle detection among specific tool groups.

Similar dynamics were observed in circRNA detection. Initially, RNase(+) reported a higher number of detected circles compared to RNase(-). For the *unfilter* case, in RNAse(+) 51,909 circles were detected (2208.42 circles per million of reads) whereas in RNase(-) 15,653 circles were detected (438.64 circles per million of reads). However, a lower proportion of circles subsequently passed the filters (e.g. *filter-split*: 11,139 (21.4%) for RNase(+) vs. 6,901 (44.1%) for RNase(-)). This suggests that RNAse enrichment might increase sensitivity, requiring a more stringent filtering.

Furthermore, the filtering effect observed for circRNA mirrors that observed for eccDNA, with *filter-split* yielding a slightly higher percentage of consensus circles compared to *filter-duplicates* (RNase(+): 35.6% (3,969/11,139) vs. 22.8% (7,327/32,110); RNase(-): 30.5% (2,106/6,901) vs. 25.3% (2,392/9,469)). This reaffirms that, when choosing one strategy, *filter-split* is the superior filtering strategy. However, applying both filtering strategies yields a similar net number of circles (RNase(+) 5,538/51,909 (8.1%) vs. RNase(-): 4,620/15,653 (25.5%)), indicating that a two-filtering system, if

Table 2. ΔCJ and p_{adj} ΔCJ values of 3 circle detection examples.

Circle	Condition	Reads (L|R)	ΔCJ	p_{adj} ΔCJ
chr6:39,754,059–39,754,823	TP	22 | 22	0	1
chr6:39,754,063–39,754,823	FP	22 | 22	0	1
chr3:140,171,091–140,172,928	TP	28 | 27	0.029	0.110
chr3:140,171,091–140,174,822	FP	28 | 0	1	$1.3 \cdot 10^{-6}$
chr17:6,795,106–6,797,132	TP	25 | 29	0.072	1
chr17:6,794,297–6,797,033	FP	6 | 39	0.734	$2.7 \cdot 10^{-6}$

possible, may stabilise the number of circles.

ΔCJ is a proxy measure of circle detection quality

One of the limitations of biological data in this context is that no ground truth is available, and thus alternative metrics must be employed to assess detection performance. In this study, we propose the difference in read coverage across each nucleotide of the CJ (ΔCJ) as a proxy for precision. The rationale behind this metric is that, assuming that reads mapped to the CJ are detected on the left side of the CJ at the same rate as on the right side of the CJ, any imbalance in the detection (e.g. a circle with 2 reads assigned on the left side and 28 on the right side) is likely to be a result of an incorrect circle detection; for which a better suited circle may or may not be available. To further improve the robustness of this metric, ΔCJ was refined to account for sequence mappability and read-level alignment quality (described in Materials and Methods).

To illustrate the rationale and behavior of the refined metric, in **Figure S9** we depict three cases based on *in silico* data where each circle was incorrectly detected along with its corresponding TP circle. Circle A represents a case of an exact duplication, where both circles share identical coordinates, resulting in equal read support on both sides of the junction and consequently identical ΔCJ = 0 and p_{adj} = 1. Circles B and C, in contrast, illustrate partial duplications, where one end of the detected circle does not match the true simulated coordinates. In circle B, the undetected right-side junction region exhibits a lower mappability score (0.470) compared to the mapped region (1), which explains the complete lack of reads on the right side. In circle C, both sides of the junction have high mappability (1.0), but the alignment quality of reads supporting the left side is substantially lower (42.793) compared to the right side (60), leading to a reduced effective read count after MAPQ weighting. Thus, these examples illustrate how the ΔCJ_{adj} distinguishes true from false circles (**Table 2**).

Assuming, for each circle with k_L reads assigned to the left, and k_R reads assigned to the right, we can compute the probability p of this configuration, assuming a binomial $B(n = k_L + k_R, p = 0.5)$ distribution. Thus we can set a circle with a high imbalance (0 or 1 reads assigned on one side) as an incorrect circle with $\alpha = 0.05$ if it contains 9 or more reads (described in Materials and Methods). Based on this, we will compute a series of metrics: (1) proportion of circles with $\geq$9 reads, (2) mean and median ΔCJ and (3) ratio of circles with $p_{adj} < 0.05$, where probabilities are adjusted by the Benjamini-Hoechberg method.

To ensure that the metric is a correct proxy for the accuracy of circle detection, we applied it to *in silico* data. To that end, in **Figures S10A** and **B** we show the ΔCJ distribution of TP reads and FP reads for eccDNA and circRNA, respectively. Overall, simulated circles follow a right-tailed distribution of ΔCJ, with median values smaller than 0.1 for both eccDNA and circRNA, showing that most circles have a balanced left/right read distribution. The ΔCJ distributions arising after filtering showed no statistically significant differences, neither for eccDNA (Kruskal-Wallis (KW) test, H=8.78, p_{adj}=0.067) nor for circRNA (H=0.01, p_{adj}=0.999).

In eccDNA, the unfiltered FP circles showed a wider ΔCJ distribution, with a mean value of 0.367, and many of them with ΔCJ=1 (75th percentile is 1). Similarly, circles that still remain FP after filtering showed a high median ΔCJ (*filter*, 0.810), even higher than the unfiltered circles. To better understand the individual filtering effect, circles remaining FP after *filter-split* showed a lower ΔCJ than circles remaining after *filter-duplicates*. This may indicate that *filter-split* removes high-ΔCJ circles, whereas *filter-duplicates* removes low-ΔCJ circles. This effect is also observable based on their assignment probability: there is a higher proportion of circles retained after *filter-duplicates* that have $p_{adj} < 0.05$ (94%), indicating that most of these are unbalanced, in contrast to the proportion of circles retained after *filter-split* (26%) (**Supplementary Material 19**). Thus, most of these low-ΔCJ circles are, expectedly, duplications of simulated circles, and thus may contain a balanced left/right-assigned reads ratio. On the other hand, high-ΔCJ circles may represent new circles, which escape duplicate-filtering, and which contain an imbalanced left-right read ratio, as explained before. These circles are likely to arise as an artifact during the alignment step, where reads are assigned incorrectly to other genomic areas in the side with lowest read presence.

Interestingly, the results on circRNA are different. All circles retained as FP after filtering remain with a low ΔCJ; with the exception of *filter*, which has a similar ΔCJ distribution as in eccDNA. These distributions are likely because most of these circles may be assigned uniquely by segemehl, which contains a extremely high amount of duplicated circles, most of which may be even remain after *filter-duplicates*. Therefore, the remaining circles contain a low ΔCJ. Interestingly, the ratio of FP circles with $p_{adj} < 0.05$ is higher after filter-split (44%) than after *filter-duplicates* (12%) indicating that many of the removed circles by *filter-duplicates* probably had a biased left/right read ratio (**Supplementary Material 20**).

Regional mappability and read mapping quality influences the detectability of circular molecules

Given that read alignment accuracy depends on regional mappability, we examined the extent to which low-mappability regions and MAPQ contribute to the non-detection or incorrect of circular molecules. Beyond the general effect of mappability on detectability, a detailed inspection of TP, FP, and FN shows that these categories differ more from one another than across tools (**FiguresS13** and **S14**).

Focusing first on eccDNA, TP events consistently occur in regions of high mappability and are supported by reads with high MAPQ, reflecting confident and unambiguous alignment. In contrast, there is a considerable proportion of FN (undetectable circles) containing at least one CJ segment in a low-mappability region, which results in poorly aligned or completely absent reads, as evidenced by their minimal MAPQ values. Among the 1000 simulated eccDNAs, 78 were not detected by any tool; of these, 61 contained at least one region lacking a mappability value and 58 had no mapped reads. Similarly, in circRNA, 11 circles were undetectable, 7 of which showed at least one region without a mappability value, and 6 had no mapped reads.

FP predictions, although arising from regions with slightly lower mappability than TP, are most strongly characterized by reads with very low MAPQ. Thus, observing low MAPQ as a shared component of FP and FN circles suggests that alignment uncertainty rather than regional mappability itself is the primary driver of false detections.

Of note, mappability and MAPQ distributions show a clear bi-

modal distribution in the violin plots of **Figures S13** and **S14**, which flattens into a unimodal distribution with a lower tail for distributions with increased circle number. This bimodal distribution may indicate that FN and FP detection is governed by two factors: on the one hand, circles with a decreased mappability and/or MAPQ, which causally affect read alignment and therefore also affect circle detection; on the other hand, circles with high mappability and MAPQ values which are nonetheless incorrectly detected. Since the proportion of these circles is tool-dependent and increases with the number of falsely detected circles, this set of circles may be affected by tool-dependent factors, and thus are not influenced by MAPQ or mappability.

Differences between tools are comparatively small: for instance, CIRCexplorer2 in eccDNA generates FP supported by extremely low-MAPQ reads, whereas ecc_finder-minimap2 shows minimal sensitivity to regional mappability. Overall, detection accuracy is governed more by the intrinsic properties of the circles (TP, FP, FN) than by tool-specific behavior, underscoring the central roles of alignment quality and genomic mappability in both eccDNA and circRNA detection (**Figures S11** and **S12**).

Focusing on circRNAs, we observe that the overall distributions of mappability and MAPQ values both for FP and FN circles is more biased towards values of 1, which indicates a much lower proportion of falsely detected circles explained by low mappability and/or MAPQ. This showcases that the differences in the biogenesis between circRNAs and eccDNAs are translated in tool-specific detection effect. In fact, the increased tendency of FP and FN in circRNAs compared to eccDNA may indicate that circle detection tools may be hindered by the more complex biological properties of the former circles.

Circle capture method greatly affected detection quality in biological data

After establishing ΔCJ as a proxy measure to evaluate the fit of the circular detection, we wanted to observe the variations in ΔCJ for the different circle capture methods in biological data. **Figure 7** shows ΔCJ values for all 4 circle capture methods in each circle detection tool and the four filtering methods.

Regarding eccDNA detection tools, similarities between individual tools are visible again. For instance, ecc_finder-bwa and ecc_finder-minimap2 showed a similar detection pattern based on the ΔCJ for both Circle-Seq and ATAC-seq, and shows overall uniformly increased ΔCJ values at 0.3 (range 0.272-0.333) in ATAC-seq. In Circle-Seq, the filtering effect on the ΔCJ is more apparent than for ATAC-seq. Generally, there are more circles and with lower ΔCJ values remaining after *filter-split*, compared to *filter-duplicates*. ΔCJ values for *filter* are similar, if not lower, to *filter-split*, suggesting again that these two filtering strategies are optimal also for biological datasets.

Additionally, ATAC-seq has a tendency to capture more circles than Circle-Seq, as mentioned before. Interestingly, in tools and filter combinations where the circle number is similar or higher, ΔCJ are higher in ATAC-seq; with few exceptions like *filter-duplicates* in Circle_finder and CIRCexplorer2. Also, ΔCJ for ecc_finder tools are markedly higher in ATAC-seq compared to Circle-Seq. Therefore, it is likely, as mentioned in the previous section, that the higher number of circles captured by ATAC-seq may show a negative correlation with circle quality.

Focusing on circRNA, there is a clear difference in the distribution of ΔCJs: RNase(-) has a quite stable ΔCJ at around 0.4 (differences in *filter*, KW test: H = 10.46, p=0.033); mostly driven by segemehl; while effects across filters are more pronounced in RNase(+) (differences in *filter*, KW test: H = 45.758, $p=2.8 \cdot 10^{-9}$), which shows a clearly reduced ΔCJ compared to RNAse(-). In this case too, compared with eccDNA, a lower number of detected circles correlates with the ΔCJ value (e.g. with different filters) indicating that the two-filtering process tends to retain circles with lower ΔCJ. Additionally, and similarly to eccDNA, circles remaining after *filter-split* show lower ΔCJs than after *filter-duplicates*. Therefore, these results suggest that RNase treatment enhances detection accuracy (**Supplementary Material 21, Supplementary Material 22**).

Rosette tool combination yields the best trade-off between detection accuracy and number of circles in biological data

The final aspect of this analysis is centered on evaluating tool combinations. Firstly, we assessed how ΔCJ varies depending on the combination of tools. To this end, **Figure S13** shows the ΔCJ values for each combination (**Supplementary Material 23**), while **Figure 8** illustrates the difference in ΔCJ between the *Rosette* combination and all other combinations (ΔΔCJ).

From this analysis, although results vary considerably based on the filter and circle enrichment method, we observed a general trend: the rest of tool combination strategies consistently yielded ΔΔCJ < 0, meaning that the *Rosette* combination typically has a lower ΔCJ compared to other combinations. This trend is particularly evident when comparing *Rosette* with *Unique*, where ΔΔCJ is the most pronounced. Regarding filtering, as previously discussed, the lowest ΔCJ values are generally achieved with the *filter* strategy, followed by *filter-split*.

The only exception exhibiting ΔΔCJ > 0 occurred with the *Intersect* combination. This result is expected, as *Intersect* exclusively selects circles detected consistently by all tools. However, *Intersect* detected about half of the circles compared to *Rosette* (**Figure S14**), suggesting that the *Rosette* combination might provide a better balance between the number of detected circles and detection quality.

The ΔCJ values observed between *Rosette* and *Double* combinations were quite similar, with *Rosette* generally having slightly lower ΔCJ values. Nevertheless, this relationship depends on the applied filter (e.g., in RNase(+) ΔΔCJ varies from -0.002 to -0.043). This similarity in ΔCJ between *Double* and *Rosette* is to some extent surprising, considering that *Double* excludes *Intersect* circles. This showcases that the weight of *Rosette* ΔCJ comes from double circles instead of *Intersect* ones.

Finally, we investigated how the number of tools affects ΔCJ. In **Figure S15** ΔCJ is plotted against the number of tools in each tool combination, circle enrichment technique, and filter. Generally, although particularly for *Rosette*, a higher number of tools slightly increased ΔCJ. In certain cases (especially for *Double*), combinations involving more tools produce ΔCJ values midway between individual combinations.

Our interpretation of this phenomenon is that adding more tools could introduce biases from tools such as segemehl or ecc_finder, which, as previously observed, may have lower detection accuracy. Thus ΔCJ values are higher, thereby slightly increasing the combined circles' ΔCJ. However, this increase in ΔCJ from including additional tools is considerably smaller than the effect introduced by the filtering method or circle enrichment technique.

We observed the opposite effect with *Intersect*, where the addition of more tools skewed the ΔCJ distribution towards lower values. As previously explained, this occurs because *Intersect* retains only circles consistently detected across multiple tools, inherently selecting those with greater detection reliability and thus lower ΔCJ, at a cost of a lowered number of circles.

Discussion

The accurate identification of eccDNA and circRNA is essential for understanding their biogenesis, functions, and implications in diseases. These circular molecules play significant roles in gene regulation and are increasingly recognized for their involvement in various pathological conditions, including cancer and neurodegenerative disorders, highlighting their potential use in disease diagnosis and monitoring [9, 10, 11, 46]. However, the lack of standardized evaluation frameworks and consensus protocols for their detec-

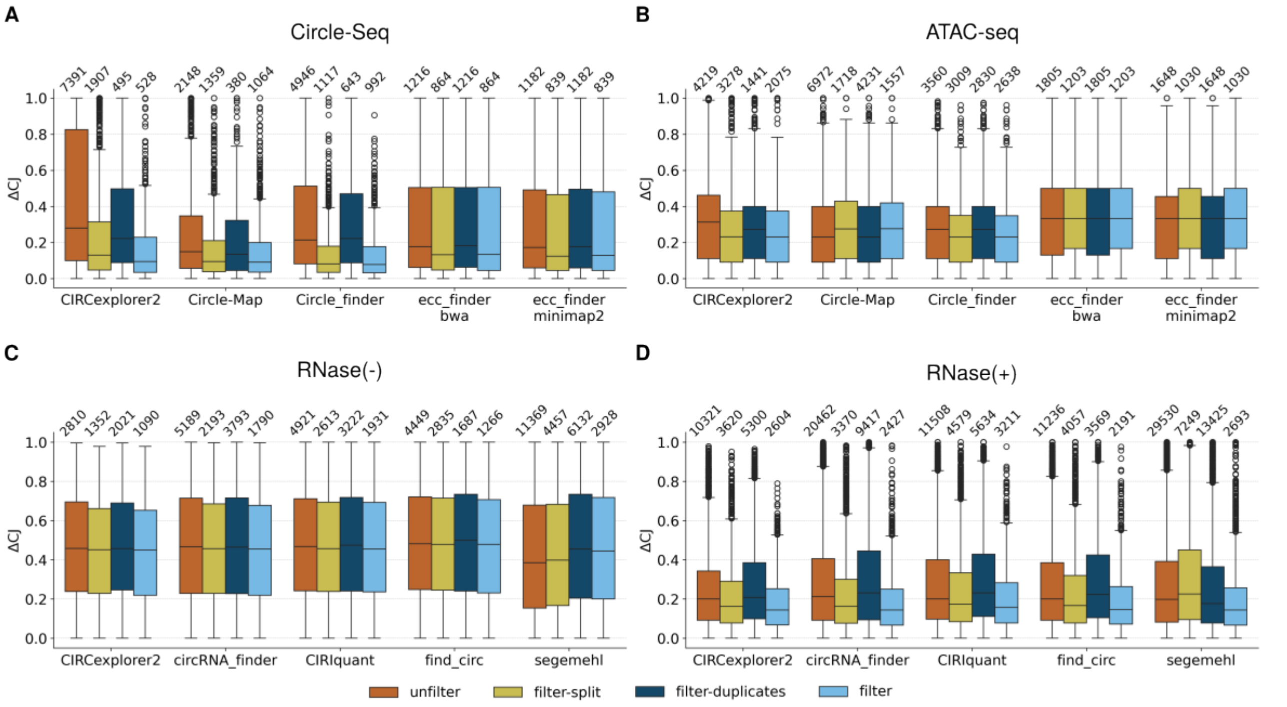

Figure 7. Performance analysis of detection software for eccDNA and circRNA identification in biological datasets. Boxplots of ΔCJ values for (**A**) Circle-Seq and (**B**) ATAC-seq data for eccDNA, and (**C**) RNase(-) and (**D**) RNase(+) data for circRNA, under four filtering conditions: *unfilter*, *filter-split*, *filter-duplicates*, and *filter*.

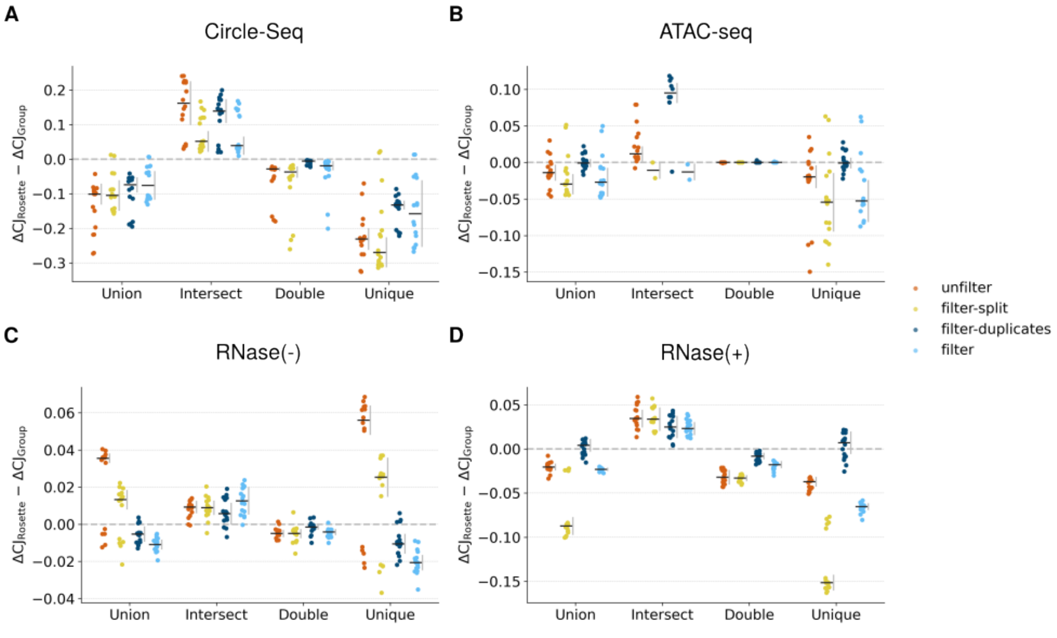

Figure 8. Performance analysis of software combinations for eccDNA and circRNA identification in biological data. Strip plot of ΔΔCJ values ($\Delta CJ_{Rosette}$ - ΔCJ_{Group}) for different tool combinations—*Union*, *Rosette*, *Intersect*, *Double*, and *Unique*—evaluated on biological data, with comparisons made against the *Rosette* combination. Results are shown for (**A**) Circle-Seq and (**B**) ATAC-seq data for eccDNA, and (**C**) RNase(-) and (**D**) RNase(+) data for circRNA, under four filtering conditions: *unfilter*, *filter-split*, *filter-duplicates*, and *filter*.

tion poses a significant challenge, often leading to inconsistencies across studies. To address this issue, we conducted a systematic benchmarking of existing detection methods using both *in silico* and biological datasets, aiming to clarify their strengths, limitations, and practical applicability.

Detection performance varied significantly depending on the type of circular molecule analyzed. Regarding eccDNA, we observed that ecc_finder-minimap2 and CIRCexplorer showed robust results, followed by Circle-Map and Circle_finder. However, applying any filtering strategy led to similar outcomes across all tools. These observations are consistent with findings reported by Li et al. [21] and Gao et al. [20], who also identified Circle-Map and Circle_finder as top-performing tools. Furthermore, Gao et al. [20] reported poor performance of ecc_finder-bwa, aligning with our results and indicating partial reproducibility between studies.

In the case of circRNAs, the comprehensive analysis by Zeng

et al. [22], although not entirely replicable in our study, suggests a correlation between the number of circles detected and the F1 score for each tool. Conversely, Vromman et al. [23] observed a clear detection bias, finding segemehl among the tools with lower detection rates, contrasting sharply with our results. This discrepancy could arise from several factors, including the fact that segemehl, initially designed as an aligner, was later integrated into circRNA analysis workflows; consequently, its performance might be highly dataset-dependent. Additionally, Liu et al. [47] analyzed the effect of coverage similar to our study, although they did not observe significant impacts of coverage on detection, possibly due to their analysis not including higher coverage levels like ours, where we indeed observed an increase in FPs. They also reported better performance for CIRI and circRNA_finder compared to our findings, potentially explained by their use of CIRI-simulator [38] for circle simulation, which may inherently bias results in favor of these tools.

Focusing on biological datasets, we found the library preparation method to be one of the most influential factors for both eccDNA and circRNA detection (ATAC-seq vs. Circle-seq for eccDNA, and RNase+ vs. RNase- for circRNA). For eccDNA, we detected a greater number of circles using ATAC-seq compared to Circle-seq, conflicting with results reported by Gao et al. [20], who observed significantly higher detection rates (up to two orders of magnitude) for Circle-seq. This discrepancy might be attributed to sample origin or other internal methodological factors, though the exact reason remains unclear.

In contrast, our circRNA findings align well with studies such as Zeng et al. [22], who reported increased circle detection following RNase treatment (approximately 1.5 to 3 times higher). Nonetheless, we observed that circle detection numbers strongly depend on the tools employed. One potential confounding factor influencing this variability is the alignment algorithm, which differs among tools. To minimize this variability, we standardized the aligners used as much as possible—employing STAR for circRNA and bwa for eccDNA detection. However, disparities in results still indicated significant aligner-specific factors influencing circle detection accuracy.

We also conclude that targeted amplification methods notably affect detection outcomes, potentially introducing additional biases. For example, Rolling Circle Amplification (RCA), used in Circle-seq, can lead to either overrepresentation or underrepresentation of circles [48]. Moreover, such amplification methods may impede the analysis of epigenetic signals, which are linked to gene expression variations and regulatory alterations. Consequently, non-targeted sequencing approaches such as ATAC-seq and RNA-seq are gaining interest, although further investigation is necessary to fully understand and address potential biases inherent in these methods. Additionally, the analysis derived from our results suggest a preference of use of targeted methods such as Circle-seq compared to untargated methods like ATAC-seq.

Delving deeper into the analysis, several structural effects deserve discussion, particularly the issue of FPs. Initially, we observed that coverage substantially influences FP detection in *in silico* data, a result whose applicability to biological data remains unclear but warrants consideration in analyses.

We aimed to understand the origin of these FPs. Zeng et al. [22] defined FPs as circles detected in RNase(-) treatment but not in RNase(+), which theoretically amplifies the circular RNA signal. Our findings highlight the need for further exploration of FP origins, particularly in *in silico* datasets, to enhance their applicability in biological studies. We propose that FPs may originate from two main sources: computational and biological effects. Computational effects include coverage-related issues, where increased sequence detection near the CJ may occasionally lead to incorrect identification due to partial flanking or sequencing errors, creating artificial circles. Additional computational factors encompass sequence length, the aligner used, and the detection algorithm itself. Biological effects primarily involve amplification methods—techniques excluding linear DNA or RNA can reduce FPs by preventing amplification of sequences resembling CJ, which may result from alternative splicing or genomic mutations and rearrangements that do not yield genuine circles. Consequently, methods involving purification steps (such as Circle-Seq for eccDNA and RNase(+) for circRNA) generally provide superior results compared to those lacking this step.

Another crucial factor affecting the analysis and significantly reducing FPs is the appropriate use of filters. Both *in silico* and biological datasets demonstrated a considerable decrease in detected circles upon filtering. Our findings align with Vromman et al. [23], who reported substantial reduction in circles with $\geq$5 reads mapped to the BSJ.

In our analysis, apart from applying a filter based on counts ($\geq$2 reads), we also implemented a duplicate filter. Generally, both filters performed adequately, though the filter-split occasionally yielded slightly superior results. A potential reason is that many tools do not provide precise CJ coordinates; rather, they often detect circles at adjacent coordinates, typically supported by low read counts (1 or 2) and occasionally mismatches that cause partial misalignment. Thus, many spurious circles escape duplicate filtering, making filter-split more effective in such cases.

An additional intriguing observation from our *in silico* analysis is related to circle length. We noted a marked decline in detected circles around 340 bp in both eccDNA and circRNA, possibly corresponding to dinucleosome length. However, since *in silico* data are independent of biological factors, this finding is surprising. It could be attributed to detection biases such as those observed in ecc_finder, which fails to detect shorter eccDNAs (<400 bp), an issue also reported by Li et al. [21]. Nonetheless, this issue would suggests that circle length biases associated to tool usage requires further study.

Lastly, repetitive sequences significantly impact circle detection. Gao et al. [20] observed pronounced effects on detecting reads associated with LTR, SINE, and LINE elements, depending on whether the sequencing technology involved long or short reads, albeit representing a small fraction of total reads (0.1% for short reads). Our *in silico* results showed F-scores slightly above 0.8 for eccDNA and between 0.8 and 1 for circRNA for LINEs and SINEs, suggesting a slightly higher yet potentially problematic detection fraction in biological datasets due to the repetitive nature of these sequences. The most pronounced effect occurred with satellite sequences, where detection accuracy declined sharply. This phenomenon may substantially impact eccDNA detection in centromeric regions, which, due to their repetitive nature, could be inaccurately captured, potentially leading to significant functional loss in analyses. This effect is related to the lack of circle detection or lowered detection accuracy in low mappability regions, that has been shown in our analysis.

A significant limitation of current benchmarking studies is the absence of robust metrics for evaluating accurate circle detection in biological data. Often, comparisons rely on the number of detected circles or similarity between tools, neither of which provide true validation. Alternative methods such as external validations using qPCR or similar techniques cannot scale effectively beyond a few circles.

In this study, we employed the ΔCJ metric, developed under the assumption that reads assigned to the CJ will equally distribute between its left and right sides. Consequently, significant deviations from this symmetry indicate potential incorrect assignments. After confirming the rationale through specific examples and verifying its applicability in *in silico* data, we found that the use of ΔCJ in biological datasets provides valuable insights for evaluating tool effectiveness, purification methods, and filtering strategies.

Additionally, although the results are only suggestive and would require a more in-depth study, the use of ΔCJ has uncovered a new possible mechanism for the generation of FP and FN circles, dependent on the mappability of the genome and the mapping quality.

Mapping quality is generated during the read alignment process and represents the probability of a read being correctly assigned to that specific region. Reads with low read quality may arise from sequencing artifacts or from mapping to a somewhat similar sequence to the read, which may correspond to a secondary region that was propritized during the alignment. Thus, part of FP circles are mistakenly assigned to a region different to the CJ, leading to a low mapping quality alignment. On the other hand, FN circles may arise from a twofold combination of aligning reads to low mappability regions, which produce low quality alignments due to the increased mismatch rate, and therefore are not accurately detected by the circle detection tools. Thus, circular molecules arising from low mappability regions have a higher probability of being underdetected under short read sequencing strategies. Future studies comparing mappability distributions of circles detected under short-read and long-read strategies would expand the insights of the effect of this metric on circle detection.

Therefore, the inclusion of ΔCJ as a surrogate metric for circle detection quality, as well as mapping quality and region mappability metrics to confirm circle detection errors, may be beneficial for studies to improve the quality of reported circles, or to evaluate detection biases, where the higher-than-expected FPs are likely to be mitigated by this metric.

Despite its relevance, it is crucial to recognize the conceptual and practical limitations of ΔCJ. For instance, some FP circles might escape detection due to factors unrelated to symmetry imbalances, limiting the metric to detecting only certain types of FPs. Additionally, the reliability of this metric requires a relatively high number of reads (preferably more than 20-30), restricting its effectiveness for detecting circles with low read counts. One advantage of analysing read unbalances is that high-read FPs with significant asymmetry may potentially be reassigned to circles sharing one CJ coordinate, generating thus a corrected circle list. Lastly, we observed that for circRNAs, the metric is noisier, possibly because many circRNAs have boundaries defined precisely at intron-exon junctions, conflicting with the expected randomness of read distribution. To improve on this limitations a more robust theoretical framework of circle read mapping to CJ that accounts for sequencing depth and circle length may be necessary.

One advantage of incorporating multiple detection tools in analyses is the improvement in eccDNA and circRNA detection, especially considering analysis workflows like those implemented in nf-core, which facilitate integration of multiple tools [28]. This observation aligns with Hansen [26], who identified commonly detected circles as *bona fide* circles. Other studies have similarly highlighted the variability in detection consistency among tools. For instance, Vromman et al. [23] reported that nearly 50% of circles detected were unique to a single tool—though the exact percentages varied considerably by tool—while approximately 10% were consistently detected across ten or more tools. Likewise, Li et al. [21] suggest that including two or more tools can enhance the robustness of circle detection.

Vromman et al. [23] validated circles consistent across multiple tools, observing that employing two or more tools notably reduced FPs. However, while this outcome could theoretically extend to eccDNA, direct validation for eccDNA is lacking, and additional validation measures for various tool combinations are needed.

Despite the *bona fide* circle strategy being reported in previous works, our benchmark is the first suggesting a well-defined and demonstrated tool combination strategy, *Rosette*, that achieves the best balance between the number of circles reported and FP reduction.

The rationale for the *Rosette* strategy is that circles detected by multiple tools are likely to represent true positives, whereas those identified by a single tool should be interpreted cautiously. Among all tested combinations, *Rosette* exhibited superior performance in simulated data by significantly lowering the FP rate while maintaining high sensitivity, even without additional filtering. Similarly, in biological data, it enhanced detection capacity without compromising accuracy. Nevertheless, further validation using deeper analysis of reads associated with detected circles would bolster the reliability of this approach.

Additionally, we observed that the effectiveness of combined detection strategies depends on the individual accuracy of each tool. This aligns with Vromman et al. [23], who found that the FP rate for combined tools approximates an average of the individual FP rates. Thus, the choice of combination strategy (particularly between *Rosette* and *Intersect*) depends on the analytical goal. In clinical settings or environments requiring higher positive predictive value, *Intersect* may be more advantageous despite detecting fewer circles. In contrast, *Rosette* may offer a sufficiently robust and reliable circle set suitable for broader research applications.

Additional files

Supplementary Material 1. Performance analysis of detection software for eccDNA identification in *in silico* datasets.

Supplementary Material 2. Performance analysis of detection software for circRNA identification in *in silico* datasets.

Supplementary Material 3. Kolmogorov-Smirnov test for circular length distribution in *in silico* datasets.

Supplementary Material 4. Repeat element annotation for eccDNA in *in silico* datasets.

Supplementary Material 5. Repeat element annotation for circRNA in *in silico* datasets.

Supplementary Material 6. Genomic element annotation for eccDNA in *in silico* datasets.

Supplementary Material 7. Genomic element annotation for circRNA in *in silico* datasets.

Supplementary Material 8. Performance analysis of software combinations detection for eccDNA in *in silico unfilter* condition datasets.

Supplementary Material 9. Performance analysis of software combinations detection for eccDNA in *in silico filter-split* condition datasets.

Supplementary Material 10. Performance analysis of software combinations detection for eccDNA in *in silico filter-duplicates* condition datasets.

Supplementary Material 11. Performance analysis of software combinations detection for eccDNA in *in silico filter* condition datasets.

Supplementary Material 12. Dunn's test results for software combination detection performance metrics for eccDNA in *in silico* datasets.

Supplementary Material 13. Performance analysis of software combinations detection for circRNA in *in silico unfilter* condition datasets.

Supplementary Material 14. Performance analysis of software combinations detection for circRNA in *in silico filter-split* condition datasets.

Supplementary Material 15. Performance analysis of software combinations detection for circRNA in *in silico filter-duplicates* condition datasets.

Supplementary Material 16. Performance analysis of software combinations detection for circRNA in *in silico filter* condition datasets.

Supplementary Material 17. Dunn's test results for software combination detection performance metrics for circRNA in *in silico* datasets.

Supplementary Material 18. Circular detection in biological datasets.

Supplementary Material 19. ΔCJ evaluation as a proxy measure of circle detection quality for eccDNA identification in *in silico* datasets.

Supplementary Material 20. △CJ evaluation as a proxy measure of circle detection quality for circRNA identification in *in silico* datasets.

Supplementary Material 21. Performance analysis of detection software in biological datasets.

Supplementary Material 22. Dunn's test results for software combination detection performance metrics in biological datasets.

Supplementary Material 23. Performance analysis of detection software combinations in biological datasets.

Supplementary Fig. S1. Performance analysis of detection software for eccDNA and circRNA identification in *in silico* datasets.

Supplementary Fig. S2. Circular length distribution analysis in *in silico* datasets.

Supplementary Fig. S3. Repeat element analysis and genomic element analysis in *in silico* datasets.

Supplementary Fig. S4. Performance analysis of software combinations for eccDNA and circRNA identification in *in silico* datasets.

Supplementary Fig. S5. eccDNA detection in Circle-Seq data.

Supplementary Fig. S6. eccDNA detection in ATAC-seq data.

Supplementary Fig. S7. eccDNA detection in RNase(-) data.

Supplementary Fig. S8. circRNA detection in RNase(+) data.

Supplementary Fig. S9. IGV visualization of three eccDNA false positives in *in silico* datasets.

Supplementary Fig. S10. Circular junction nucleotide difference (△CJ) in *in silico* datasets.

Supplementary Fig. S11. Circular junction mappability in *in silico* datasets.

Supplementary Fig. S12. Circular junction read mapping quality (MAPQ) in *in silico* datasets.

Supplementary Fig. S13. Circular junction nucleotide difference (△CJ) of software combinations for eccDNA and circRNA identification in biological datasets.

Supplementary Fig. S14. Comparation of detected circles between Rosette and Intersect.

Supplementary Fig. S15. Performance of tool combinations for eccDNA and circRNA in biological datasets.

Abbreviations

BSJ: backsplice junction; CJ: circular junctions; circRNA: circular RNA; △CJ: the discrepancy in read assignment to each side of the breakpoint; DNASE1L3: deoxyribonuclease 1 like 3; eccDNA: extrachromosomal circular DNA; FN: false negative; FP: false positive; KS: Kolmogorov-Smirnov test; KW: Kruskal-Wallis test; LINE: Long Interspersed Nuclear Element; LTR: Long Terminal Repeats; MAPQ: mapping quality; NCBI: National Center for Biotechnology Information; pre-mRNA: precursor messenger RNA; SINE: Short Interspersed Nuclear Elements; snRNA: small nuclear RNA; TP: true positive; UTR: untranslated region; WGS: whole-genome sequencing; Ø: non-repetitive region.

Acknowledgements

All circular detection analyses were conducted on the Hyperion cluster at the Donostia International Physics Center (DIPC). The author acknowledges the technical and human support provided by the DIPC Supercomputing Center. The authors have used generative AI technology (ChatGPT) to improve the readability and overall quality of the manuscript. The use of AI has not altered the content or messages conveyed in the manuscript, focusing solely on refining the writing for clarity and enhanced readability.

Author's Contributions

Author contributions are detailed according to the CRediT taxonomy. AZ, AMA, and DO contributed to the conceptualization of the study. Methodology was developed by AZ, AMA, IPL, and DO. Software development and implementation were carried out by AZ and AMA. Validation and formal analysis were performed by AZ and AMA. AZ was responsible for the investigation. Resources were provided by AZ, AMA, and DO. Data curation was handled by AZ and AMA. All authors—AZ, AMA, IPL, and DO—contributed to writing the original draft, as well as to reviewing and editing the manuscript. Visualization was carried out by AZ. Supervision was provided by AMA, IPL, and DO, while project administration was managed by AMA and DO. Funding acquisition was undertaken by DO.

Funding

AZ is supported by a predoctoral fellowship from the Basque Government (PRE_2025_1_0138). AMA is supported by the IKUR-Nanoneuro initiative (Basque Government). IPL is supported by the Dietmar Hopp Foundation. This study (DO) has been funded by Instituto de Salud Carlos III (ISCIII) through the project PI23/00903 and co-founded by the European Union.

Data Availability

- Project name: benchmarking
- Project home page: https://github.com/ZabalaAitor/benchmarking
- Data repository: https://doi.org/10.5281/zenodo.15783793
- Notebook output repository: https://doi.org/10.5281/zenodo.15783795
- Operating system: Platform independent
- Programming language: Python

  The data sets supporting the results of this article are available in the ENA repository, accession number PRJEB95764.

Computational resources

All circular detection analyses were conducted using on a single computing node with 256 GB of RAM and 24 cores.

Competing Interests

The author(s) declare that they have no competing interests.

References

1. Cohen S, Mechali M. A novel cell-free system reveals a mechanism of circular DNA formation from tandem repeats. Nucleic acids research 2001;29(12):2542–2548.
2. Chen LL, Yang L. Regulation of circRNA biogenesis. RNA biology 2015;12(4):381–388.
3. Cohen S, Houben A, Segal D. Extrachromosomal circular DNA derived from tandemly repeated genomic sequences in plants. The Plant Journal 2008;53(6):1027–1034.
4. Møller HD, Parsons L, Jørgensen TS, Botstein D, Regenberg B. Extrachromosomal circular DNA is common in yeast. Proceedings of the National Academy of Sciences 2015;112(24):E3114–E3122.
5. Møller HD, Mohiyuddin M, Prada-Luengo I, Sailani MR, Halling JF, Plomgaard P, et al. Circular DNA elements of chromosomal origin are common in healthy human somatic tissue. Nature communications 2018;9(1):1069.
6. Møller HD, Ramos-Madrigal J, Prada-Luengo I, Gilbert MTP,

Regenberg B. Near-random distribution of chromosome-derived circular DNA in the condensed genome of pigeons and the larger, more repeat-rich human genome. Genome biology and evolution 2020;12(2):3762–3777.

7. Sanger HL, Klotz G, Riesner D, Gross HJ, Kleinschmidt AK. Viroids are single-stranded covalently closed circular RNA molecules existing as highly base-paired rod-like structures. Proceedings of the National Academy of Sciences 1976;73(11):3852–3856.
8. Memczak S, Jens M, Elefsinioti A, Torti F, Krueger J, Rybak A, et al. Circular RNAs are a large class of animal RNAs with regulatory potency. Nature 2013;495(7441):333–338.
9. Turner KM, Deshpande V, Beyter D, Koga T, Rusert J, Lee C, et al. Extrachromosomal oncogene amplification drives tumour evolution and genetic heterogeneity. Nature 2017;543(7643):122–125.
10. Zhang Hd, Jiang Lh, Sun Dw, Hou Jc, Ji Zl. CircRNA: a novel type of biomarker for cancer. Breast cancer 2018;25:1–7.
11. Iparraguirre L, Muñoz-Culla M, Prada-Luengo I, Castillo-Triviño T, Olascoaga J, Otaegui D. Circular RNA profiling reveals that circular RNAs from ANXA2 can be used as new biomarkers for multiple sclerosis. Human molecular genetics 2017;26(18):3564–3572.
12. Jeck WR, Sorrentino JA, Wang K, Slevin MK, Burd CE, Liu J, et al. Circular RNAs are abundant, conserved, and associated with ALU repeats. Rna 2013;19(2):141–157.
13. Møller HD, Bojsen RK, Tachibana C, Parsons L, Botstein D, Regenberg B. Genome-wide purification of extrachromosomal circular DNA from eukaryotic cells. JoVE (Journal of Visualized Experiments) 2016;(110):e54239.
14. Iparraguirre L, Prada-Luengo I, Regenberg B, Otaegui D. To be or not to be: circular RNAs or mRNAs from circular DNAs? Frontiers in Genetics 2019;10:940.
15. Kumar P, Kiran S, Saha S, Su Z, Paulsen T, Chatrath A, et al. ATAC-seq identifies thousands of extrachromosomal circular DNA in cancer and cell lines. Science advances 2020;6(20):eaba2489.
16. Digby B, Finn SP, Ó Broin P. nf-core/circrna: a portable workflow for the quantification, miRNA target prediction and differential expression analysis of circular RNAs. BMC bioinformatics 2023;24(1):27.
17. Prada-Luengo I, Krogh A, Maretty L, Regenberg B. Sensitive detection of circular DNAs at single-nucleotide resolution using guided realignment of partially aligned reads. BMC bioinformatics 2019;20:1–9.
18. Salzman J, Gawad C, Wang PL, Lacayo N, Brown PO. Circular RNAs are the predominant transcript isoform from hundreds of human genes in diverse cell types. PloS one 2012;7(2):e30733.
19. Zhang J, Chen S, Yang J, Zhao F. Accurate quantification of circular RNAs identifies extensive circular isoform switching events. Nature communications 2020;11(1):90.
20. Gao X, Liu K, Luo S, Tang M, Liu N, Jiang C, et al. Comparative analysis of methodologies for detecting extrachromosomal circular DNA. Nature Communications 2024;15(1):9208.
21. Li F, Ming W, Lu W, Wang Y, Dong X, Bai Y. Bioinformatics advances in eccDNA identification and analysis. Oncogene 2024;43(41):3021–3036.
22. Zeng X, Lin W, Guo M, Zou Q. A comprehensive overview and evaluation of circular RNA detection tools. PLoS computational biology 2017;13(6):e1005420.
23. Vromman M, Anckaert J, Bortoluzzi S, Buratin A, Chen CY, Chu Q, et al. Large-scale benchmarking of circRNA detection tools reveals large differences in sensitivity but not in precision. Nature methods 2023;20(8):1159–1169.
24. Digby B, Finn S, Ó Broin P. Computational approaches and challenges in the analysis of circRNA data. BMC genomics 2024;25(1):527.
25. Szabo L, Salzman J. Detecting circular RNAs: bioinformatic and experimental challenges. Nature Reviews Genetics 2016;17(11):679–692.
26. Hansen TB. Improved circRNA identification by combining prediction algorithms. Frontiers in cell and developmental biology 2018;6:330528.
27. Gaffo E, Buratin A, Dal Molin A, Bortoluzzi S. Sensitive, reliable and robust circRNA detection from RNA-seq with CirComPara2. Briefings in Bioinformatics 2021 Oct;23(1). http://dx.doi.org/10.1093/bib/bbab418.
28. Ewels PA, Peltzer A, Fillinger S, Patel H, Alneberg J, Wilm A, et al. The nf-core framework for community-curated bioinformatics pipelines. Nature biotechnology 2020;38(3):276–278.
29. Di Tommaso P, Chatzou M, Floden EW, Barja PP, Palumbo E, Notredame C. Nextflow enables reproducible computational workflows. Nature biotechnology 2017;35(4):316–319.
30. Schreyer D, nf-core bot, Ewels P, Peltzer A, nf-core/circdna: v1.1.0 – Tremendous Wombat. Zenodo; 2024. https://doi.org/10.5281/zenodo.10643212.
31. Zhao Y, Yu L, Zhang S, Su X, Zhou X. Extrachromosomal circular DNA: Current status and future prospects. eLife 2022 Oct;11. http://dx.doi.org/10.7554/eLife.81412.
32. Zhang XO, Wang HB, Zhang Y, Lu X, Chen LL, Yang L. Complementary sequence-mediated exon circularization. Cell 2014;159(1):134–147.
33. Kim D, Pertea G, Trapnell C, Pimentel H, Kelley R, Salzberg SL. TopHat2: accurate alignment of transcriptomes in the presence of insertions, deletions and gene fusions. Genome biology 2013;14:1–13.
34. Dobin A, Davis CA, Schlesinger F, Drenkow J, Zaleski C, Jha S, et al. STAR: ultrafast universal RNA-seq aligner. Bioinformatics 2013;29(1):15–21.
35. Li H, Durbin R. Fast and accurate short read alignment with Burrows–Wheeler transform. bioinformatics 2009;25(14):1754–1760.
36. Li H. Minimap2: pairwise alignment for nucleotide sequences. Bioinformatics 2018;34(18):3094–3100.
37. Sin ST, Deng J, Ji L, Yukawa M, Chan RW, Volpi S, et al. Effects of nucleases on cell-free extrachromosomal circular DNA. JCI insight 2022;7(8).
38. Gao Y, Wang J, Zhao F. CIRI: an efficient and unbiased algorithm for de novo circular RNA identification. Genome biology 2015;16:1–16.
39. Lex A, Gehlenborg N, Strobelt H, Vuillemot R, Pfister H. UpSet: Visualization of Intersecting Sets. IEEE Transactions on Visualization and Computer Graphics 2014;20(12):1983–1992.
40. Zhang XO, Dong R, Zhang Y, Zhang JL, Luo Z, Zhang J, et al. Diverse alternative back-splicing and alternative splicing landscape of circular RNAs. Genome research 2016;26(9):1277–1287.
41. Kumar P, Dillon LW, Shibata Y, Jazaeri AA, Jones DR, Dutta A. Normal and cancerous tissues release extrachromosomal circular DNA (eccDNA) into the circulation. Molecular Cancer Research 2017;15(9):1197–1205.
42. Zhang P, Peng H, Llauro C, Bucher E, Mirouze M. ecc_finder: a robust and accurate tool for detecting extrachromosomal circular DNA from sequencing data. Frontiers in plant science 2021;12:743742.
43. Westholm JO, Miura P, Olson S, Shenker S, Joseph B, Sanfilippo P, et al. Genome-wide analysis of drosophila circular RNAs reveals their structural and sequence properties and age-dependent neural accumulation. Cell reports 2014;9(5):1966–1980.
44. Hoffmann S, Otto C, Kurtz S, Sharma CM, Khaitovich P, Vogel J, et al. Fast mapping of short sequences with mismatches, insertions and deletions using index structures. PLoS computational biology 2009;5(9):e1000502.
45. Tsai SQ, Nguyen NT, Malagon-Lopez J, Topkar VV, Aryee MJ, Joung JK. CIRCLE-seq: a highly sensitive in vitro screen

for genome-wide CRISPR–Cas9 nuclease off-targets. Nature Methods 2017 May;14(6):607–614. http://dx.doi.org/10.1038/nmeth.4278.

46. Noer JB, Hørsdal OK, Xiang X, Luo Y, Regenberg B. Extrachromosomal circular DNA in cancer: history, current knowledge, and methods. Trends in Genetics 2022 Jul;38(7):766–781. http://dx.doi.org/10.1016/j.tig.2022.02.007.
47. Liu H, Akhatayeva Z, Pan C, Liao M, Lan X. Comprehensive comparison of two types of algorithm for circRNA detection from short-read RNA-Seq. Bioinformatics 2022 Apr;38(11):3037–3043. http://dx.doi.org/10.1093/bioinformatics/btac302.
48. Yu J, Zhang H, Han P, Jiang X, Li J, Li B, et al. Circle-seq based method for eccDNA synthesis and its application as a canonical promoter independent vector for robust microRNA overexpression. Computational and Structural Biotechnology Journal 2024 Dec;23:358–368. http://dx.doi.org/10.1016/j.csbj.2023.12.019.

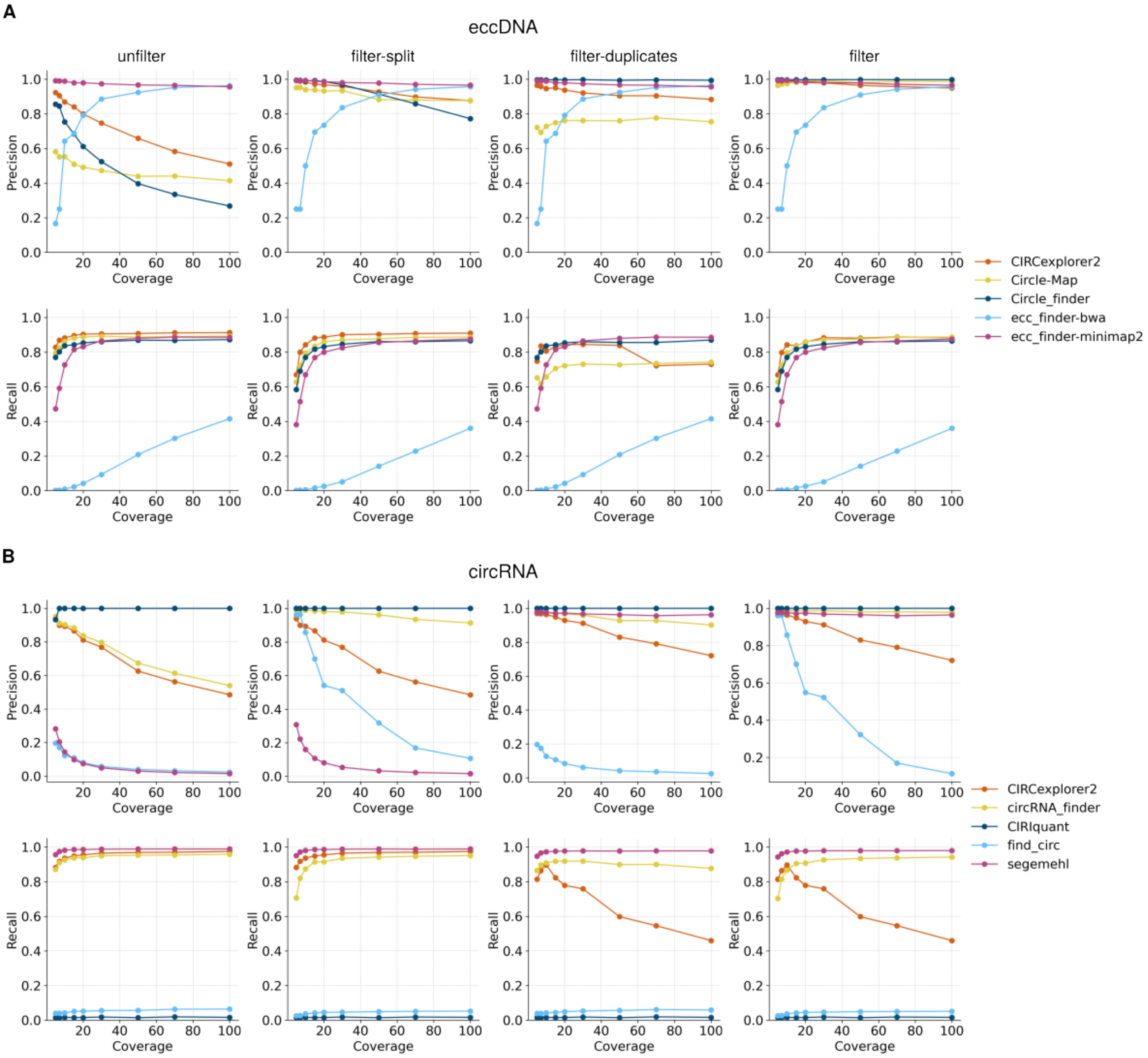

Figure S1. Performance analysis of detection software for eccDNA and circRNA identification in *in silico* datasets. Precision (**above**) and recall (**below**) values for eccDNA (**A**) and circRNA (**B**) detection across four filtering conditions: unfilter, filter-split, filter-duplicates, and filter. Color intensity indicates coverage level, with higher intensity corresponding to greater coverage.

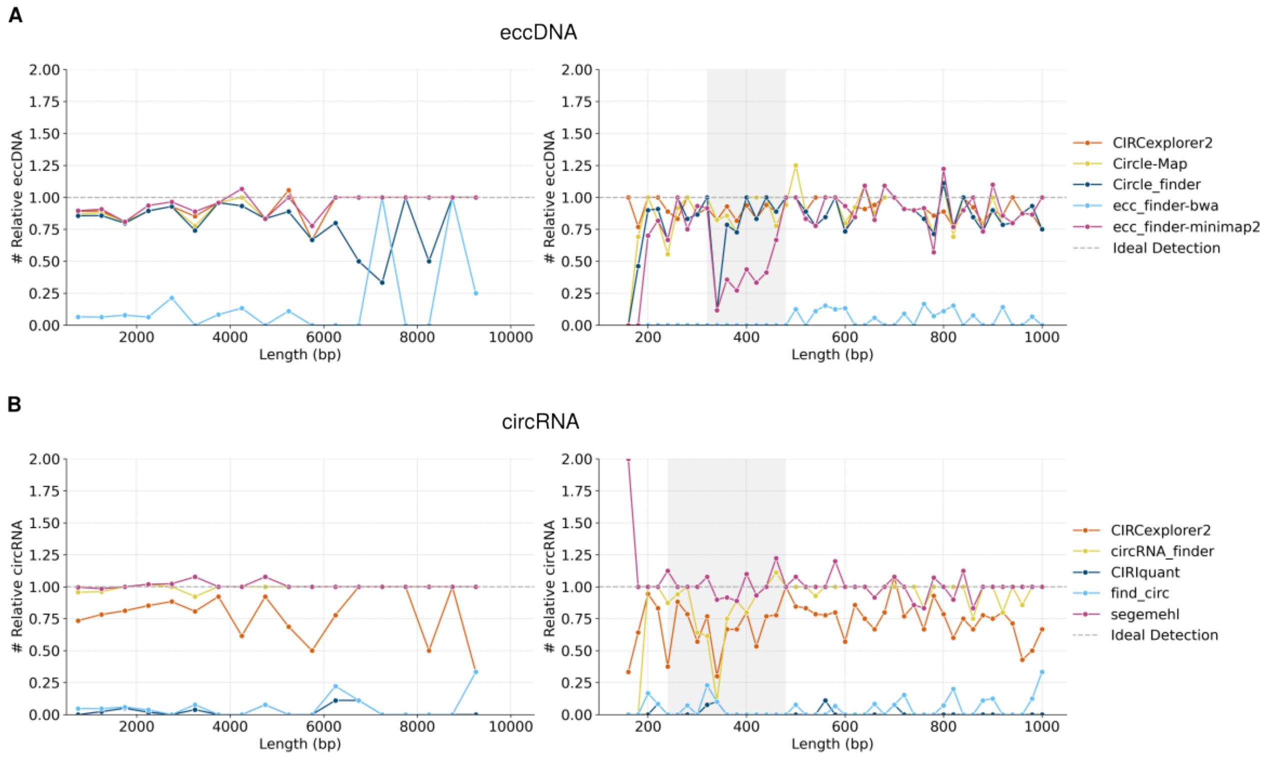

Figure S2. Circular length distribution analysis in *in silico* datasets. Relative circular length distribution of detected eccDNA (**A**) and circRNA (**B**) across all size ranges (**left**) and within the short-length range (**right**) in in silico datasets at coverage x30. A grey-shaded area highlights the length interval where detection performance was lowest (eccDNA: 320-480 bp; circRNA: 240-480 bp). To enhance the clarity of the distribution plots, a sliding window of size 5 was applied to smooth the distribution curves.

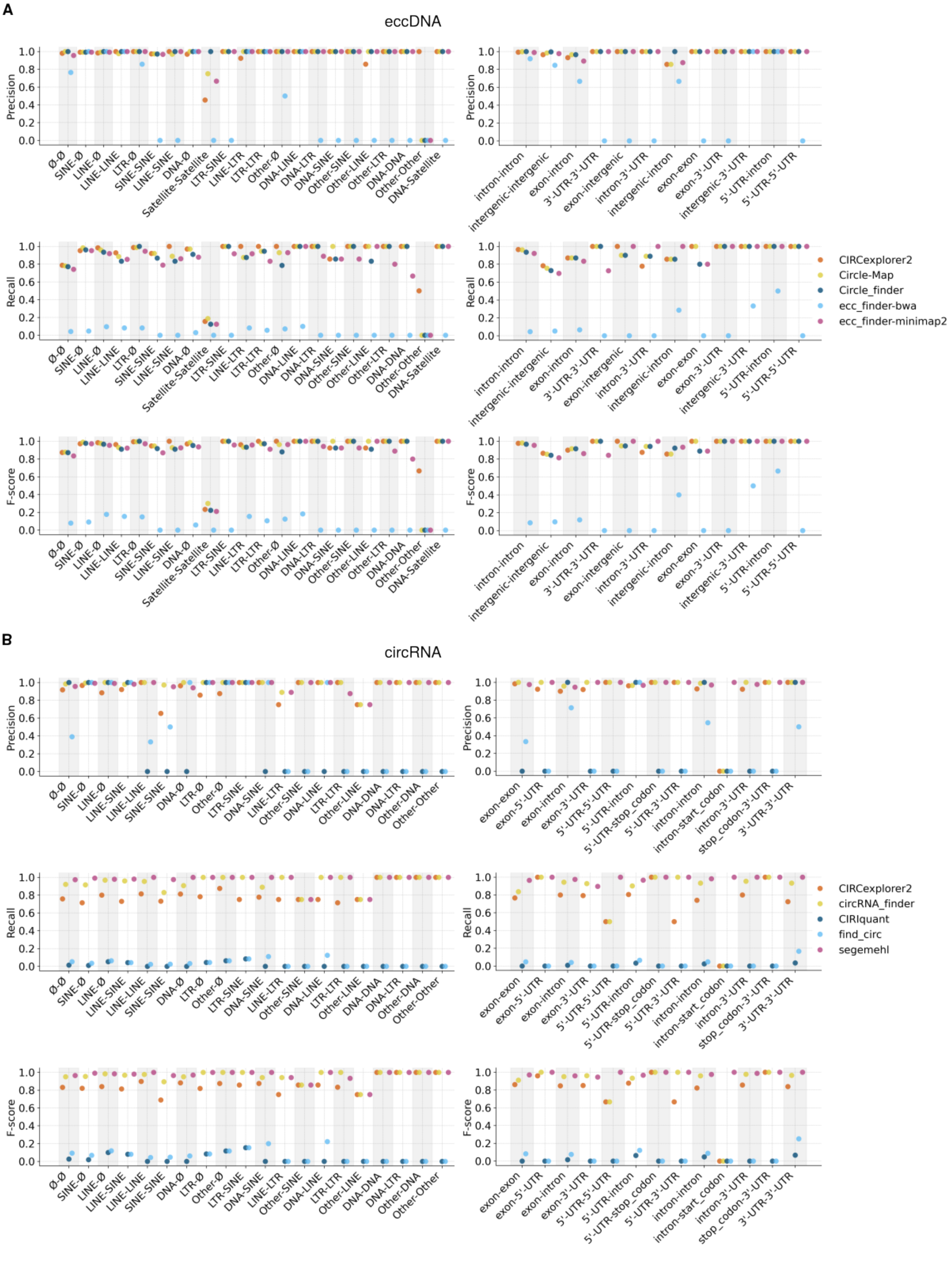

Figure S3. Repeat element analysis and genomic element analysis in *in silico* datasets. Precision (**above**), recall (**middle**) and F-score (**below**) values of all repeat elements (**left**) and genomic features (**right**) associated with detected eccDNA (**A**) and circRNA (**B**) in *in silico* datasets at coverage x30. In circRNAs no intergenic circles were generated and thus the region is not included in this plot.

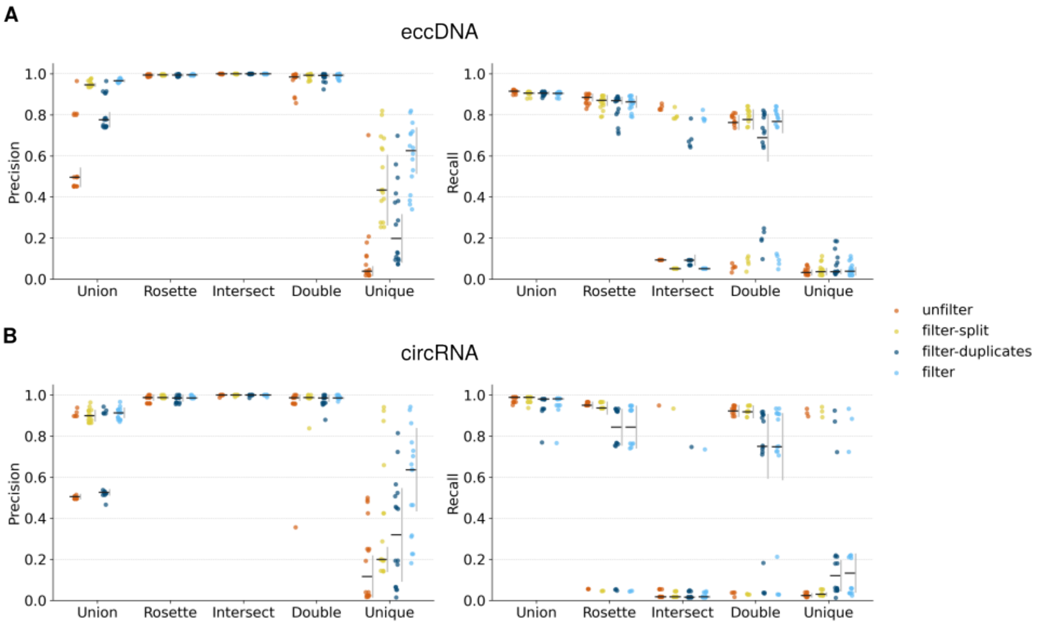

Figure S4. Performance analysis of software combinations for eccDNA and circRNA identification in *in silico* datasets. Strip plot of the F-score of software combination strategies—*Union*, *Rosette*, *Intersect*, *Double*, and *Unique*—were evaluated in *in silico* datasets at coverage x30 under four filtering conditions: *unfilter*, *filter-split*, *filter-duplicates*, and *filter*. Precision (**left**) and recall (**right**) values are shown for eccDNA (**A**) and circRNA (**B**). For each combination-filter pair, the horizontal bar represents the mean value and the vertical gray bar represents the standard deviation.

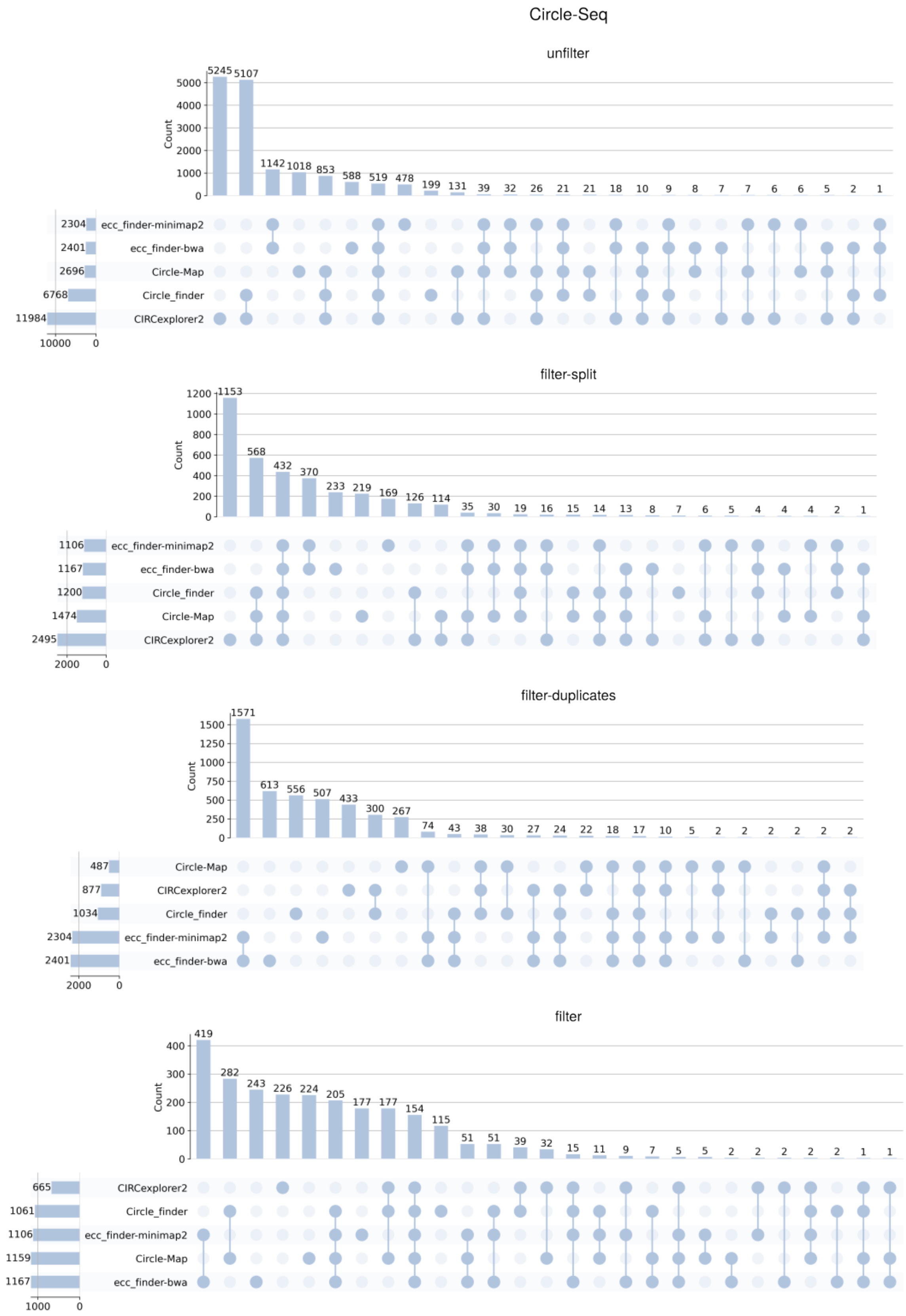

Figure S5. eccDNA detection in Circle-Seq data. UpSet plot showing the intersection of detected eccDNA among different detection tools in four filtering conditions: *unfilter*, *filter-split*, *filter-duplicates*, and *filter*. Each vertical bar represents the size of a specific intersection between tools, while the connected dots below indicate which tools are involved in each intersection. Horizontal bars on the left represent the total number of detections per tool.

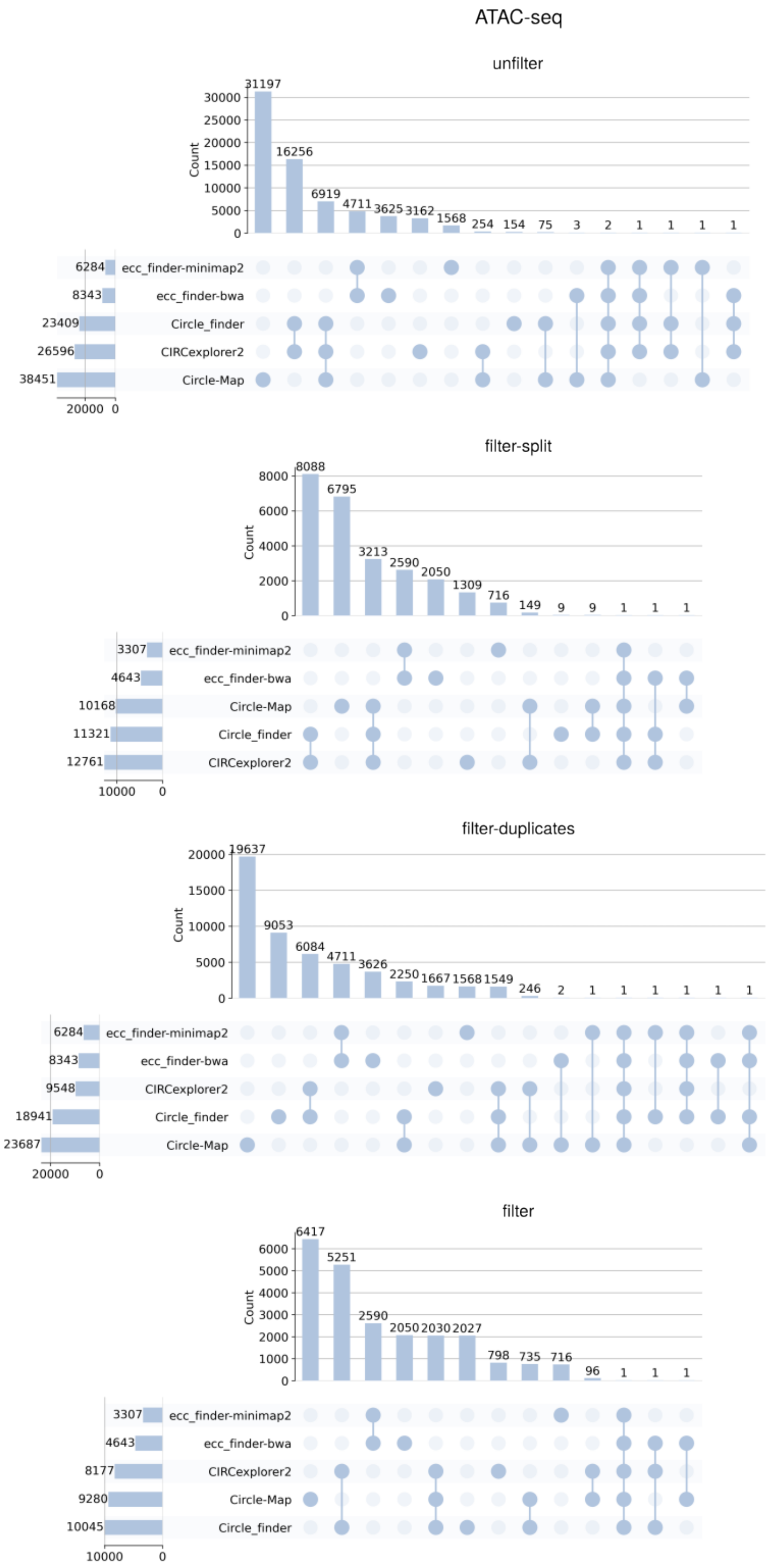

Figure S6. eccDNA detection in ATAC-seq data. UpSet plot showing the intersection of detected eccDNA among different detection tools in four filtering conditions: *unfilter*, filter-split, *filter-duplicates*, and *filter*. Each vertical bar represents the size of a specific intersection between tools, while the connected dots below indicate which tools are involved in each intersection. Horizontal bars on the left represent the total number of detections per tool.

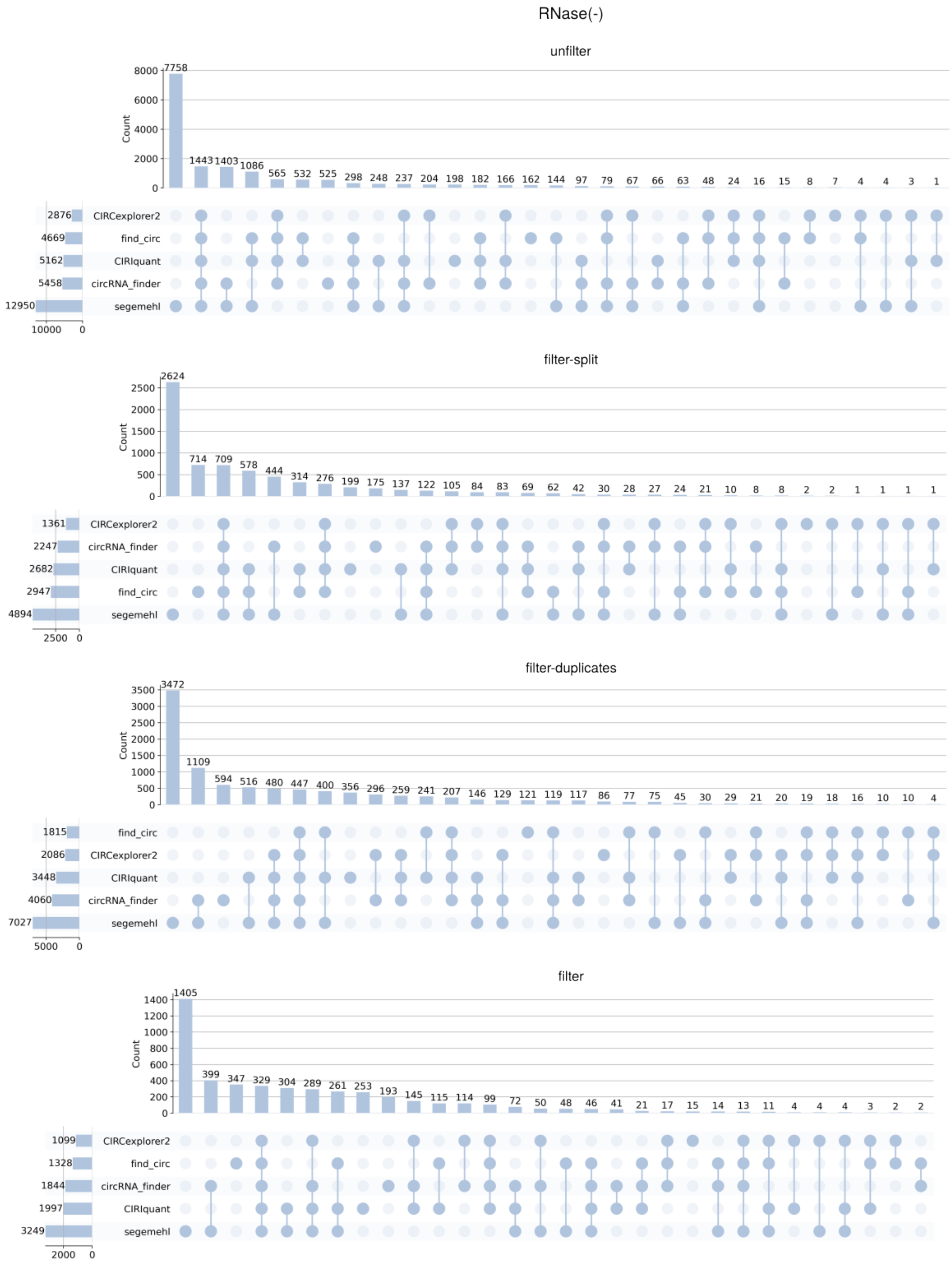

Figure S7. eccDNA detection in RNase(−) data. UpSet plot showing the intersection of detected circRNA among different detection tools in four filtering conditions: *unfilter*, *filter-split*, *filter-duplicates*, and *filter*. Each vertical bar represents the size of a specific intersection between tools, while the connected dots below indicate which tools are involved in each intersection. Horizontal bars on the left represent the total number of detections per tool.

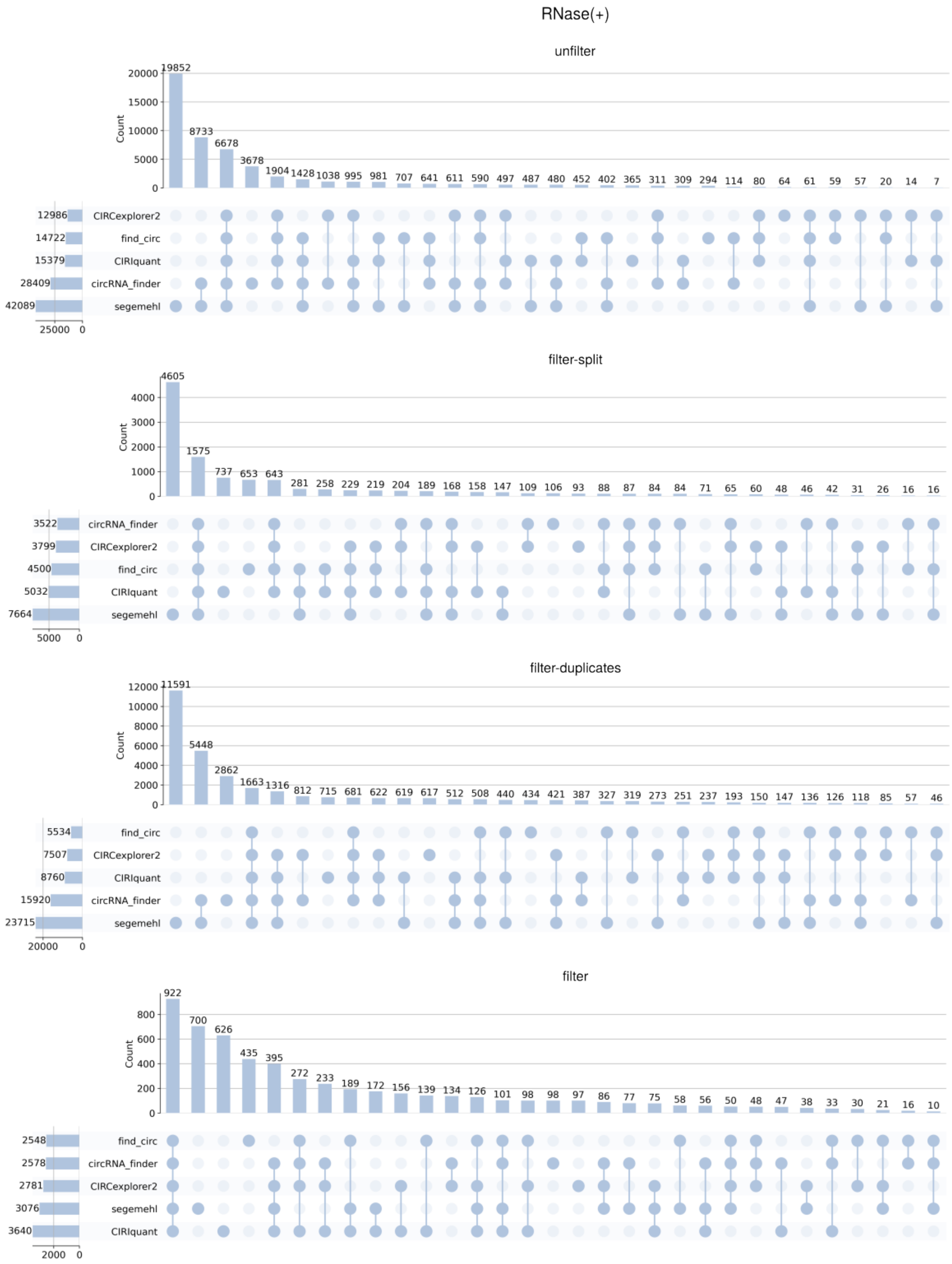

Figure S8. circRNA detection in RNase(+) data. UpSet plot showing the intersection of detected circRNA among different detection tools in four filtering conditions: *unfilter*, *filter-split*, *filter-duplicates*, and *filter*. Each vertical bar represents the size of a specific intersection between tools, while the connected dots below indicate which tools are involved in each intersection. Horizontal bars on the left represent the total number of detections per tool.

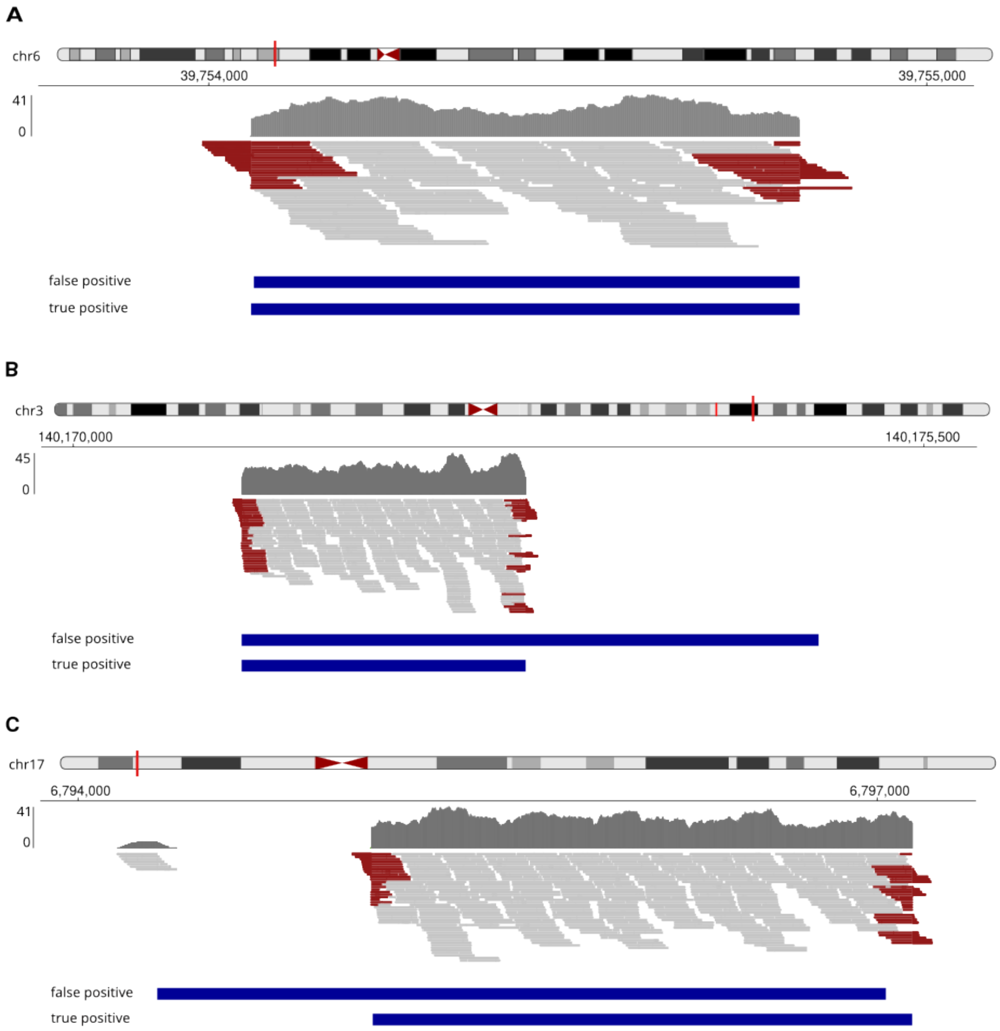

Figure S9. IGV visualization of three eccDNA false positives in *in silico* datasets. Genome browser views (IGV) illustrating three examples of false positive (FP) eccDNAs, each shown alongside their corresponding true positive (TP) circle. (**A**) TP: chr6:39,754,059–39,754,823; FP: chr6:39,754,063–39,754,823. (**B**) TP: chr3:140,171,091–140,172,928; FP: chr3:140,171,091–140,174,822. (**C**) TP: chr3:140,171,091–140,172,928; FP: chr17:6,794,297–6,797,033. Reads marked in red represent the split reads associated with the CJ.

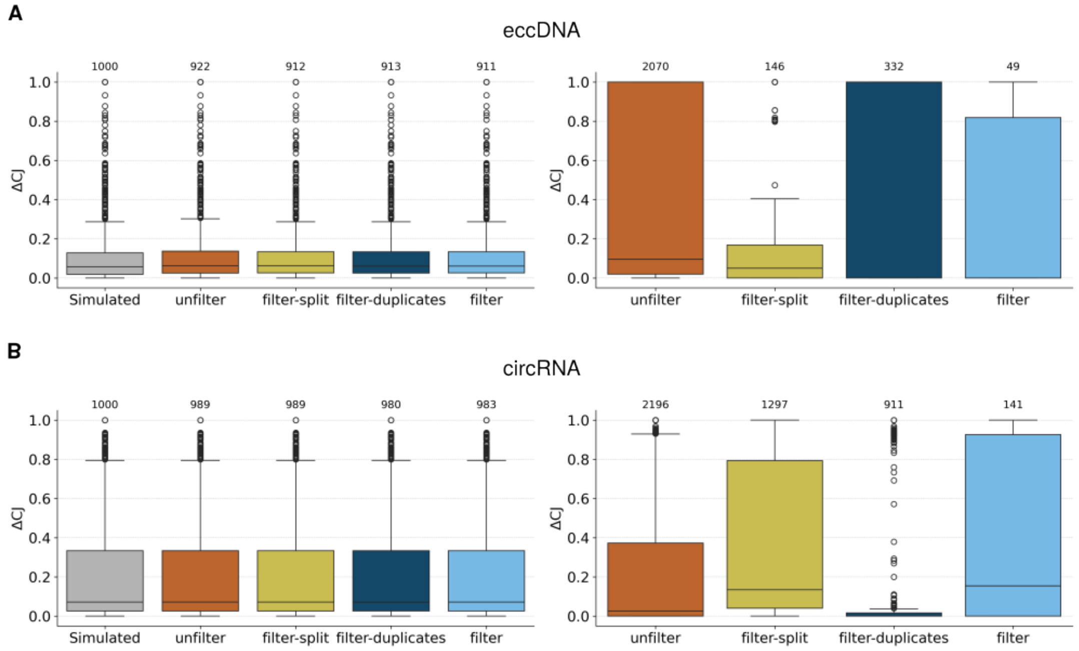

Figure S10. Circular junction nucleotide difference △ CJ in *in silico* datasets. Boxplots of △CJ values of TP (**left**) and FP (**right**) circles for (**A**) eccDNA and (**B**) circRNA in *in silico* datatsets at coverage x30 under *filter* filtering.

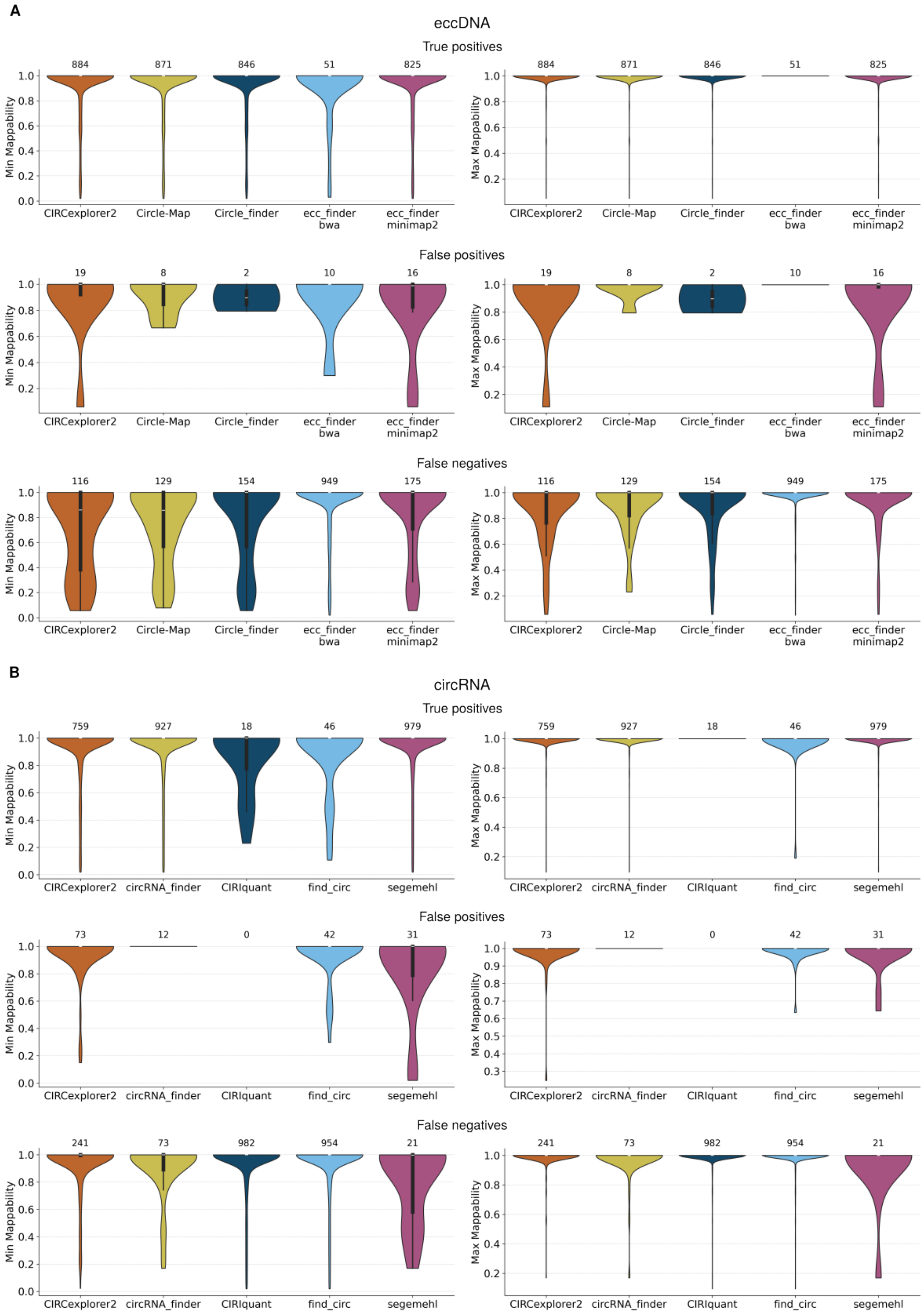

Figure S11. Circular junction mappability in *in silico* datasets. Violinplots of minimun (**left**) and maximun (**right**) mappability for true positive, false positive and false negative (**A**) eccDNA and (**B**) circRNA in *in silico* datatsets at coverage x30 under *filter* filtering.

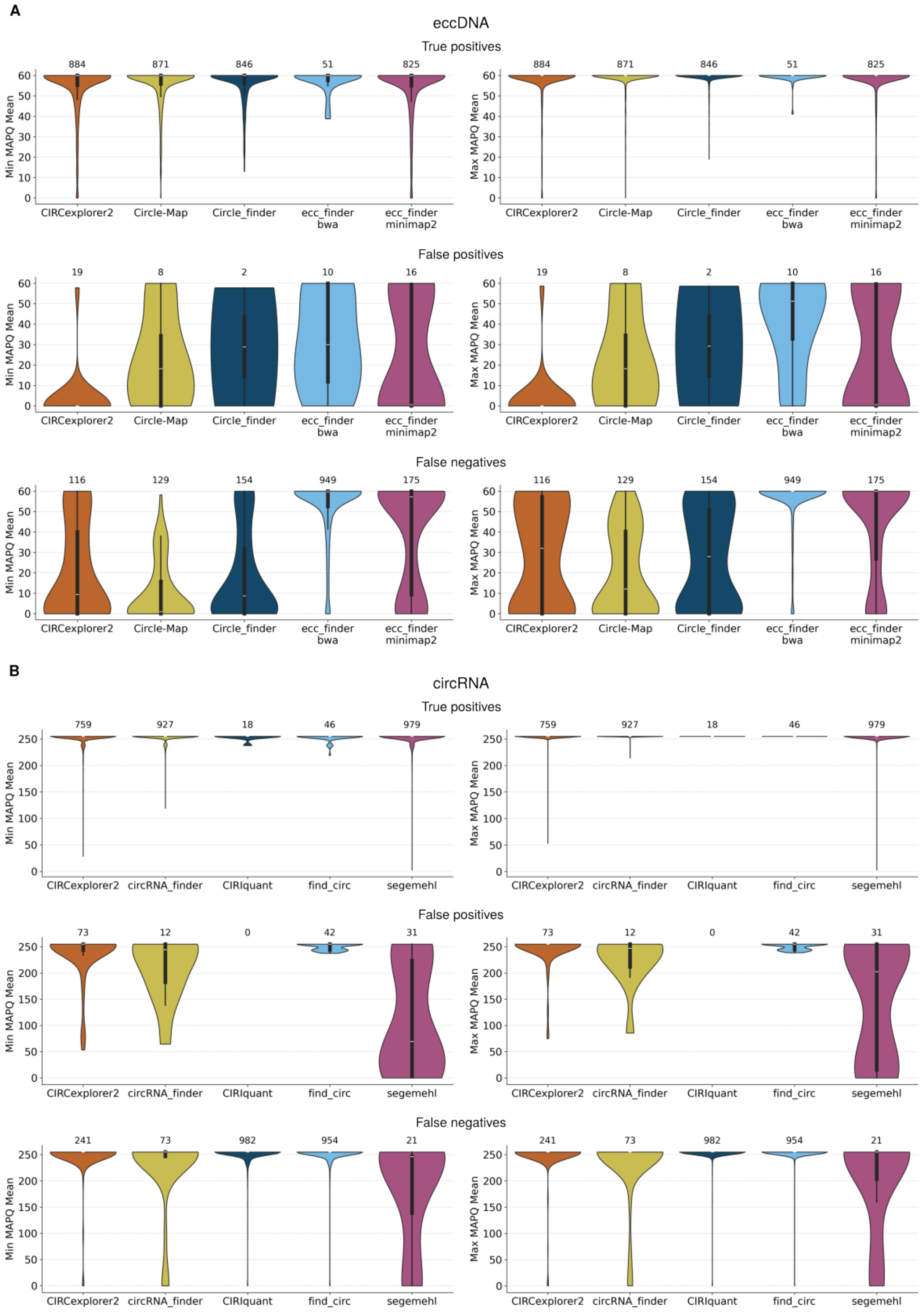

Figure S12. Circular junction read mapping quality (MAPQ) in *in silico* datasets. Violinplots of minimun (**left**) and maximun (**right**) MAPQ mean for true positive, false positive and false negative (**A**) eccDNA and (**B**) circRNA in *in silico* datatsets at coverage x30 under *filter* filtering.

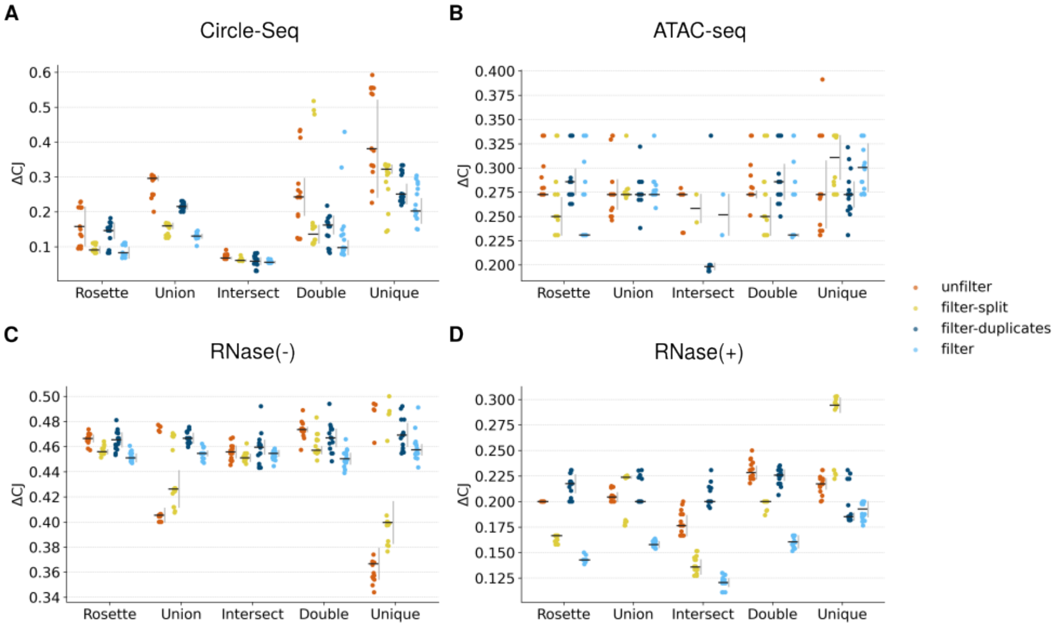

Figure S13. Circular junction nucleotide difference (ΔCJ) of software combinations for eccDNA and circRNA identification in biological datasets. Strip plot of ΔCJ values for different tool combinations—Union, Rosette, Intersect, Double, and Unique—evaluated on biological data. Results are shown for Circle-Seq (**A**) and ATAC-seq (**B**) data for eccDNA, and RNase(-) (**C**) and RNase(+) (**D**) data for circRNA, under four filtering conditions: *unfilter*, *filter-split*, *filter-duplicates*, and *filter*.

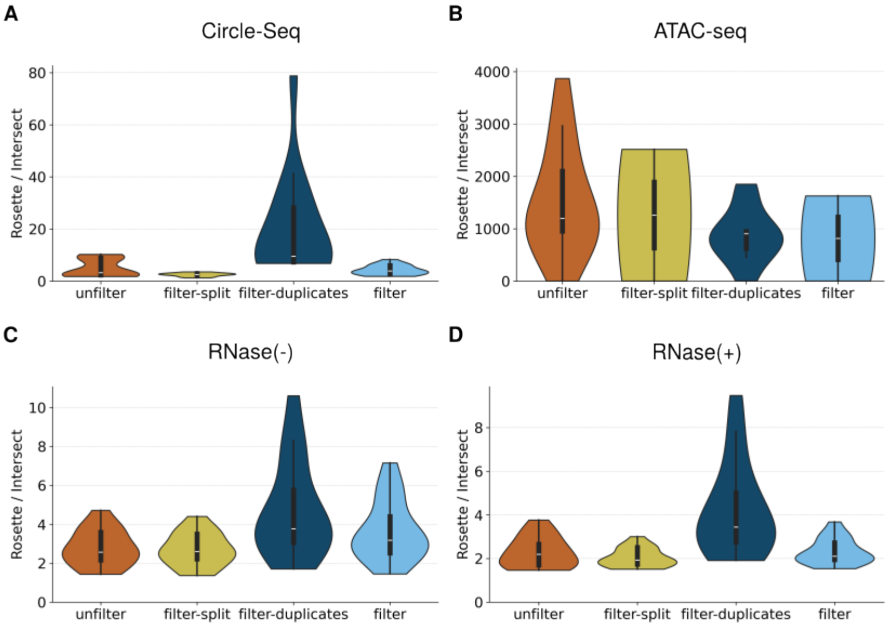

Figure S14. Comparison of detected circles between Rosette and Intersect. Violin plots of the ratio of detected circles in different tool combinations between *Rosette* and *Intersect* in biological data. Results are shown for Circle-Seq (**A**) and ATAC-seq data (**B**) for eccDNA, and RNase(-) (**C**) and RNase(+) (**D**) data for circRNA, under four filtering conditions: *unfilter*, *filter-split*, *filter-duplicates*, and *filter*.

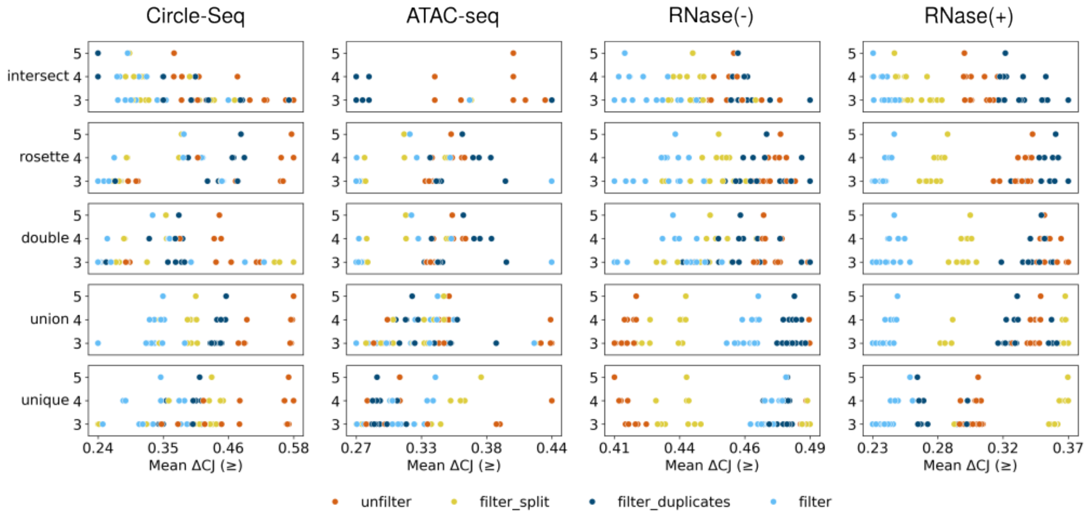

Figure S15. Performance of tool combinations for eccDNA and circRNA in biological datasets. Circular junction nucleotide difference (ΔCJ) mean values are reported, indicating the number of tools combined, across different combinations, filtering conditions and circle enrichment strategies.

This response addresses the manuscript number GIGA-D-25-00306.

Reviewer reports:

Reviewer #1: In "Comparative analysis of eccDNA and circRNA tools shows increased accuracy of tool combination", Zabala and colleagues performed extensive benchmarking of modern tools for eccDNA and circRNA detection from sequencing data, and created innovative ways to compare the performance of these tools despite the lack of a strong truth set for eccDNA or circRNA. The paper is important to the field as comprehensive benchmarks are not common. The authors demonstrated that a more reliable set of calls on eccDNA status can be obtained by combining results of independent callers. The manuscript is well-written, however there are a few points that should be addressed.

Major points

1. EccDNA should be defined from a size or length (bp) early on in the paper, and distinguished from the larger 10kbp+ ecDNA as there can still be confusion in the community of ecDNA research about the type of extrachromosomal DNA being studied when reading studies about eccDNA or ecDNA.

We appreciate the reviewer's suggestion. To clarify the terminology and avoid confusion between eccDNA and ecDNA, we have added an explicit definition in the Introduction. We now specify that in this study, extrachromosomal circular DNA (eccDNA) refers to small circular DNA elements typically ranging from a few hundred base pairs up to approximately 10 kb. We also note that larger oncogene-amplified circles (>10 kb) are referred to as ecDNA and are outside the scope of this work. This clarification appears in the first paragraph of the Introduction section.

Added text in the Introduction:

" In this study, we focus on eccDNA defined as circular DNA elements in the small-to-moderate size range (tens to a few thousand base pairs). We distinguish these from larger, tumor-associated ecDNA elements (commonly >10 kbp) that are typically reported in oncogene amplification studies; because ecDNA and eccDNA differ in size, biogenesis and functional impact, we use the term eccDNA throughout to indicate the smaller class studied here."

2. What exactly is the balance of in-silico and real data in this study in terms of samples? Perhaps a graphical outline of input data can be worked into figure 1? More importantly, do the tools perform differently on in-silico vs. real data? It was not immediately clear if Table 1 and Figure 2 are assessed strictly on simulated data or if they included real data as well.

We thank the reviewer for this helpful suggestion. To clarify the balance between in-silico and biological datasets, we have added a graphical abstract illustrating the study design and data flow, now included as part of Figure 2. This schematic outlines the number and type of datasets analyzed (in-silico and biological) and the workflow followed for each, which varies depending on the data type (e.g. F-score-type analyses are performed in *in silico* data whereas ΔCJ analyses are validated in *in silico* data and then applied for biological data).

In addition, we have revised the captions of tables and figures to explicitly indicate whether the results correspond to in-silico or biological data, and have updated all major titles and subtitles to indicate if the analyses have been performed in *in silico* or biological data This clarification ensures that readers can easily distinguish which analyses were performed on simulated versus experimental datasets.

3. How do these tools perform on samples that have read orientation artifacts? Some PE samples aligned with BWA show an excess of read pairs marked as discordant due to issues with barcode hopping or other technical reasons. Consequently, this can produce read pairs where read1 aligns downstream of read2 on the reference genome, creating outward-facing orientations that mimic circular DNA signatures, whereby the left and right read now have a circular-like / duplication-like orientation. Will these be called as false-positives by the tools? How significant is this problem in real analyzed samples?

This is a very insightful thought that we believe is very relevant to address within the manuscript. Within our first approach, after observing the lack of accuracy of eccDNA detection within centromeric regions, we suspected that circles generated (biologically or artificially) in low-complexity sequences were very likely to be underrepresented because of problems during the alignment process. However, after the comment from the reviewer, and based on the mappability parameter suggested afterwards, we performed an extended analysis based on this parameter, and have observed that (1) *in silico* generated circles that were unmapped (False

Negatives) were present in low mappability regions, and (2) circles with discordant (high) ΔCJ may arise from reads that originate from regions similar to low-mappability regions (i.e. the low-mapping flank in the CJ has low mappability, and therefore the "correct" circle may arise from a region with certain similarity to that low-mappability region, giving rise to that second "copy" circle of which only reads from one flank map correctly. Although this insight suggests a more in-depth analysis, we believe that the extent of the manuscript is already quite broad and further extending it would result in a manuscript with a complex narrative. Nonetheless, due to the relevance of this effect, we will work on this issue in the future, and have already included a section within the *Results* section (*Regional mappability strongly influences the detectability of circular molecules*), briefly commented on other *Results* parts ("*In circle B, the undetected right-side junction region exhibits a lower mappability score (0.470) compared to the mapped region (1), which explains the complete lack of reads on the right side. In circle C, both sides of the junction have high mappability (1.0), but the alignment quality of reads supporting the left side is substantially lower (42.793) compared to the right side (60), leading to a reduced effective read count after MAPQ weighting.*") and commented on it in the *Discussion* (e.g. "*The most pronounced effect occurred with satellite sequences, where detection accuracy declined sharply. This phenomenon may substantially impact eccDNA detection in centromeric regions, which, due to their repetitive nature, could be inaccurately captured, potentially leading to significant functional loss in analyses. This effect is related to the lack of circle detection or lowered detection accuracy in low mappability regions, that has been shown in our analysis.*").

Lastly, the impact of this effect on the biological samples will be discussed in the next question.

4. The delta-CJ metric is useful and important, however the implicit assumption is that both sides of the junction are equally-well mappable is frequently not the case. As the authors point out, the genome is not uniform along its sequence. Can the authors refine this delta-CJ score by incorporating the actual mappability of the sequence at the junction ends? Can a high delta-CJ be explained in some cases by sequence content of one end?

We appreciate the reviewer's insightful comment regarding the assumptions underlying the ΔCJ metric. In response, we have refined the ΔCJ statistic to account for sequence mappability and read-level alignment quality, as suggested. These modifications are now detailed in the revised Methods section.

Specifically, the updated version of ΔCJ includes two complementary adjustments:

- Junction region mappability: We now estimate the expected left/right read split probability based on local mappability scores obtained from UCSC mappability tracks. This correction ensures that regions with different sequence complexities or repetitive content are properly normalized.

- Read-level mapping quality: We further weight each read's contribution according to its mapping quality (MAPQ) to reduce the influence of low-confidence or multi-mapped reads.

The refined metric better reflects the true evidence of circular junctions by integrating genome sequence context and alignment reliability. The mathematical formulation, implementation details, and justification for statistical testing are provided in the revised manuscript.

With respect to the effect of the inclusion of these refined metrics on the analysis, we do observe slight changes in ΔCJ and p values. However, the overall trends and results remain unchanged; which suggests, as observed with in silico data, that most of the circles are detected in high mappability regions, and requires a minimum set of reads with high mapping quality as well. Similarly, as observed in *in silico* data that circles present in low mappability regions are unlikely to be detected, these circles are likely not to be detected in biological data and, therefore, since they are not detected, they do not affect the overall ΔCJ distribution, which consists of detected circles which are in mid-to-high mappability regions.

As suggested in the previous question, including the mappability within the circle detection is important, and suggests further questions like how much is detection of circles arising from low-mappability regions improved from long-read sequencing methods. We will address this in future studies.

5. In the discussion section the authors report that library preparation is one of the most influential factors for both eccDNA and circRNA detection. This reviewer agrees strongly with the claim, based on experience in the field. However, this paper does not rigorously demonstrate this fact. The paper would stand to benefit enormously from a detailed analysis that separates real data by library prep method (perhaps by kit? fragmentation method?) and quantifies and compares tool performance after considering that variable.

We have addressed the effect of library preparation (Circle-seq vs. ATAC-seq for eccDNA and RNAse(-) vs. RNAse(+) for circRNA) by analysing (1) the total number of detected circles and the overlap of circles across tools, (2) the effect on ΔCJ on each tool and method and (3) how tool combination affects ΔCJ. Although studying the effect of the library preparation is complex since we lack a proper "golden truth" or standardized evaluation protocol, we believe that the consistent outcomes on each of these 3 analyses provides enough evidence to suggest how biases in each library preparation method impacts (positively or negatively) the detection of circles. We hope that the performed analysis is sufficiently rigorous and that is correctly reflected in the manuscript.

.

Minor comments:

a. On line 206, the authors state "The circles were simulated with a size distribution ranging from 175 to 10,000bp in a log-normal distribution, with a mean of 1,000 and a standard deviation of 1." Is the value given for the standard deviation correct? If the standard deviation were 1, wouldn't the values be tightly clustered around 1,000 bp? Please clarify whether these refer to the underlying normal distribution parameters or the actual fragment lengths, and provide the correct values for reproducibility.

We thank the reviewer for this observation and agree that clarification is needed. The parameters reported in the manuscript correspond to those of the underlying log-normal distribution, not to the empirical fragment length distribution. Specifically, the circles were simulated using the scipy.stats.lognorm function with parameters s = 1, loc = 0, and scale = 1000, as shown in the code snippet:

```
circle_length = int(lognorm.rvs(s=1, loc=0, scale=1000, size=1))
```

This configuration produces a right-skewed length distribution with most values near 1,000 bp but extending up to ~10,000 bp, consistent with the intended biological range. We have updated the text in the revised manuscript to explicitly state that these are parameters of the log-normal distribution rather than the observed standard deviation of fragment lengths, ensuring clarity and reproducibility.

b. Manuscript methods are very detailed, but could move some non-critical details into supplement (e.g. a paragraph explaining how data was downloaded from SRA is not germane to the paper)

We thank the reviewer for this suggestion. However, based on previous experiences with reviewers in other manuscripts, who complained of the opposite, we decided to keep all methods together to maintain a cohesive narrative that is sustained, and which does not require a break in the reading process. Additionally, GigaScience does not include a word limit, which favors the development of more throughout methods that, we believe, encourage a deeper understanding of the thought process and development of the analyses supported in the manuscript.

c. Grammatical and spelling errors on line 105: "CIRCexplorer2 alignes reads to the reference genome using various aligners and detects non-colinear reads are detected"

We thank the reviewer for catching this error. The sentence has been corrected for clarity and grammar.

d. Grammatical error in section title on line 417

We thank the reviewer for noticing this issue. The section title on line 417 has been corrected to ensure proper grammar and consistency with the rest of the manuscript.

Reviewer #2: The manuscript attempts to benchmark eccDNA and circRNA detection tools and proposes combination strategies with a new metric (ΔCJ). While benchmarking studies can be useful, the present work lacks sufficient methodological novelty, rigor, and biological validation to warrant publication in a high-impact journal. The analysis is superficial in several key aspects, the evaluation design raises concerns about fairness and completeness, and the conclusions are not adequately supported by evidence. Overall, the study falls below the standards expected for GigaScinece.

Major Concerns

1. The study provides little genuine methodological innovation. Tool benchmarking has been repeatedly conducted for both circRNA and eccDNA detection. The only "new" contribution, ΔCJ, is neither adequately validated nor convincingly demonstrated to improve detection accuracy. Tool-combination strategies are already well known in genomics, and here they add minimal incremental value. As presented, the manuscript offers little beyond repackaging existing ideas.

We respectfully disagree with the reviewer's assessment that our study lacks methodological innovation.

First, with respect to eccDNA detection, there is only one previous benchmarking study, which does not evaluate tool combinations. Our work is the first to systematically benchmark eccDNA detection tools within a unified and reproducible framework (Rosette). To deepen on this point, we have performed a thorough comparison on similarities, dissimilarities and innovations produced by this work by to previously published benchmarks in the *Discussion* section.

Second, Rosette represents a novel methodological contribution. It integrates multiple detection tools and harmonizes their outputs within a unified reference framework, allowing for direct, fair, and reproducible cross-tool comparisons. Importantly, our results demonstrate that the Rosette-based combination strategy consistently outperforms individual tools as well as conventional combination approaches—such as simple union or intersection methods (commonly regarded as bona-fide in the circRNA field).

Third, we have refined the ΔCJ metric in the revised version to incorporate junction region mappability and read-level mapping quality, ensuring that it more accurately reflects the true evidence supporting circular junctions. This adjustment directly addresses the reviewer's concerns about the underlying assumptions of ΔCJ and enhances its robustness. Neither this metric, as well as its updated version, nor any similar type of metric have been presented within a circRNA/eccDNA benchmarking paper, and thus introduce a novel aspect on how to evaluate the detection of circles in future benchmarks.

Finally, although tool-combination strategies have been conceptually proposed in genomics, no previous study has quantitatively evaluated their impact on eccDNA or circRNA detection, nor analyzed their performance across different circle lengths and genomic contexts (e.g., satellite regions). These aspects represent both methodological and conceptual advancement beyond previous work.

Together, through these contributions (a novel benchmarking framework (Rosette), the first systematic evaluation of eccDNA tool combinations, and the improved ΔCJ metric) we believe that the current manuscript form provides clear innovation, rigor, and analytical depth that advances the field beyond existing studies.

2. The benchmarking design is problematic. Only a limited subset of tools was included, with no justification for the exclusion of other widely used methods. For example, CIRCexplorer3 (DOI: 10.1016/j.gpb.2019.11.004), has been available for a while. The authors should consider including CIRCexplorer3 in their comparative analysis. Performance metrics for some tools deviate substantially from their published benchmarks, raising questions about whether the tools were run under optimal or even fair conditions. Comparisons between eccDNA and circRNA detection pipelines are conceptually forced, as these classes of molecules have distinct biological properties and sequencing contexts. Without broader tool coverage and rigorous reproducibility, the conclusions cannot be trusted.

We appreciate the reviewer's insightful comments regarding the benchmarking design and tool selection.

We selected the tools included in our benchmark primarily from nf-core pipelines, which provide standardized and well-documented protocols for circular DNA and RNA analysis. This standardization ensures reproducibility and allows for a fair comparison between methods, avoiding inconsistencies caused by user-dependent configurations. For eccDNA, we additionally included two tools outside nf-core due to the limited number of available detection methods at the time of performing the benchmark. We believe that, as the number of tools expands in the near future, the use of nf-core will greatly improve reproducibility and interoperability of the results for future benchmarks and, more generally, more studies in which bona-fide/Rossette strategies are incorporated, and which will warrant more robust results, will be performed.

We acknowledge that other widely used tools, such as CIRCexplorer3 (DOI: 10.1016/j.gpb.2019.11.004), were not part of this study. Our rationale was to focus on pipelines that are fully standardized and reproducible, minimizing variability introduced by differences in default parameters and workflow structures. It is also important to note that CIRCexplorer3 depends on CIRCexplorer2 for detection and can even use its output as input, indicating a strong methodological overlap with tools already included in our benchmark. For this reason we initially decided not to include CIRCexplorer3 within the benchmark and still consider it not sufficient to include it.

Regarding performance discrepancies compared to published results, our findings are consistent for eccDNA. Differences observed for circRNA occur mainly in *in silico* datasets, which were simulated specifically for CIRI, rather than reflecting suboptimal tool execution. All pipelines were executed through nf-core, ensuring optimal configuration and minimizing user-induced variability.

Finally, while we recognize that eccDNA and circRNA represent distinct biological entities and sequencing contexts, we believe that a comparative analysis remains valuable. Both molecule types share similar detection principles based on split-read mapping and circular junction reconstruction. Evaluating their performance side by side provides insights into tool generalizability and highlights methodological limitations that may not be apparent when each class is analyzed independently. For instance, similar effects on the accuracy of the detection linked to circle length and coverage are uncovered in our study, which reflects that technical aspects of tool detection are shared regardless of molecule type, and this aspects are relevant to study to infer potential downstream causal effects.

3. The manuscript reads almost entirely as a technical exercise. The authors do not convincingly demonstrate how their proposed approach leads to more biologically meaningful or validated discoveries. For instance: No downstream analysis is performed to show whether circles identified by ΔCJ or "Rosette" strategies overlap with functional enhancers, promoters, or disease-related elements. The claimed biological implications are left as speculation, with no experimental validation or orthogonal support. As such, the work fails to move beyond technical benchmarking into meaningful biology. These limitations should also be disscussed.

We appreciate the reviewer's comment regarding the biological interpretation of our results. Our study was primarily designed as a technical benchmark, aiming to evaluate the performance, reproducibility, and limitations of current circular DNA and RNA detection tools, rather than to provide comprehensive biological validation.

We acknowledge that downstream analyses of linking detected circles to functional genomic elements such as enhancers, promoters, or disease-associated regions were not included. These analyses were beyond the scope of the present work, as our main objective was to establish a robust and reproducible benchmarking framework that can serve as a foundation for future biology-oriented investigations.

We agree that discussing these limitations is important. In the revised manuscript, we explicitly note that the biological implications of the detected circles remain speculative, and that functional validation through orthogonal experimental approaches or integrative genomics will be required to confirm potential regulatory or disease-related associations. By clarifying this, we aim to position our study as a rigorous technical contribution that enables and informs future biologically driven research, rather than as a study claiming direct biological discoveries.

4. Several claims are overstated relative to the evidence. The assertion that Rosette is the optimal strategy is not convincing, as the reported gains are minimal compared to simpler strategies. The discussion of ΔCJ suggests it prevents false positives, yet no gold-standard validation is provided. Differences between Circle-seq, ATAC-seq, and RNase-treated datasets are described but not rigorously analyzed, leaving open the possibility that observed patterns are driven by sequencing or mapping artifacts.

We respectfully disagree with the characterization that Rosette does not improve upon simpler strategies. In our analyses, Rosette consistently outperforms both union and intersect approaches (the latter commonly regarded as bona fide in circRNA studies), showing clear and reproducible gains in detection performance across multiple datasets. In some cases Rossette has similar performances to the Double strategy, which is not a simple strategy commonly used in other benchmarks either. These improvements are supported by the benchmarking results presented in the manuscript and reflect Rosette's ability to optimize tool complementarity rather than relying on naive overlap criteria.

Regarding ΔCJ, its performance was validated *in silico* using datasets containing a gold-standard set of known circles. While we acknowledge that experimental validation in biological samples is beyond the scope of this

study, these *in silico* results provide robust computational evidence that ΔCJ effectively reduces false positives. We have clarified this point in the revised manuscript, emphasizing that the validation is computational rather than experimental.

We agree that the discussion of Circle-seq, ATAC-seq, and RNase-treated datasets can be strengthened. In the revised version, we now assess mapping quality (MAPQ) and mappability distributions to evaluate whether observed differences arise from sequencing or mapping biases. For instance, similar MAPQ and mappability profiles across RNAse+, RNAse–, ATAC-seq, and Circle-seq datasets support that observed differences are driven by tool-specific behavior, whereas divergent profiles may reflect protocol-dependent effects. We have also considered additional parameters such as read length to contextualize these patterns.

Therefore, we hope that both the improved analyses and the integration of the results and their interpretation within the revised manuscript significantly improve the overall quality of the manuscript and satisfy the requests of the reviewer.
