# Supplementary material for: Comparative analysis of eccDNA and circRNA tools shows increased accuracy of tool combination
Source: Gigascience. 2026 Feb 25;15:giag017. doi: 10.1093/gigascience/giag017 (PMC13154841; doi:10.1093/gigascience/giag017)
Supplement: giag017_Authors_Response_To_Reviewer_Comments_original_submission [file giag017_authors_response_to_reviewer_comments_original_submission.pdf]

Author's Response To Reviewer Comments

This response addresses the manuscript number GIGA-D-25-00306.

Reviewer reports:

Reviewer #1: The authors have adequately addressed all of my comments and concerns, and while there are future directions that should be explored (e.g., the effect of library prep on eccDNA detection, the effect of sequencing artifacts on eccDNA detection), I agree with the authors that those tasks are slightly outside the scope of their existing manuscript. Line 105-106 has a minor grammatical error. I have no further suggestions and recommend the manuscript for publication as the comparisons performed here will help people in the field understand what tools should be used.

We sincerely thank the reviewer for their positive evaluation and for the time and effort invested throughout the entire review process. The minor grammatical error identified in lines 105–106 has been corrected. The reviewer's comments and suggestions have contributed substantially to improving the clarity, robustness, and overall quality of the study. We are pleased that the reviewer finds the comparisons presented here useful for the field and we greatly appreciate the recommendation for publication.

Reviewer #2: This manuscript presents a systematic and carefully executed benchmark of eccDNA and circRNA detection tools using both in silico simulations and biological datasets. The introduction of CircleSim and, in particular, the ΔCJ metric as a proxy for detection quality in the absence of ground truth is a notable conceptual contribution. The study is generally well designed, the analyses are extensive, and the conclusions are largely supported by the data. However, some points need to be addressed to strengthen the manuscript and avoid potential misinterpretation of the results.

1. Provide a concise table summarizing tool versions, aligners, key parameters et al. This would be helpful for readers attempting to replicate the benchmark.

We thank the reviewer for this constructive comment. In response, we have added a concise summary table detailing the tool versions, aligners, pipeline, references, and key detection strategy. This addition is intended to improve transparency and facilitate reproducibility for readers wishing to replicate or build upon our analyses. We thank the reviewer for raising this important point.

2. CircleSlim is a useful contribution, but its biological realism requires clearer justification. Circles are generated uniformly across chromosomes and transcripts, yet real eccDNA and circRNA formation is known to be biased by chromatin state, transcriptional activity, repetitive elements, and genomic architecture. The authors should explicitly discuss which biological biases are not captured by CircleSim, and explain how this affects interpretation of precision/recall values.

We have now explicitly addressed the biological limitations of CircleSim in the manuscript. In particular, we added the following text:

"Despite its relevance for proper in silico analysis, CircleSim faces several limitations related to circle generation dynamics. Our main assumption is that circles are uniformly distributed across the genome or transcriptome; however, biological eccDNA and circRNA formation is known to be biased by chromatin state, transcriptional activity, repetitive elements, and genomic architecture."

3. The conclusion that higher sequencing coverage increases false positives is intriguing but potentially misleading if generalized. The observed decrease in F-score at high coverage appears driven by accumulation of low-confidence split reads, and tool-specific sensitivity to noise. The manuscript should clarify that high coverage per se is not intrinsically detrimental, but rather that current algorithms lack sufficient FP control at high depth without stricter filtering. Reframing this as a tool- and filter-dependent

phenomenon would prevent misinterpretation.

We thank the reviewer for this insightful comment. We have revised the manuscript to clarify that the observed decrease in F-score at high sequencing coverage should not be generalized as an intrinsic problem of high coverage. In particular, we added the following text:

"Delving deeper into the analysis, several structural effects deserve discussion, particularly the issue of FPs. Initially, we observed that coverage substantially influences FP detection in in silico data, a result whose applicability to biological data remains unclear but warrants consideration in analyses. In fact, such decrease in precision at high coverage may appear driven by accumulation of low-confidence split reads, and tool-specific sensitivity to noise. Therefore, high coverage may not per se be intrinsically detrimental, but rather that current algorithms lack sufficient FP control at high depth without stricter filtering."

This framing emphasizes that the phenomenon is tool- and filter-dependent, helping to prevent potential misinterpretation of the results.

Cookie Preference Center

We use cookies which are necessary to make our site work. We may also use additional cookies to analyze, improve and personalize our content and your digital experience. For more information, see our Cookie Policy.

You may choose not to allow some types of cookies. However, blocking some types may impact your experience of our site and the services we are able to offer. See the different category headings below to find out more or change your settings.

Allow all

Manage Consent Preferences

Strictly Necessary Cookies

Always active

These cookies are necessary for the website to function and cannot be switched off in our systems. They are usually only set in response to actions made by you which amount to a request for services, such as setting your privacy preferences, logging in or filling in forms. You can set your browser to block or alert you about these cookies, but some parts of the site will not then work.

Functional Cookies

These cookies enable the website to provide enhanced functionality and personalisation. They may be set by us or by third party providers whose services we have added to our pages. If you do not allow these cookies then some or all of these services may not function properly.

Performance Cookies

These cookies allow us to count visits and traffic sources so we can measure and improve the performance of our site. They help us to know which pages are the most and least popular and see how visitors move around the site.

Targeting Cookies

These cookies may be set through our site by our advertising partners. They may be used by those companies to build a profile of your interests and show you relevant adverts on other sites. If you do not allow these cookies, you will experience less targeted advertising.

Cookie List

Clear

Apply Cancel

Consent Leg.Interest

Confirm my choices

Powe
