# Supplementary material for: Comparative analysis of eccDNA and circRNA tools shows increased accuracy of tool combination
Source: Gigascience. 2026 Feb 25;15:giag017. doi: 10.1093/gigascience/giag017 (PMC13154841; doi:10.1093/gigascience/giag017)
Supplement: giag017_Reviewer_2_Report_Original_submission — Reviewer 2 -- 1/23/2026 [file giag017_reviewer_2_report_original_submission.pdf]

Reviewer Report

Title: Comparative analysis of eccDNA and circRNA tools shows increased accuracy of tool combination

Version: Original Submission **Date:** 1/23/2026

Reviewer name: Reviewer 2

Reviewer Comments to Author:

This manuscript presents a systematic and carefully executed benchmark of eccDNA and circRNA detection tools using both in silico simulations and biological datasets. The introduction of CircleSim and, in particular, the âˆ†CJ metric as a proxy for detection quality in the absence of ground truth is a notable conceptual contribution. The study is generally well designed, the analyses are extensive, and the conclusions are largely supported by the data. However, some points need to be addressed to strengthen the manuscript and avoid potential misinterpretation of the results.

1. Provide a concise table summarizing tool versions, aligners, key parameters et al. This would be helpful for readers attempting to replicate the benchmark.
2. CircleSlim is a useful contribution, but its biological realism requires clearer justification. Circles are generated uniformly across chromosomes and transcripts, yet real eccDNA and circRNA formation is known to be biased by chromatin state, transcriptional activity, repetitive elements, and genomic architecture. The authors should explicitly discuss which biological biases are not captured by CircleSim, and explain how this affects interpretation of precision/recall values.
3. The conclusion that higher sequencing coverage increases false positives is intriguing but potentially misleading if generalized. The observed decrease in F-score at high coverage appears driven by accumulation of low-confidence split reads, and tool-specific sensitivity to noise. The manuscript should clarify that high coverage per se is not intrinsically detrimental, but rather that current algorithms lack sufficient FP control at high depth without stricter filtering. Reframing this as a tool- and filter-dependent phenomenon would prevent misinterpretation.

Methods

Are the methods appropriate to the aims of the study, are they well described, and are necessary controls included? Choose an item.

Conclusions

Are the conclusions adequately supported by the data shown? Choose an item.

Reporting Standards

Does the manuscript adhere to the journal's guidelines on minimum standards of reporting? Choose an item.

Choose an item.

Statistics

Are you able to assess all statistics in the manuscript, including the appropriateness of statistical tests used? Choose an item.

Quality of Written English

Please indicate the quality of language in the manuscript: Choose an item.

Declaration of Competing Interests

Please complete a declaration of competing interests, considering the following questions:

- Have you in the past five years received reimbursements, fees, funding, or salary from an organisation that may in any way gain or lose financially from the publication of this manuscript, either now or in the future?
- Do you hold any stocks or shares in an organisation that may in any way gain or lose financially from the publication of this manuscript, either now or in the future?
- Do you hold or are you currently applying for any patents relating to the content of the manuscript?
- Have you received reimbursements, fees, funding, or salary from an organization that holds or has applied for patents relating to the content of the manuscript?
- Do you have any other financial competing interests?
- Do you have any non-financial competing interests in relation to this paper?

If you can answer no to all of the above, write 'I declare that I have no competing interests' below. If your reply is yes to any, please give details below.

I declare that I have no competing interests

I agree to the open peer review policy of the journal. I understand that my name will be included on my report to the authors and, if the manuscript is accepted for publication, my named report including any attachments I upload will be posted on the website along with the authors' responses. I agree for my report to be made available under an Open Access Creative Commons CC-BY license (http://creativecommons.org/licenses/by/4.0/). I understand that any comments which I do not wish to be included in my named report can be included as confidential comments to the editors, which will not be published.

Choose an item.

To further support our reviewers, we have joined with Publons, where you can gain additional credit to further highlight your hard work (see: https://publons.com/journal/530/gigascience). On publication of this paper, your review will be automatically added to Publons, you can then choose whether or not to claim your Publons credit. I understand this statement.

Yes Choose an item.
